# Giant viruses, giant chimeras: The multiple evolutionary histories of Mimivirus genes

**DOI:** 10.1186/1471-2148-8-12

**Published:** 2008-01-18

**Authors:** David Moreira, Céline Brochier-Armanet

**Affiliations:** 1 Ecologie, Systématique et Evolution, UMR CNRS 8079, Université Paris-Sud, 91405 Orsay Cedex, France; 2 EA 3781 Evolution, Génome, Environnement, Université de Provence, Aix-Marseille I, Marseille, France

## Abstract

**Background:**

Although capable to evolve, viruses are generally considered non-living entities because they are acellular and devoid of metabolism. However, the recent publication of the genome sequence of the Mimivirus, a giant virus that parasitises amoebas, strengthened the idea that viruses should be included in the tree of life. In fact, the first phylogenetic analyses of a few Mimivirus genes that are also present in cellular lineages suggested that it could define an independent branch in the tree of life in addition to the three domains, Bacteria, Archaea and Eucarya.

**Results:**

We tested this hypothesis by carrying out detailed phylogenetic analyses for all the conserved Mimivirus genes that have homologues in cellular organisms. We found no evidence supporting Mimivirus as a new branch in the tree of life. On the contrary, our phylogenetic trees strongly suggest that Mimivirus acquired most of these genes by horizontal gene transfer (HGT) either from its amoebal hosts or from bacteria that parasitise the same hosts. The detection of HGT events involving different eukaryotic donors suggests that the spectrum of hosts of Mimivirus may be larger than currently known.

**Conclusion:**

The large number of genes acquired by Mimivirus from eukaryotic and bacterial sources suggests that HGT has been an important process in the evolution of its genome and the adaptation to parasitism.

## Background

Since their discovery in the last years of the XIX century up to our days, viruses have never ceased to puzzle biologists, especially those studying their evolution. Because of their nature as entities at the border between the living and the non-living, their origin has been the matter of passionate debate. Opinions range between the two extreme "virus-early" and "virus-late" hypotheses. The first postulates that viruses originated before modern cells [1-5]. On the contrary, the second hypothesis proposes that viruses originated either by the escape of genetic material and proteins from cells, or by a dramatic reductive evolution from cellular forms that lost all the "cellular paraphernalia" unnecessary for the parasitic lifestyle [6-8]. As a consequence, the position of viruses within the tree of life is also a subject of disagreement. Whereas some scientists fervently advocate that viruses should have a place in the tree of life [9], many other consider that, being acellular and lacking any kind of carbon and energy metabolism, viruses cannot be properly compared with true living beings (i.e. cellular) and, therefore, do not belong to the tree of life [7,10]. Moreover, there is compelling evidence for the polyphyletic origin of viruses, which further complicates the issue [11].

These debates have recently gained an unprecedented impetus when Raoult and co-workers published the description of the genome of Mimivirus, a giant virus first detected as a parasite of amoeba of the genus *Acanthamoeba*, which is endowed with the biggest viral genome known to date: 1.2 Mbp containing 911 genes [12]. A number of genomic signatures strongly support that Mimivirus belongs to the NCLDV (Nucleo-Cytoplasmic Large ds-DNA Virus) virus family. The mimiviral genome contains genes frequently found in viruses, as those involved in genome replication, but also, and for the first time in a virus, a number of genes coding for proteins involved in transcription and translation. Therefore, at least from the point of view of gene content, Mimivirus appears somehow closer to a typical cell than any other described virus. Moreover, Raoult et al. identified seven mimiviral proteins that have closely related eukaryotic homologues. Their phylogenetic analysis in a multi-protein concatenation supported the emergence of Mimivirus as a sister-group of eukaryotes in a tree including representatives from the three domains of life [12]. Therefore, it was hypothesised that this virus would define a new branch distinct from the three domains of life: Bacteria, Archaea and Eukaryotes [12]. Such an unexpected result had the potential to revolutionise our conceptions on the diversity and evolution of life, up to now based on the tripartite scheme of the three domains [13]. Raoult et al. further suggested that the NCLDV family may have played a role in the origin of eukaryotes [12]. This would agree with previous hypotheses stating that viruses could be at the origin of many eukaryotic genes [3,14] or even the eukaryotic nucleus [15,16].

The study of viral evolution using phylogenetic analysis is most often a difficult task due to several peculiarities of viruses related to their parasitic lifestyle, such as rapid evolutionary rates and the propensity to be involved in horizontal gene transfer (HGT) events [17]. As recently shown [18], the emergence of Mimivirus at the base of the eukaryotic branch in the seven-protein study by Raoult et al. was the result of an accumulation of artefacts due to the simultaneous analysis of several markers that had been profusely exchanged between distant species by HGT [18]. That was the case for two aminoacyl-tRNA synthetases: methyonyl-tRNA (Met-RS) and tyrosyl-tRNA (Tyr-RS) synthetases, which are known to have been intensely affected by HGT [19]. For example, the proteobacterium *Escherichia coli *has a Met-RS of archaeal origin and a Tyr-RS transferred from Gram-positive Firmicutes [18,19]. Therefore, a single species can have aminoacyl-tRNA synthetases with at least two very different evolutionary histories and, consequently, their simultaneous analysis in a multi-protein concatenation will inevitably lead to a wrong phylogenetic tree, especially when a restricted taxonomic sampling is used (i.e., a very small number of species).

When coping with datasets affected by HGT, the most reliable way to avoid artefacts is to carry out independent analyses for each marker. In fact, when independent phylogenetic trees of the proteins used by Raoult et al. were carried out with a rich taxonomic sampling, the results were completely different [18]. Not only HGT events were detected, but Mimivirus did no longer form an independent branch at the base of the eukaryotes, but emerged well nested within them. Moreover, for certain markers such as the Tyr-RS, it branched as a close relative of several species of amoeba [18]. This result showed that, instead of being the ancestral source of eukaryotic genes, Mimivirus has incorporated these genes from its eukaryotic host, the amoeba, into its own genome. While these studies were based on only a handful of genes, we sought here to understand how extensive was the role of HGT in shaping the whole Mimivirus genome, and which were the sources of the transferred genes. These questions were recently addressed using surrogate methods, those that do not require inference of phylogenetic trees, such as the analysis of BLAST scores. Using this approach, Ogata et al. [20] studied the distribution of BLAST scores for 87 Mimivirus ORFs, searched against a database containing homologous sequences from the amoeba *Entamoeba histolytica*, metazoa, fungi and plants. Their analysis showed that only five Mimivirus ORFs (MIMI_L124, MIMI_L469, MIMI_L619, MIMI_R665, and MIMI_L780) are more similar to their *E. histolytica *homologues than to the sequences from other eukaryotic groups. A similar analysis for the entire set of Mimivirus ORFs showed that about 40 of them have eukaryotic or bacterial sequences as best matches [21-23]. However, BLAST searches, as also other surrogate methods, offer only a very rough estimate of the phylogenetic affinity of a gene. Indeed, very often the best BLAST hit does not correspond to the closest evolutionary relative. For example, a BLAST search of the ORF MIMI_R299 (ribonucleotide reductase HI) retrieves several fungi as best hits, whereas phylogenetic analysis supports its relationship with bacterial homologues (Supplementary Figure 28 in Additional data file 2). This can be explained by several factors that can reduce the accuracy of BLAST searches, in particular the heterogeneity of evolutionary rates among species [24]. In that sense, several studies have shown that surrogate methods are clearly inferior to phylogenetic analysis in inferring the evolutionary origin of genes [25,26]. In addition, phylogenetic analyses can provide a very precise identification of the donors of genes acquired by HGT, especially in the case of recent transfer events, and they can also provide statistical measures of the support of the inferred phylogenetic relationships (e.g., bootstrap proportions or Bayesian posterior probabilities). Therefore, we carried out a detailed phylogenetic analysis of all the Mimivirus genome ORFs with well-identified cellular homologues in order to determine whether these genes have a viral or a cellular origin. In the latter case, phylogenetic analyses can be helpful to identify cell-to-Mimivirus HGT events and the corresponding gene donors.

## Results and Discussion

Accurate phylogenetic reconstruction requires a correct degree of conservation among the sequences analysed. Therefore, we focused only on the set of 198 mimiviral proteins ascribed to COG families [27].

### Mimivirus ORF homologues have an extremely patchy taxonomic distribution

**Figure 1:**
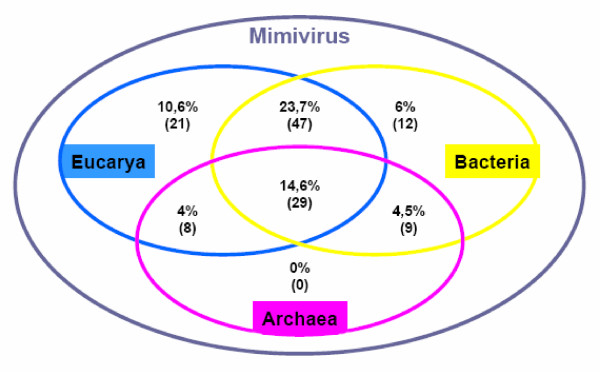
Taxonomic distribution of 128 conserved mimiviral ORFs. The number of homologues in the three domains of life (Eucarya, Archaea and Bacteria) is shown.

For each protein, we retrieved by BLASTP all homologues available in the protein non-redundant (nr) database and studied their distribution in the three domains of life. We considered that homologues of a mimiviral ORF are 'present' in a domain only if they are widely distributed across different phyla of the domain or, at least, in most species of one major phylum (e.g., Metazoa). For 72 ORFs out of the 198 starting proteins, we did not retrieve any clear homologue, some of these ORFs most likely corresponding to erroneous annotations (see Supplementary table 1 in Additional data file 1). Among the remaining 126 ORFs, the most abundant class (Figure 1) was that of ORFs present only in bacteria and eukaryotes (47 ORFs, 37,3%), followed by those present in the three domains (29 ORFs, 23%) or only in eukaryotes (21 ORFs, 16,7%). Smaller proportions of ORFs were found only in bacteria and archaea (9 ORFs, 7%), bacteria (12 ORFs, 9,5%) or archaea and eukaryotes (8 ORFs, 6,5%). A more detailed inspection revealed that the distribution within each domain was in certain cases very unequal. For example, some ORFs were only found in animals (e.g., the glycosyltransferases MIMI_L230 and MIMI_R699), in bacteria and fungi (e.g., the mannosyltransferase MIMI_L373), or in a number of very diverse combinations of taxa (Supplementary table 1 in Additional data file 1).

Interestingly, we found that only a very small fraction of the 198 mimiviral ORFs has homologues in other members of the NCLDV family of viruses, even in those with relatively big genomes. For example, the phycodnaviruses *Emiliania huxleyi *virus 86 (407 kbp, 478 protein coding genes) and *Paramecium bursaria Chlorella *virus 1 (330 kbp, 701 genes) contain only 27 and 28 homologues of the 198 mimiviral ORFs (detected by BLASTP with an E-value threshold of 1e-03), respectively. This represents less than 15% of these 198 ORFs, and could be partially explained by the smaller genome size of those phycodnaviruses. However, the values are significantly smaller than those expected just by the difference in genome size: ~100–150 homologues should be retrieved in these phycodnaviruses. In addition, these data show a very disparate taxonomic distribution of genes among the different viruses of the NCLDV family, a situation that is frequently a clear symptom of HGT [28]. Moreover, our phylogenetic analyses support a viral origin for only 4 ORFs among the 198 mimiviral ORFs studied (the helicase MIMI_L206, the NAD-dependent DNA ligase MIMI_R303, and the two thiol-oxidoreductases MIMI_R368 and MIMI_R596, Additional data file 2). This observation, together with the fact that the mimiviral ORFs that have homologues in the three domains of life are not the dominant class of ORFs in Mimivirus (see above), is extremely difficult to reconcile with the hypothesis that NCLDV viruses may define a fourth major lineage of life (a "fourth domain"). In fact, genomes among the smallest ones found in archaea (e.g. *Nanoarchaeum equitans*, 490 kbp [29]) and bacteria (e.g. *Mycoplasma genitalium*, 580 kbp [30]), which are significantly smaller than the Mimivirus genome, share many more genes with their archaeal and bacterial relatives than the Mimivirus does with the other NCLDV. Moreover, these archaeal and bacterial species with highly reduced genomes have a much larger repertoire of typical cellular genes than the Mimivirus [31,32].

If the "fourth domain" hypothesis [12] was correct, the actual taxonomic distribution shown by the mimiviral ORFs could only be explained by an extremely massive loss of ancestral genes in the different NCLDV viruses but, even in that case, the majority of gene phylogenies should support a clear separation of the mimiviral sequences from those of the other domains. On the contrary, if the "gene acquisition by HGT" hypothesis [18] was correct, the majority of gene phylogenies should support an emergence of the Mimivirus ORFs that have cellular homologues within one of the three domains (according to the specific donor involved in each HGT event). We have explored these two possibilities by detailed phylogenetic analyses, applying Maximum Likelihood (ML) and Bayesian Inference (BI) methods, of all the 126 mimiviral ORFs with cellular homologues and with an adequate degree of sequence conservation.

### Phylogenetic analysis: Mimiviral ORFs of prokaryotic origin

**Figure 2:**
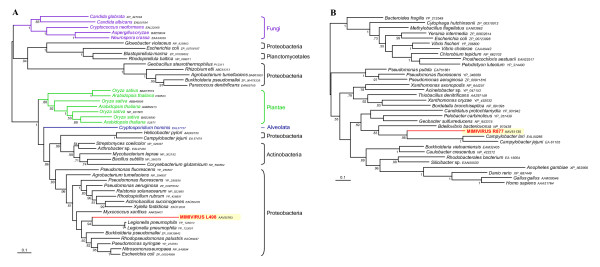
Bayesian phylogenetic trees of (A) the Zn-dependent alcohol dehydrogenase (MIMI_L498) and of (B) the putative outer membrane lipoprotein (MIMI_R877). These trees show HGT events from Proteobacteria that co-exist with Mimivirus within the same amoebal hosts. Numbers at nodes are Bayesian posterior probabilities. Scale bar represents the number of estimated changes per position for a unit of branch length.

We detected a single ORF related to archaeal homologues, the DNA-directed RNA polymerase MIMI_R470 (Supplementary Figure 53 in Additional data file 2), and a much larger number of ORFs related to bacterial sequences. The six mimiviral ORFs MIMI_L432, MIMI_L153, MIMI_R836, MIMI_R852, MIMI_R853, and MIMI_R855 are shared exclusively with bacteria (Figure 1 and Supplementary Table 1 in Additional data file 1). They likely correspond to genes that have been acquired by the virus by HGT from bacteria. Interestingly, the three uncharacterised proteins MIMI_R852, MIMI_R853 and MIMI_R855 show very similar taxonomic distributions across bacteria and similar phylogenies. These three mimiviral ORFs emerge within Cyanobacteria (Supplementary Figures 94, 95 and 96 in Additional data file 2), suggesting a single HGT from a cyanobacterial donor to the Mimivirus. In addition to them, our phylogenetic analyses detected 23 additional genes of bacterial origin among the mimiviral ORFs shared with bacteria and other domains (archaea and/or eukaryotes, Supplementary Figures in Additional data file 2). Some of these are related to homologues from Gram positive Firmicutes: MIMI_L233 (a putative Zn-dependent peptidase) and MIMI_R836 (uncharacterised bacterial protein). The others are closer to proteobacterial sequences: e.g., ORFs MIMI_L477 (a cysteine protease), MIMI_L498 (a Zn-dependent alcohol dehydrogenase), and MIMI_R877 (outer membrane lipoprotein). The source of these bacterial-related ORFs is intriguing. The most appealing possibility is that Mimivirus has acquired them from bacteria that co-infect its same eukaryotic hosts. Indeed, amoebas harbour a variety of intracellular bacteria, including Proteobacteria, Gram positives (both Actinobacteria and Firmicutes), Chlamydiae and Bacteroidetes [33]. Our phylogenetic trees show that several mimiviral ORFs are clearly related to the corresponding homologues from bacterial species that are typical inhabitants of amoeba. That is the case of two of the ORFs cited above: MIMI_L498, related to *Legionella pneumophila *(Figure 2A) and MIMI_R877, related to *Campylobacter *spp. (Figure 2B), which are proteobacteria frequently found in different amoebas, including *Acanthamoeba *[33-35]. A recent article stresses the role that amoebas may have played to facilitate the exchange of genes between different intracellular bacterial species, a phenomenon that might have been important in their adaptation to life within eukaryotic cells [36]. Our results suggest that the situation may be even more complex, since the intracellular bacteria appear to have transferred genes also to the mimiviral genome. Some of these genes are probably involved in the parasitic adaptations of the Mimivirus, such as the *Campylobacter*-like outer membrane lipoprotein cited above (MIMI_R877).

### Mimiviral ORFs of eukaryotic origin: amoebas and evidence for unknown hosts

**Figure 3:**
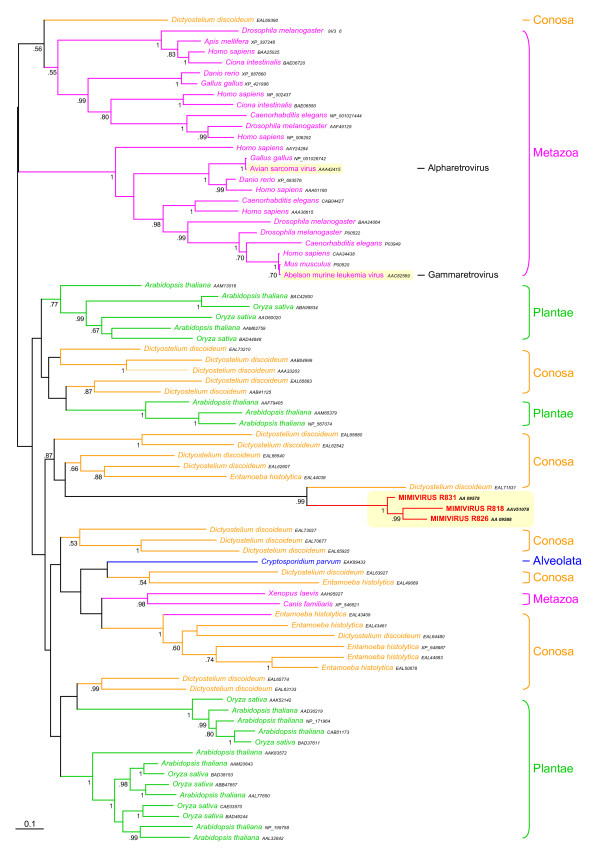
Bayesian phylogenetic tree of three serine/threonine protein kinases (MIMI_R818, MIMI_R826, MIMI_R831). The tree shows one gene acquisition by Mimivirus from its host, followed by two duplication events in the mimiviral lineage. Numbers at nodes are Bayesian posterior probabilities. Scale bar represents the number of estimated changes per position for a unit of branch length.

Most of the 126 mimiviral ORFs that have homologues in cellular species useful for phylogenetic analysis can be found in eukaryotes (105 ORFs, 21 of them being absent from prokaryotes, Figure 1). Our phylogenetic analyses inferred a eukaryotic origin for 60 of these ORFs. Interestingly, several ORFs of eukaryotic origin are closely related to homologues found in different amoebas, as in the case of MIMI_L124 (tyrosyl-tRNA synthetase) already reported [18]. We inferred a clear amoebal origin also for MIMI_R214 (RAS family GTPase), MIMI_L254 (heat shock protein HSP70), MIMI_L258 (thymidine kinase), MIMI_R259 (DUF549 domain-containing protein), MIMI_L300 (endo/excinuclease), MIMI_L394 (HD superfamily phosphohydrolase), MIMI_R405 (tRNA uracil-5-methyltransferase), MIMI_L444 (ADP-ribosylglycohydrolase), MIMI_R464 (translation initiation factor SUI1), MIMI_R528 (unknown protein), MIMI_R818, MIMI_R826 and MIMI_R831 (three paralogous serine/threonine protein kinases, Figure 3). To sum up, our trees support that ~10% of the mimiviral ORFs with eukaryotic homologues were acquired from amoeba. These ORFs are involved in a variety of processes and, in some cases, their acquisition by HGT was followed by duplication events, such as the kinases MIMI_R818, MIMI_R826 and MIMI_R836 (Figure 3). Certain mimiviral ORFs are exclusively shared by this virus and its amoebal hosts and they represent probable additional host-to-virus HGT events. This is the case of the 16 mimiviral ORFs proteins containing the FNIP motif detected by Song et al. [37]. It is important to note that all these ORFs of amoebal origin represent a minimal number since others might also have an amoebal origin but did not produce well resolved trees, a problem that can be due to different causes: lack of phylogenetic signal, small number of positions useful for phylogenetic analyses of several ORFs, tree reconstruction artefacts due to unequal evolutionary rates among taxa, and/or missing data concerning amoebas (for example, there is no complete genome sequence available at present for any *Acanthamoeba *species).

**Figure 4:**
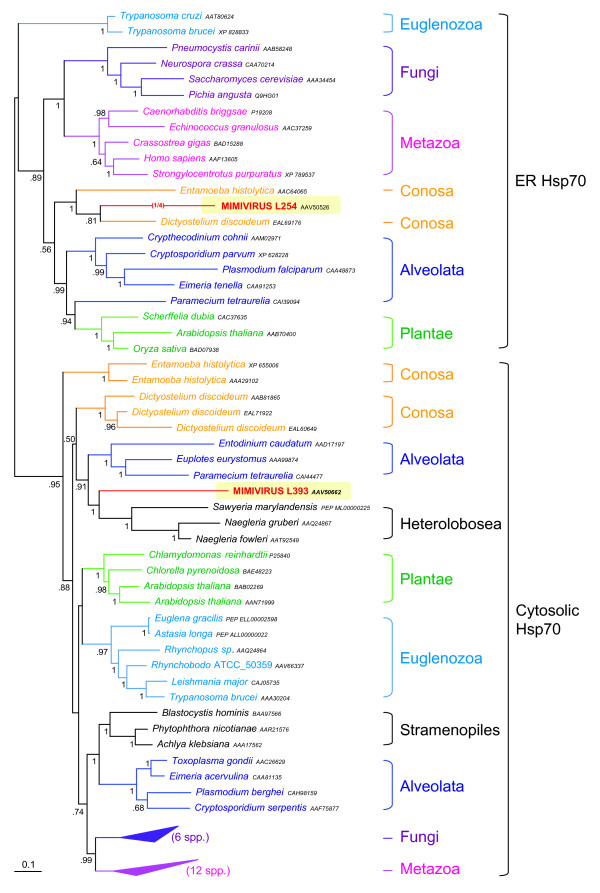
Bayesian phylogenetic tree of a cytosolic- and an endoplasmic reticulum (ER)-type HSP70 heat shock protein (MIMI_L254, and MIMI_L393). The tree shows the eukaryotic origin of the two mimiviral HSP70 by independent HGT from two distant eukaryotic groups (Amoebozoa and Heterolobosea). Numbers at nodes are Bayesian posterior probabilities. Scale bar represents the number of estimated changes per position for a unit of branch length.

In addition to all the ORFs likely acquired by HGT from the amoebal hosts, we detected a few ones that support a close phylogenetic relationship between the Mimivirus and eukaryotes unrelated to the Amoebozoa. The phylogenetic analysis of the two HSP70 homologues found in Mimivirus is a remarkable example. Whereas MIMI_L254, an endoplasmic reticulum-type HSP70, is strongly related to amoebozoan homologues, the cytosolic-type HSP70 MIMI_L393 is clearly related to species of the genera *Naegleria *and *Sawyeria *(Figure 4). These species are flagellated amoebas, many of them parasitic, which belong to the Heterolobosea [38,39]. This group is possibly related to the Euglenozoa, forming a large assemblage called the Discicristata, very distant from Amoebozoa [40,41]. The presence of giant virus-like particles in the cytoplasm of *Naegleria fowleri *was already noticed in the 1970s [42,43], and it was recently hypothesised that these particles might be related to Mimivirus [44]. Our detection of a mimiviral ORF phylogenetically related to homologues from *Naegleria *and *Sawyeria *strongly supports the hypothesis that Mimivirus can also infect these amoeboid species, even if they are very distant from the hosts, such as *Acanthamoeba *spp., known up to date.

**Figure 5:**
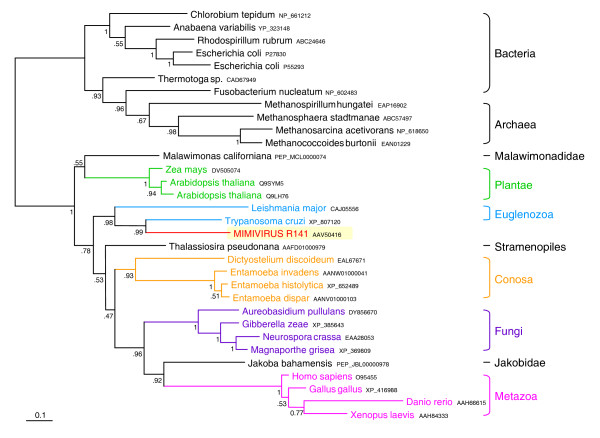
Bayesian phylogenetic tree of the GDP mannose 4,6-dehydratase (MIMI_R141). The tree shows a case of gene acquisition by Mimivirus from a euglenozoan donor. Numbers at nodes are Bayesian posterior probabilities. Scale bar represents the number of estimated changes per position for a unit of branch length.

In addition to this ORF, we detected three other ORFs (MIMI_R141, MIMI_L605, and MIMI_L615, see Supplementary Figures 7, 70 and 73 in Additional data file 2) that are related to homologues from different protist species belonging to the Euglenozoa, more specifically to the Kinetoplastida, which are flagellates with both free-living and parasitic members, such as *Trypanosoma *and *Leishmania*. Since the heterolobosean flagellate amoebas, as *Naegleria*, are likely related to the Euglenozoa (see above), the possibility exists that these ORFs have also been acquired from *Naegleria *relatives but that we could only detect a phylogenetic affinity with the Kinetoplastida because the corresponding sequences from *Naegleria *are not available in current databases. That could also be the case for the ORFs that branch as sisters of the kinetoplastid sequences: MIMI_L605 (peptidylprolyl isomerase) and MIMI_L615 (phosphatidylinositol kinase). However, for the ORF MIMI_R141 (dTDP-D-glucose 4,6-dehydratase), our phylogenetic analyses strongly support the emergence of Mimivirus within the Kinetoplastida, indicating that it acquired this ORF from a kinetoplastid species (Figure 5). This suggests that not only protists with amoeboid cell structures, but also typical flagellates such as the kinetoplastids may be hosts of mimiviruses. Nevertheless, we cannot discard the possibility that HGT from these flagellates to Mimivirus occurred within amoebas since kinetoplastid parasites (such as *Perkinsiella amoebae*) have been detected in several amoebal species [45]. As in the case of several mimiviral ORFs closely related to homologues from parasitic bacteria (see above), these flagellates may have transferred genes to the Mimivirus infecting the same amoebal hosts. A less parsimonious alternative hypothesis would be that these ORFs have been transferred to Mimivirus by an unidentified third partner, for example viruses infecting kinetoplastids and heterolobosea that could have recombined with Mimivirus. However, the fact that particles very similar to Mimivirus have been observed in *Naegleria *supports the hypothesis of direct gene acquisition at least from heterolobosean hosts.

### The amoebas as complex ecosystems promoting HGT

The number of bacterial species that have been characterised as stable inhabitants of diverse amoebas seems to be far from being completely known [33,46]. As commented above, the promiscuity of these bacterial species within amoebas may have facilitated HGT and the adaptation to parasitic lifestyles [36]. Several eukaryotic parasites, such as the kinetoplastid *Perkinsiella*, can also be found in amoebas [45]. Moreover, there is increasing evidence that the use of amoebas as a reservoir can be a key factor in the selection of virulent strains of eukaryotic parasites of mammals, in particular several pathogenic dimorphic fungi [47,48]. Mimiviruses infecting amoebas can thus coexist in a confined environment with a variety of other parasites. In addition to the DNA from the amoebal host, the sporadic cell lysis of these parasites provides DNA that can be a source of new genes for the virus. The acquisition of genes from the host and from its bacterial and eukaryotic parasites may be significant in the development of virulence traits of the virus, but also in its opportunistic pre-adaptation to alternative hosts, including humans. This has been shown to be an important process in other pathogens. For example, the parasitic bacterium *L. pneumophila *has acquired by HGT several eukaryotic genes involved in a variety of cell functions, in particular two serine/threonine protein kinases [49]. It has been shown in several pathogens that these protein kinases are responsible of inhibiting phagosome-lysosome fusion, allowing intracellular survival, but also of disrupting the host defence by interfering with the eukaryotic signal transduction pathways [50,51]. Interestingly, Mimivirus possesses three serine/threonine protein kinases (MIMI_R818, MIMI_R826 and MIMI_R831) that have been acquired from its amoebal hosts (see above and Figure 3). This is also the case for the RAS GTPase MIMI_R214. The presence of these proteins suggests that Mimivirus can regulate the host cell cycle to its benefit. This is an example of the crucial role that HGT may have had in the evolution of the virulence strategy of Mimivirus.

## Conclusion

Most of the genes in Mimivirus with homologues in cellular organisms appear to have been acquired by HGT to the virus. This strongly supports that Mimivirus does not define a new domain of life. Mimivirus certainly acquired most of these genes either from its eukaryotic hosts or from other parasites coexisting in the same host. In that sense, this virus appears to have followed a similar strategy as other parasites to interfere with host cellular processes through the modification and expression of genes acquired from the host by HGT. Our data also suggest that the primary hosts for Mimivirus are the Amoebozoa, as most of the mimiviral ORFs of eukaryotic origin with a well resolved phylogeny are closely related to homologues from this group. Nevertheless, we detect HGT from putative alternative hosts, such as heterolobosea (e.g., *Naegleria*) and kinetoplastids. Despite the fact that Mimivirus appears to have the capacity to infect also humans [46,52,53], we identified only a single case of a probable gene acquisition from animals, the glycosyltransferases MIMI_L230 and MIMI_R699 (Supplementary Figure 15 in Additional data file 2). This small number suggests that the recently reported human infection by this virus may be a relatively rare or very recent event.

## Methods

### Phylogenetic reconstruction methods

Homologues for each Mimivirus ORF were retrieved from the NCBI protein non-redundant data base after identification by BLAST [54]. The sequences were aligned with CLUSTALW [55] and the alignment was manually refined with the program ED of the MUST package [56]. Regions where homology between sites was doubtful were removed before the phylogenetic analysis.

Data sets were analysed by Maximum likelihood (ML) using the JTT model with a Γ law (4 rate categories) and a proportion of invariant sites using the program PHYML [57]. To asses the topologies found by ML, several data sets were also analysed with Bayesian methods using the program MrBAYES 3 with a mixed substitution model and a Γ law (6 rate categories) and a proportion of invariant sites to take among-site rate variation into account [58]. The Markov chain Monte Carlo search was run with 4 chains for 1,000,000 generations, with trees being sampled every 100 generations (the first 2,500 trees were discarded as "burnin").

## Authors' contributions

DM and CB-A collaboratively designed the study, carried out the phylogenetic analyses, interpreted the results, and wrote the paper. Both authors read and approved the final manuscript.

## Supplementary Material

Additional data file 1 Table with details on all Mimivirus ORFs studied in this work. Click here for file

Additional data file 2 Maximum likelihood phylogenetic trees for all the conserved Mimivirus ORFs with cellular homologue Click here for file
