# Supplementary material for: Giant viruses, giant chimeras: The multiple evolutionary histories of Mimivirus genes
Source: BMC Evol Biol. 2008 Jan 18;8:12. doi: 10.1186/1471-2148-8-12 (PMC2263039; doi:10.1186/1471-2148-8-12)
Supplement: Additional data file 2 — Maximum likelihood phylogenetic trees for all the conserved Mimivirus ORFs with cellular homologue [file 1471-2148-8-12-S2.pdf]

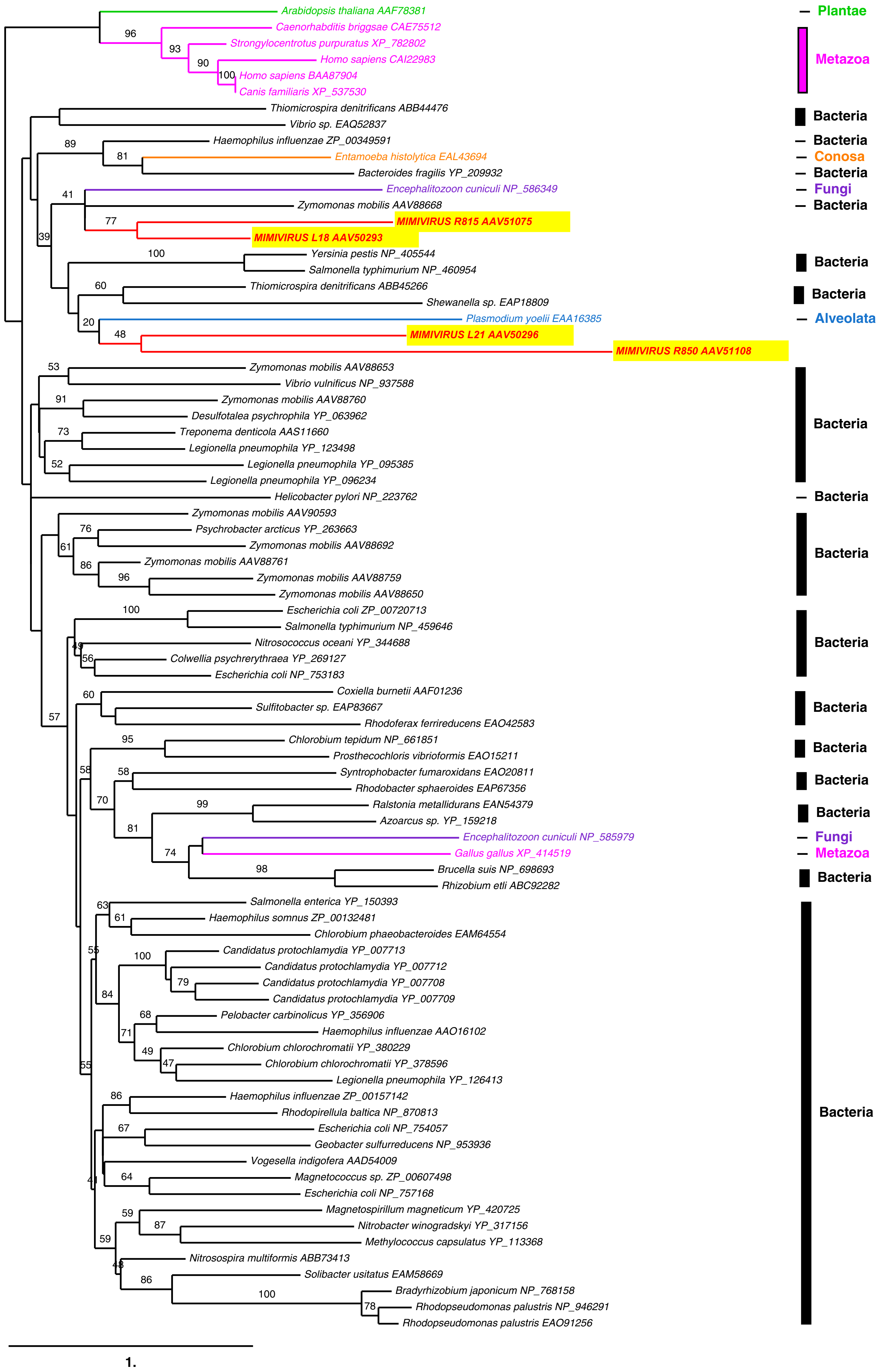

Figure 1: L18/L21/R815/R850 (FOG: TPR repeat, SEL1 subfamily)

(82 sequences, 139 positions)

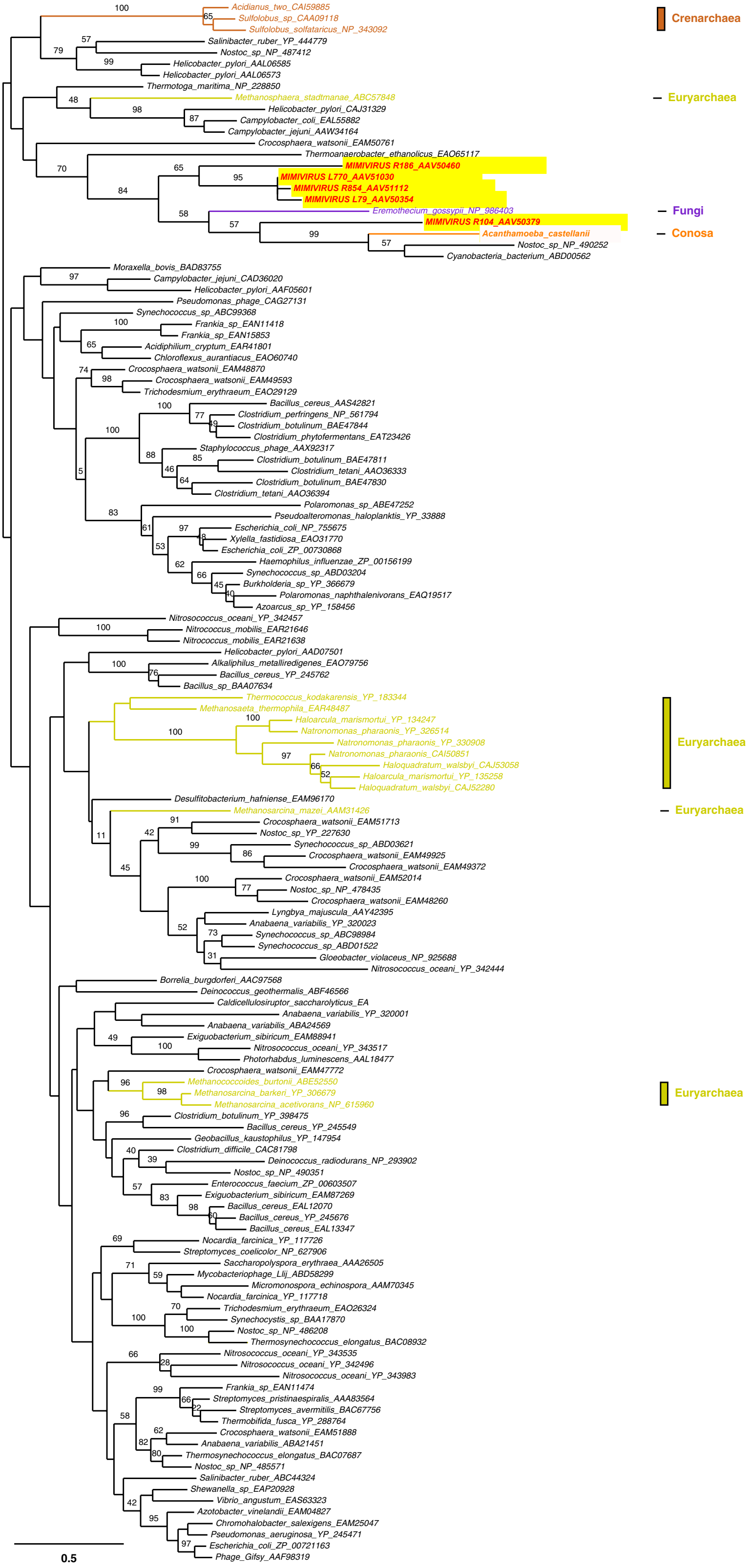

Figure 2: L79/R104/R186/L770/R854 (Transposase and inactivated derivates)
(138 sequences, 117 positions)

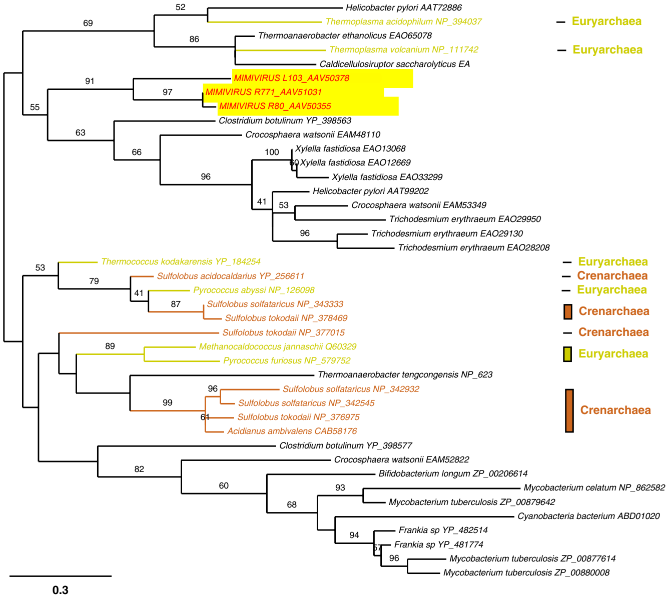

Figure 3: r80/L103/R771 (predicted site-specific integrase-resolvase)
(41 sequences, 71 positions)

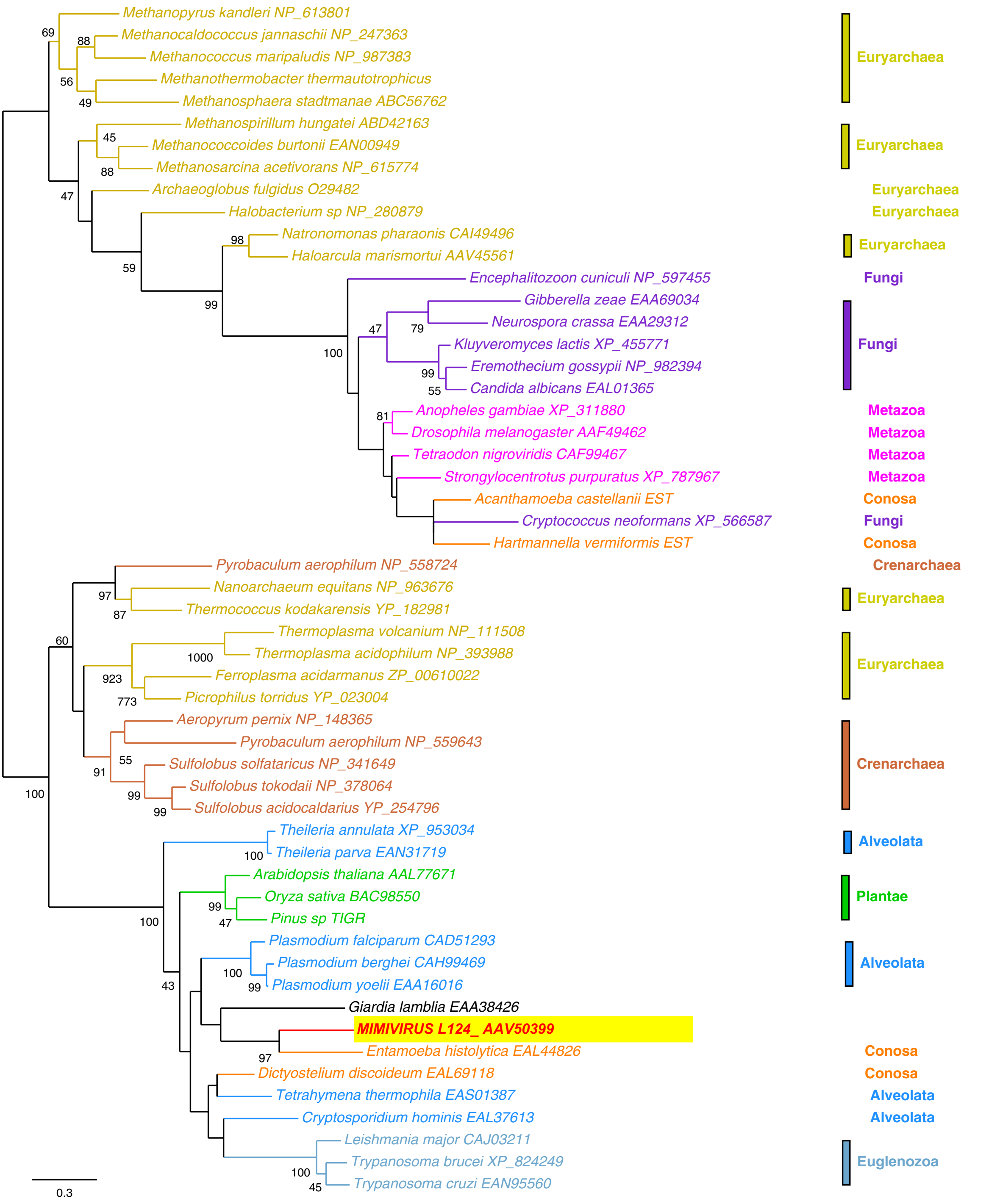

Figure 4:
L124 (Tyrosyl-tRNA synthetase)
(54 sequences, 154 positions)

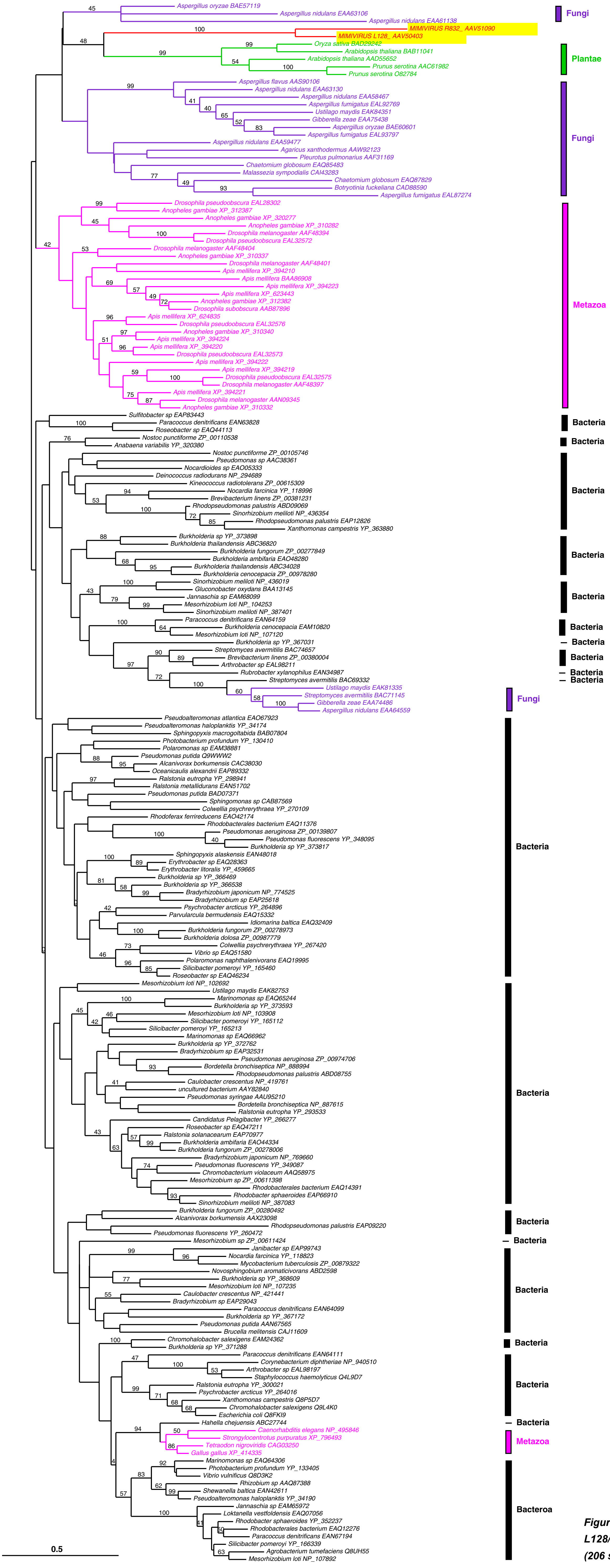

Figure 5:
L128/R832 (Choline dehydrogenase)
(206 sequences, 176 positions)

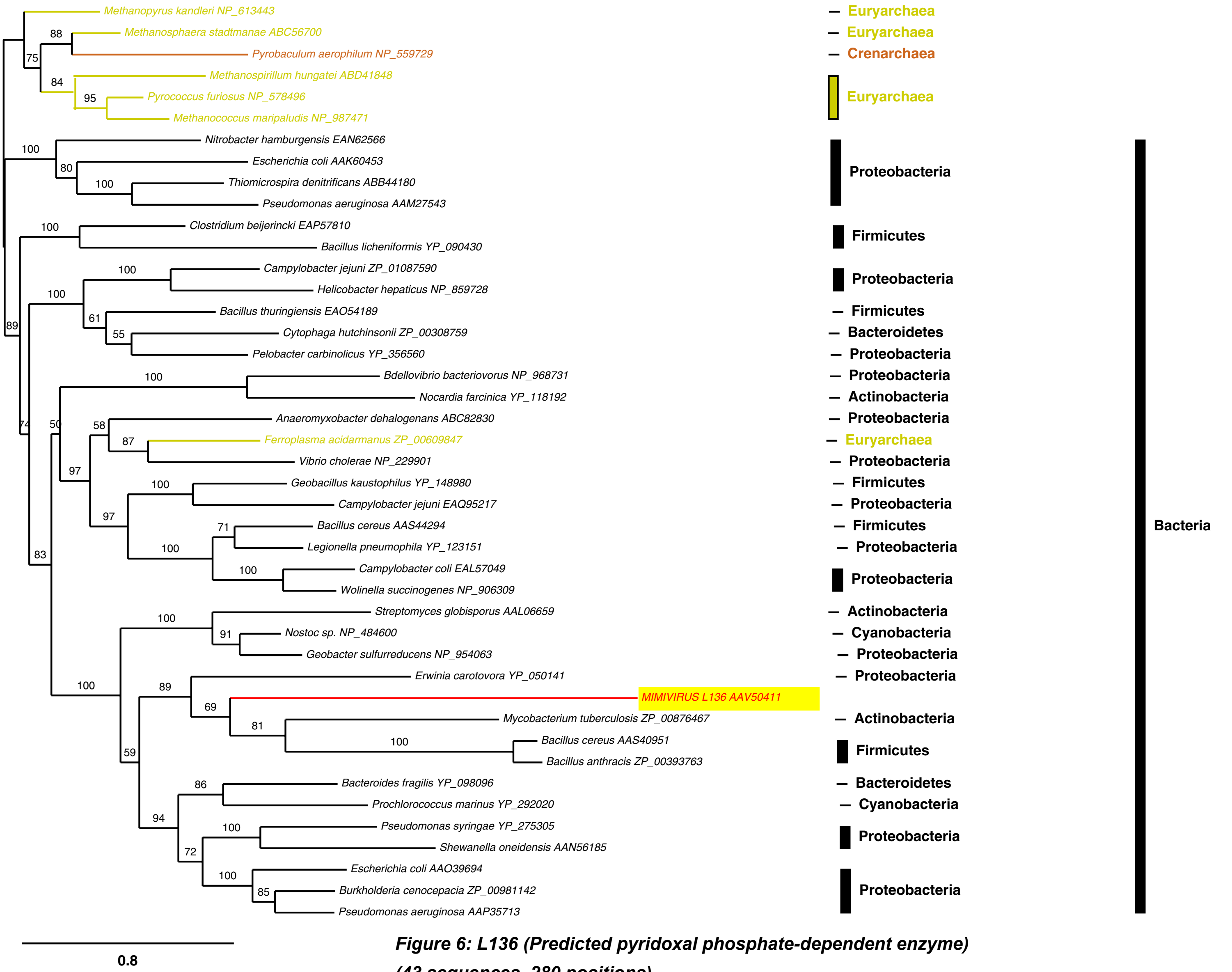

Figure 6: L136 (Predicted pyridoxal phosphate-dependent enzyme)
(43 sequences, 280 positions)

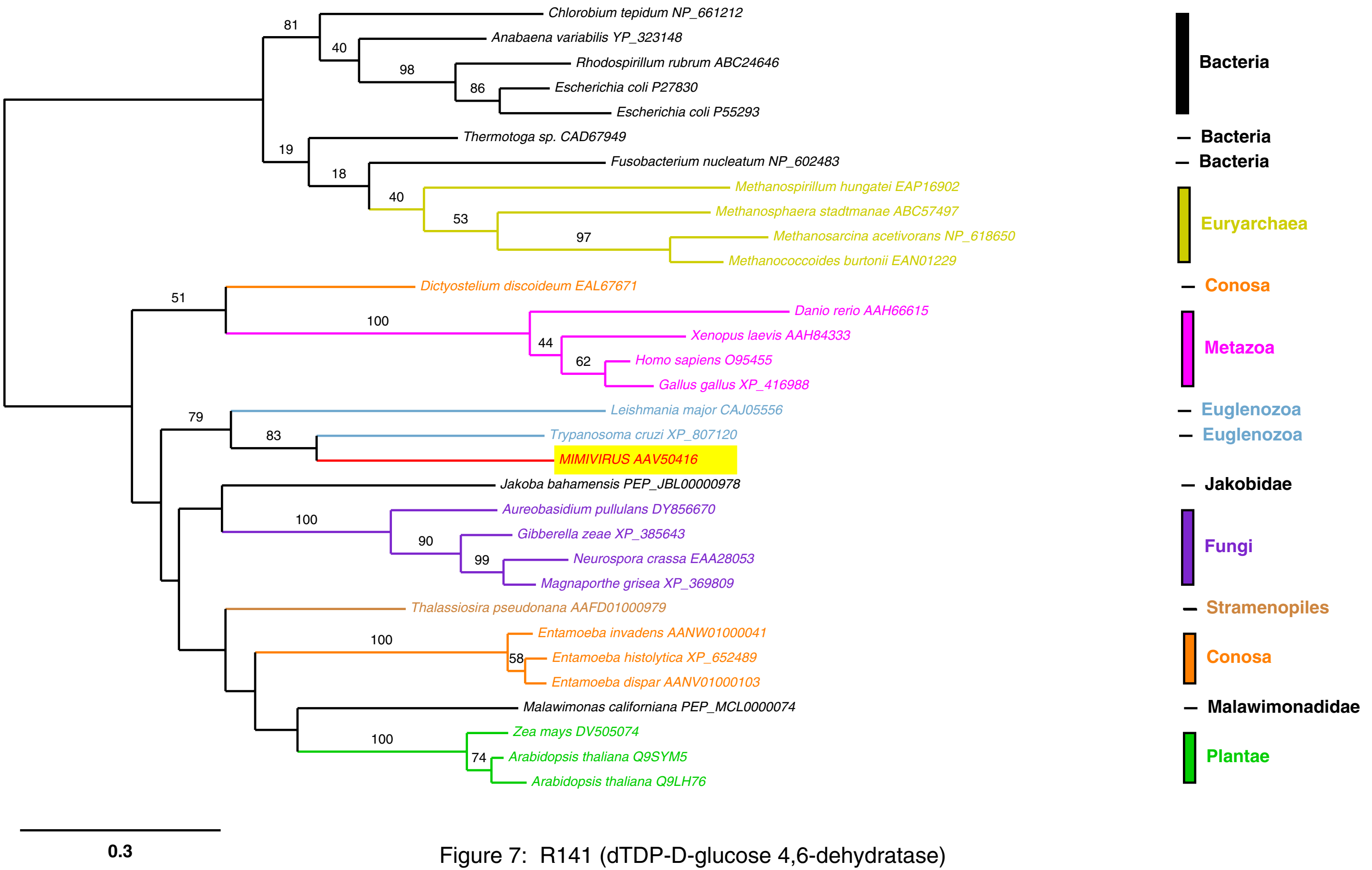

Figure 7: R141 (dTDP-D-glucose 4,6-dehydratase)
(33 sequences, 213 positions)

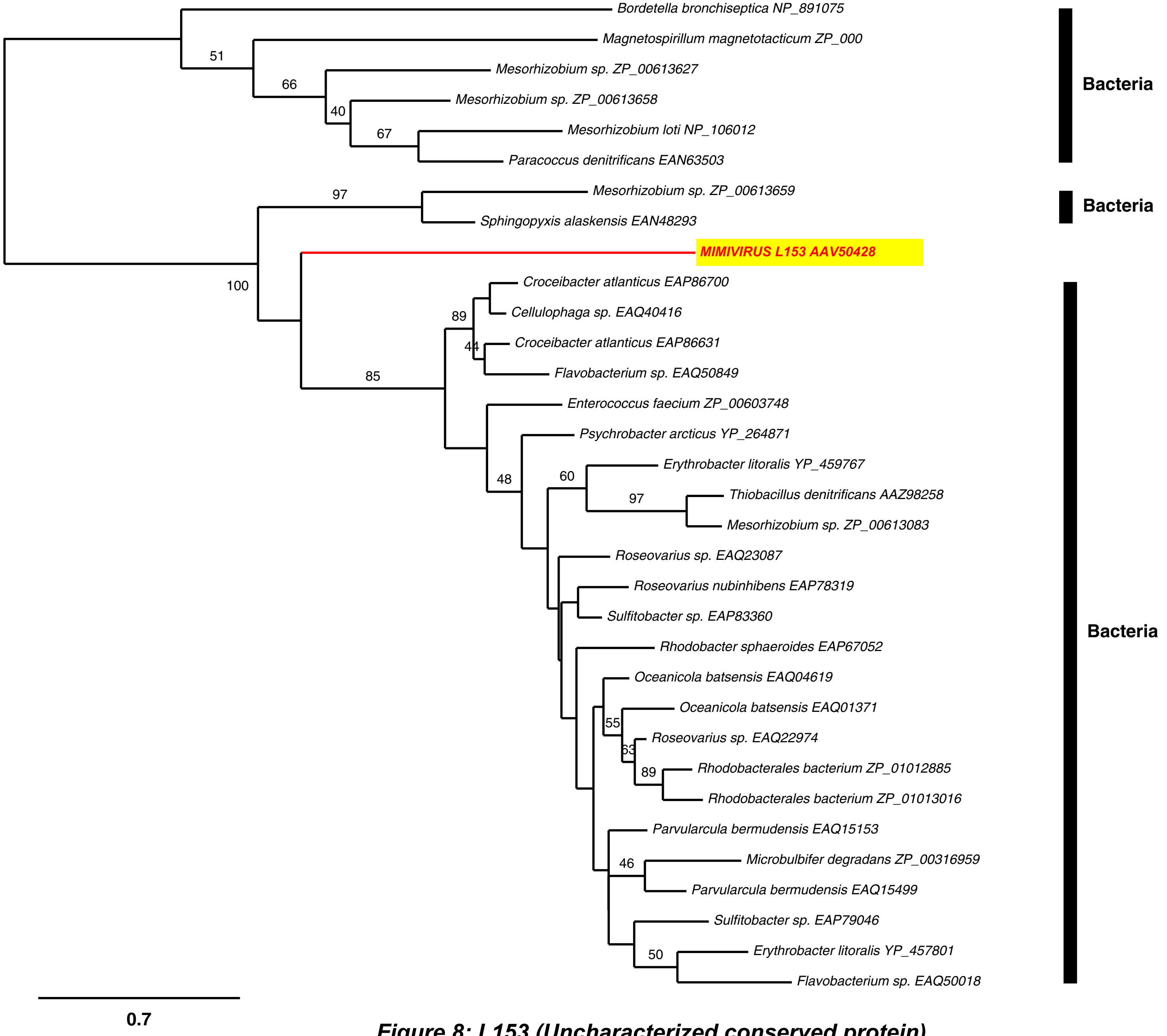

Figure 8: L153 (Uncharacterized conserved protein)

(33 sequences, 111 positions)

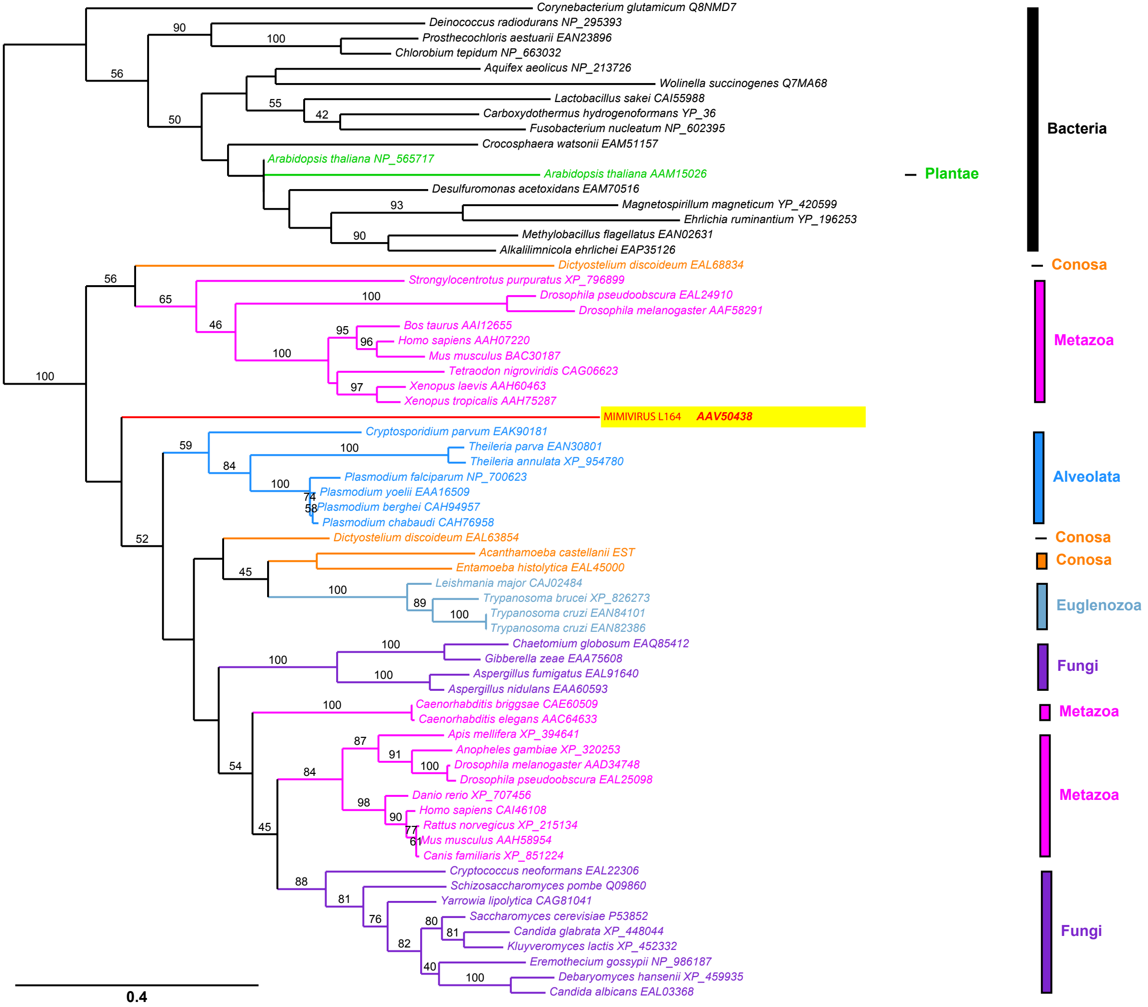

Figure 9:
L164 (Cystenyl-tRNA synthetase)
(66 sequences, 207 positions)

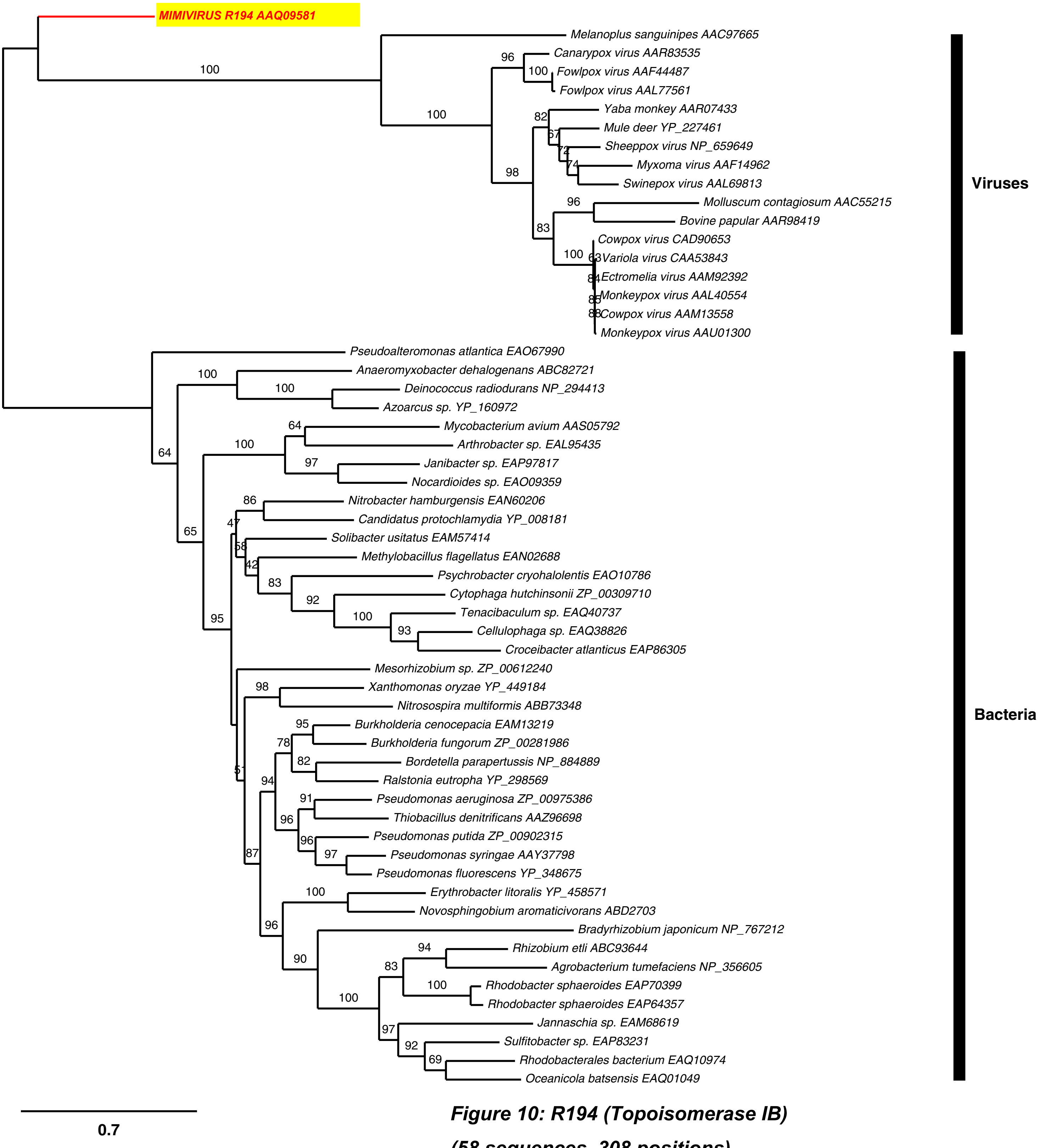

Figure 10: R194 (Topoisomerase IB)

(58 sequences, 308 positions)

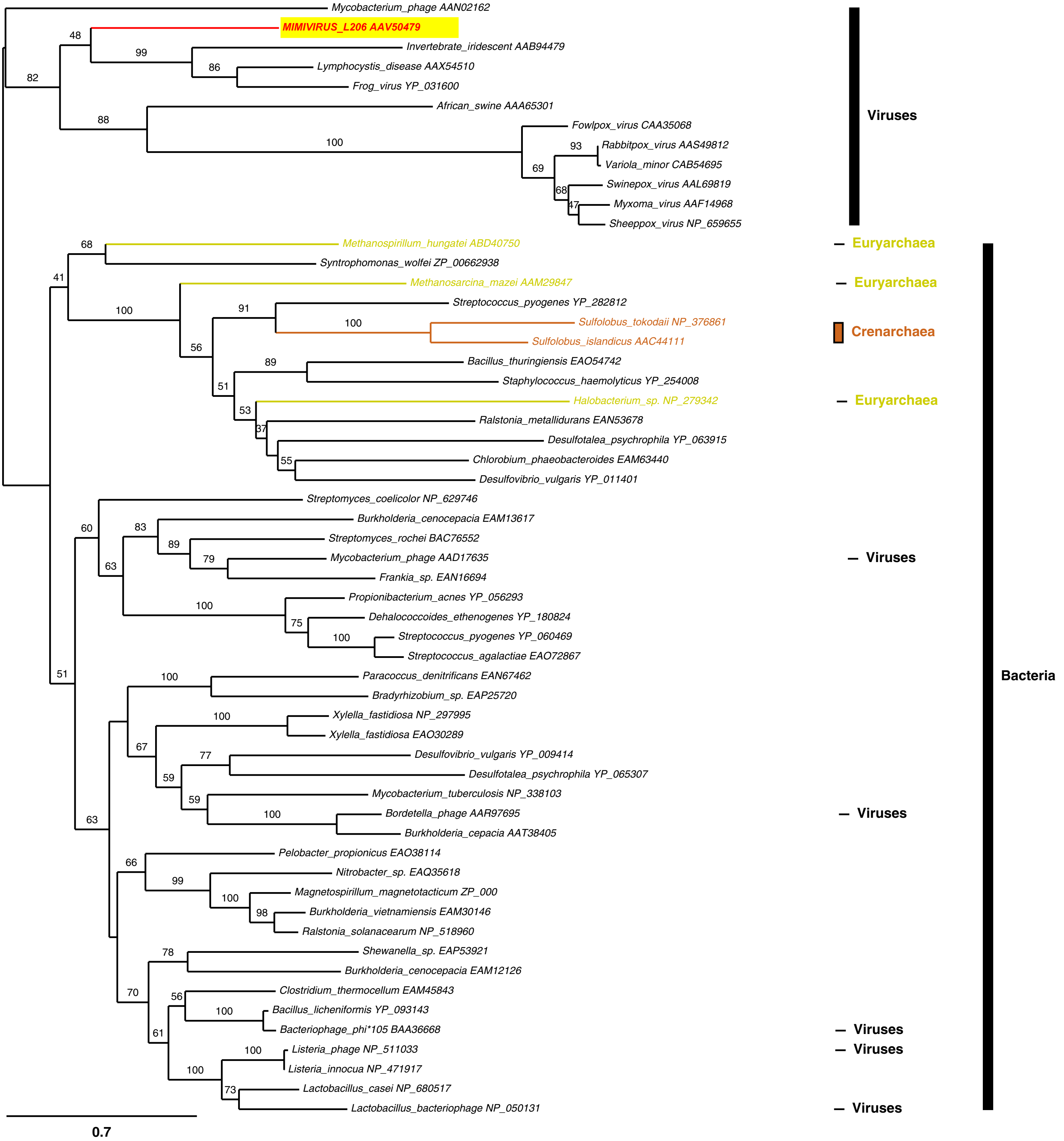

Figure 11: L206 (Predicted ATPase)

(57 sequences, 233 positions)

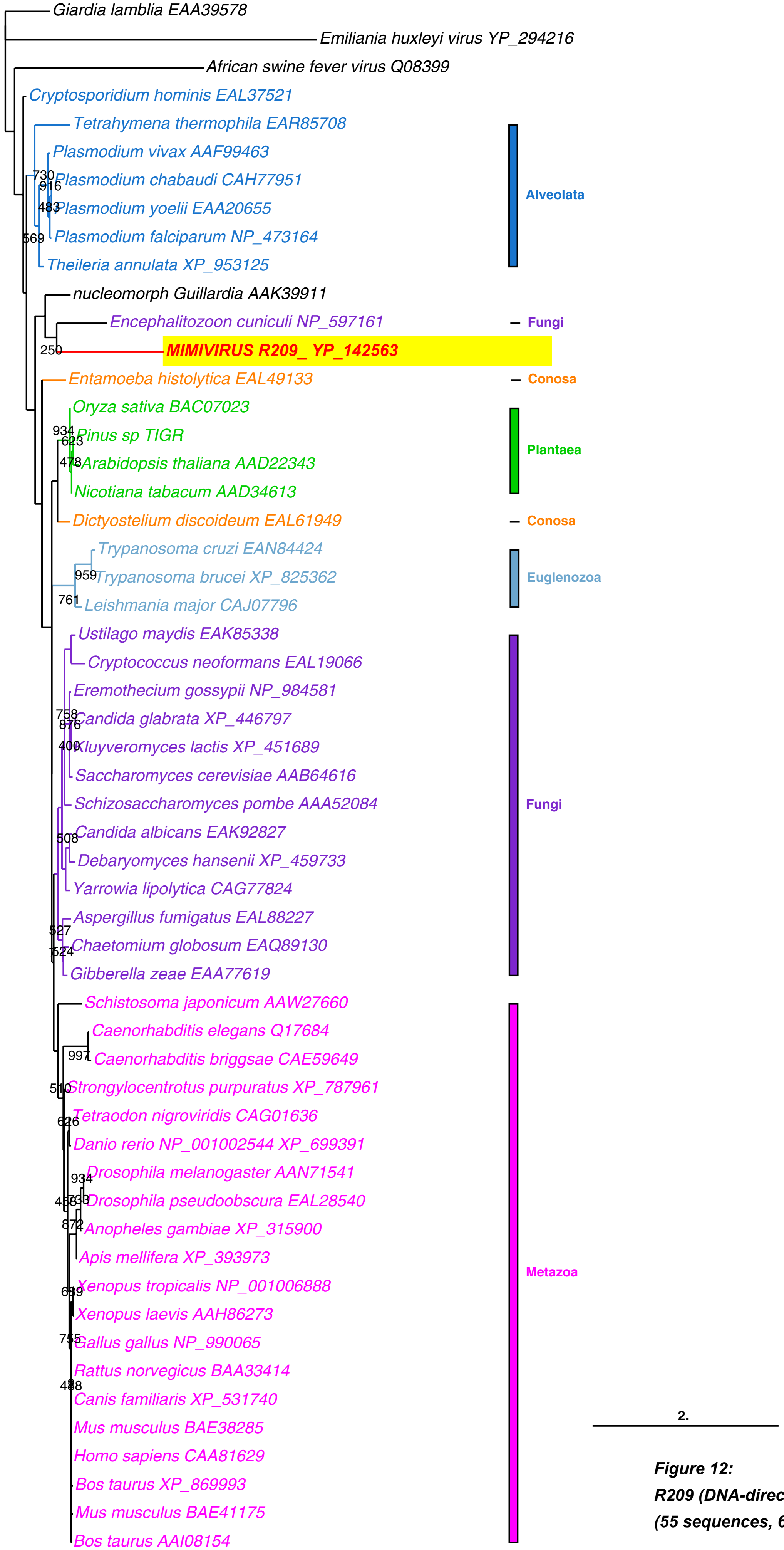

Figure 12:
R209 (DNA-directed RNA polymerase, subunit K/omega)
(55 sequences, 64 positions)

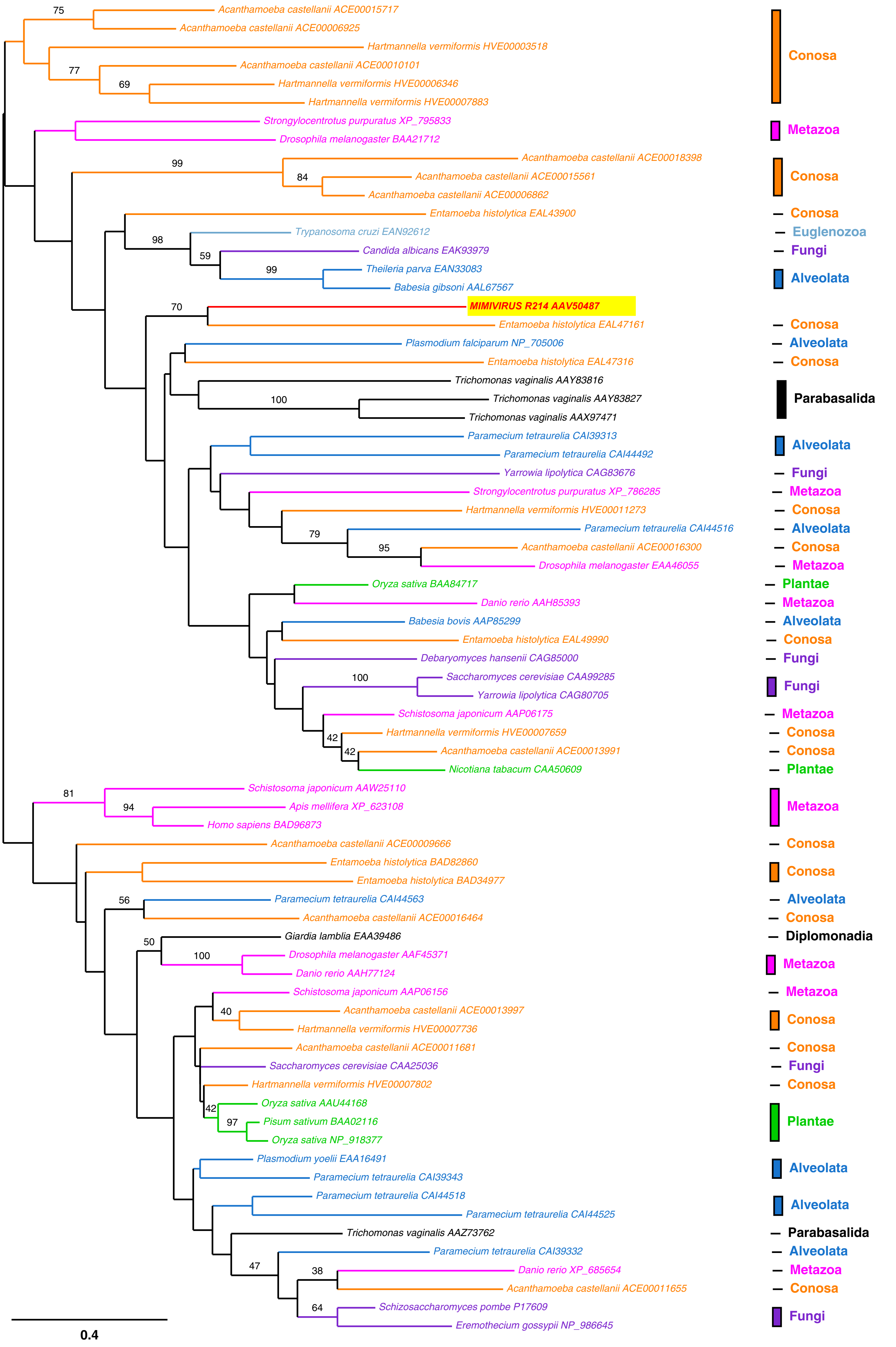

Figure 13: R214 (GTPase SAR1 and related small G proteins)
(72 sequences, 140 positions)

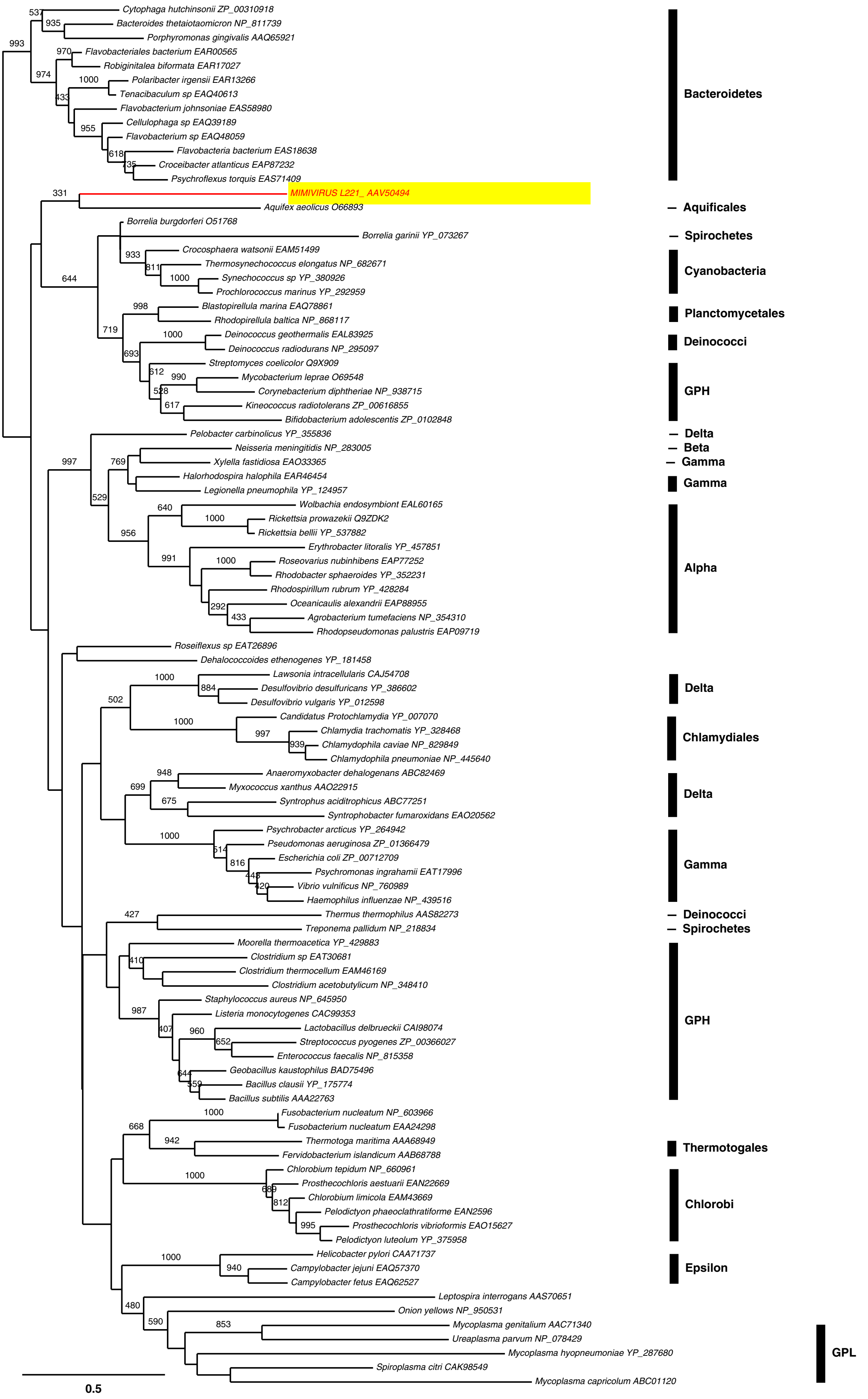

Figure 14:
L221 (Topoisomerase IA)
(98 sequences, 316 positions)

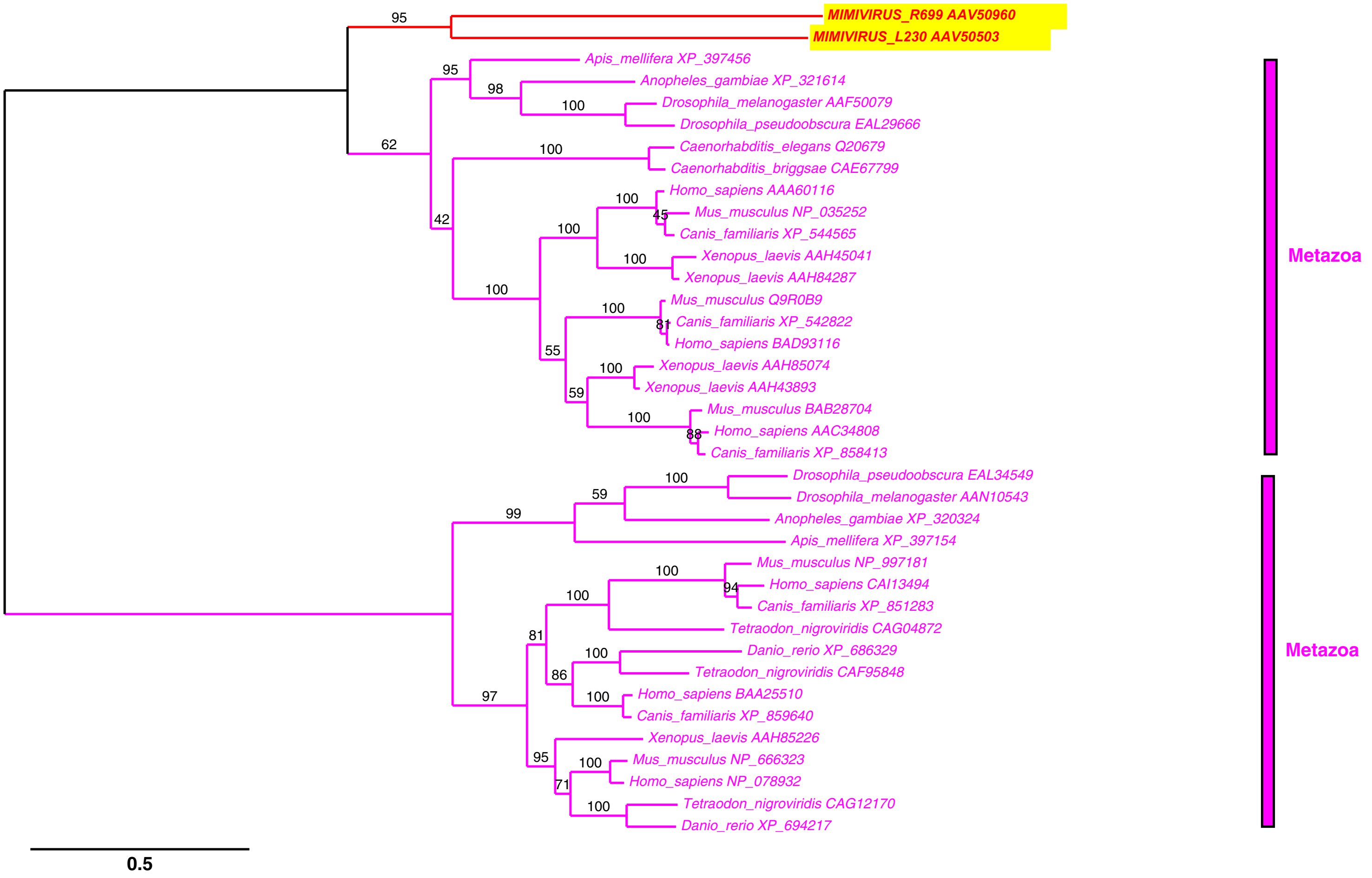

Figure 15: L230/R699 (Glycosyltransferase involved in LPS biosynthesis)
(38 sequences, 157 positions)

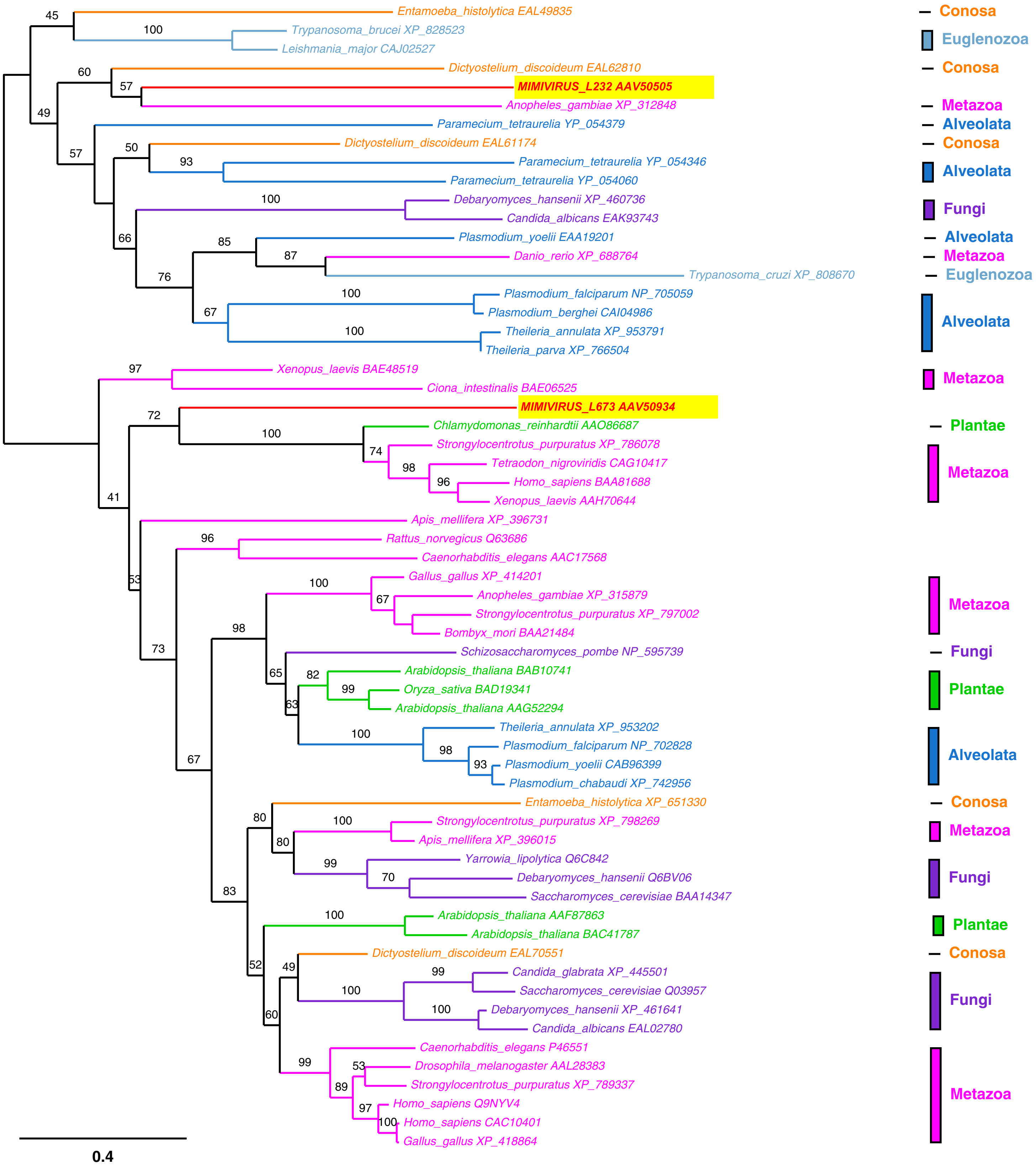

Figure 16: L232/L673 (Serine / threonine protein kinase)

(61 sequences, 166 positions)

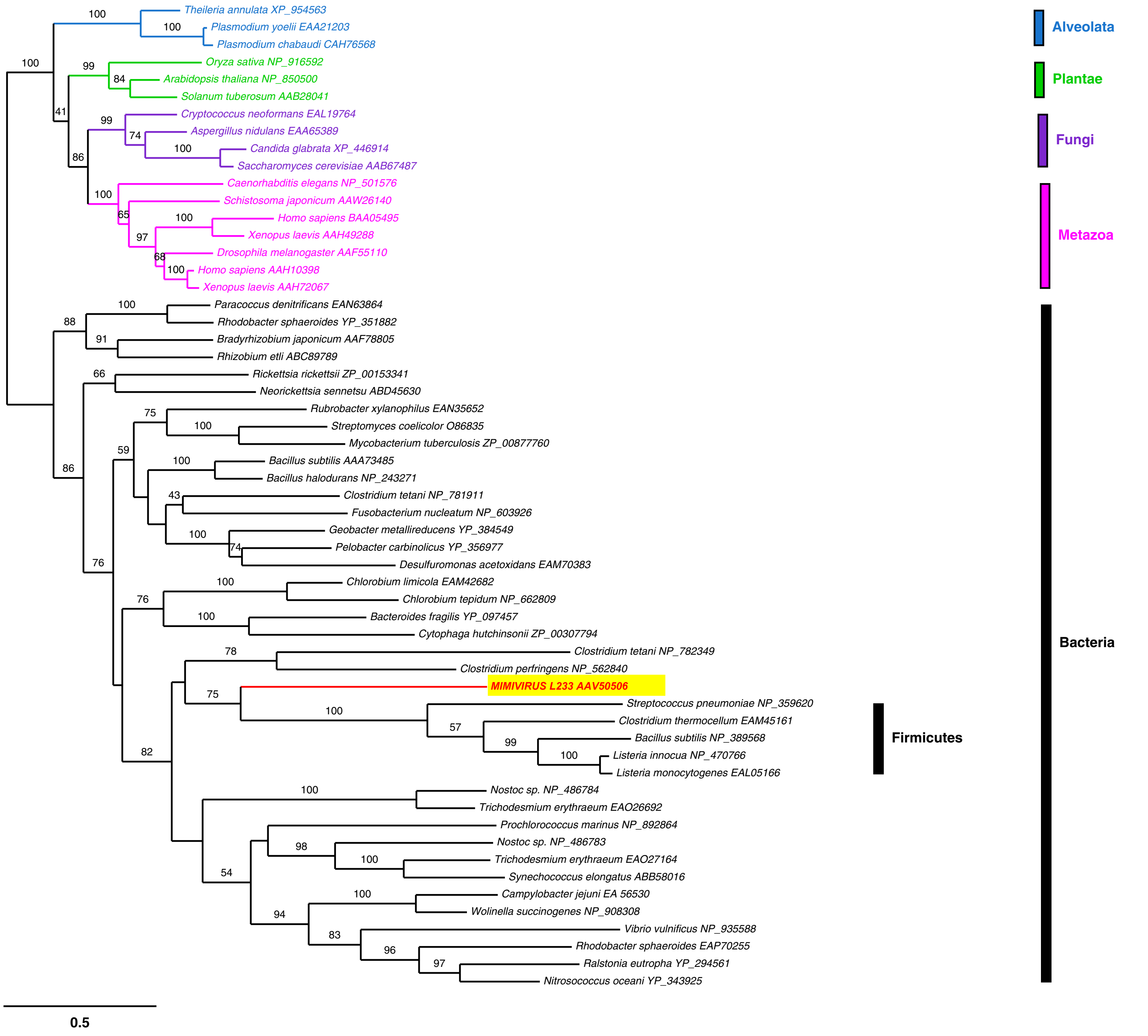

Figure 17: L233 (Predicted Zn-dependent peptidase)

(57 sequences, 296 positions)

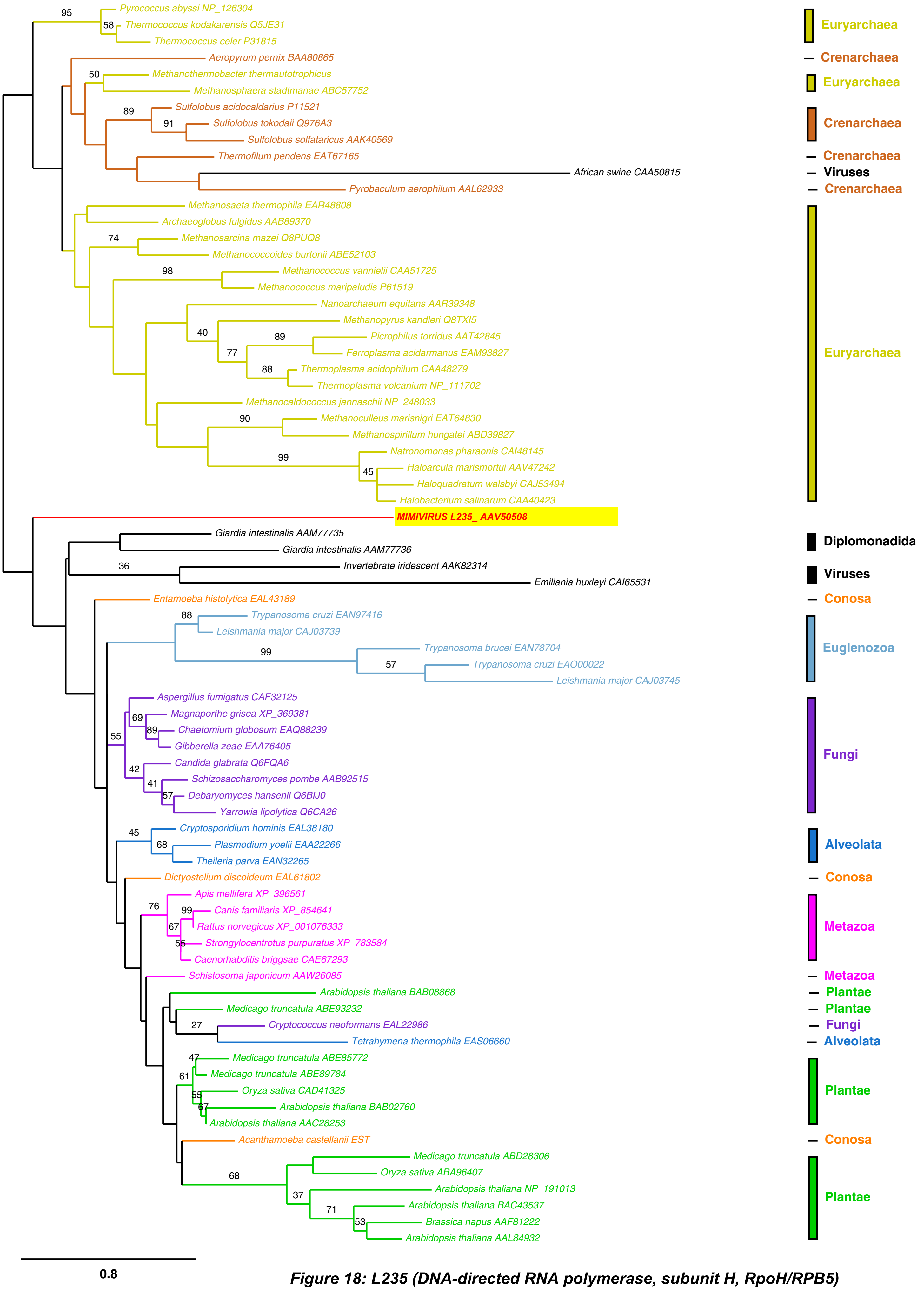

Figure 18: L235 (DNA-directed RNA polymerase, subunit H, RpoH/RPB5) (76 sequences, 62 positions)

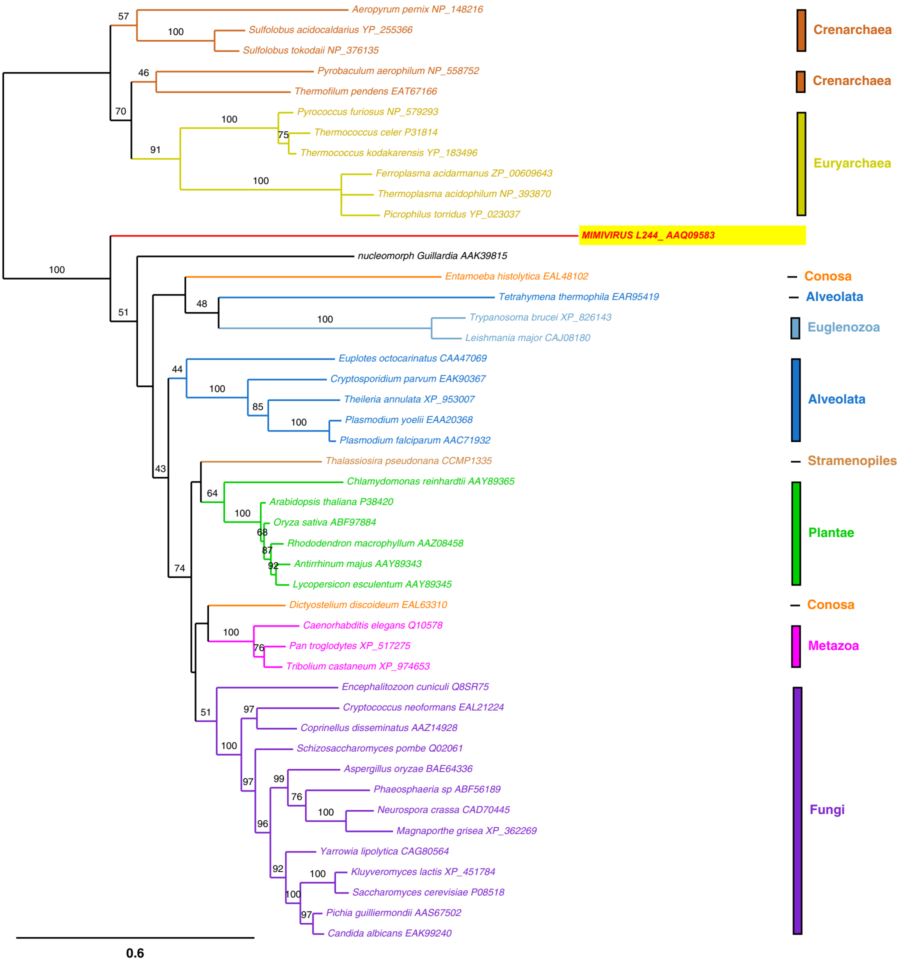

Figure 19:

L244 (DNA-directed RNA polymerase, beta subunit/140 kD subunit)

(46 sequences, 766 positions)

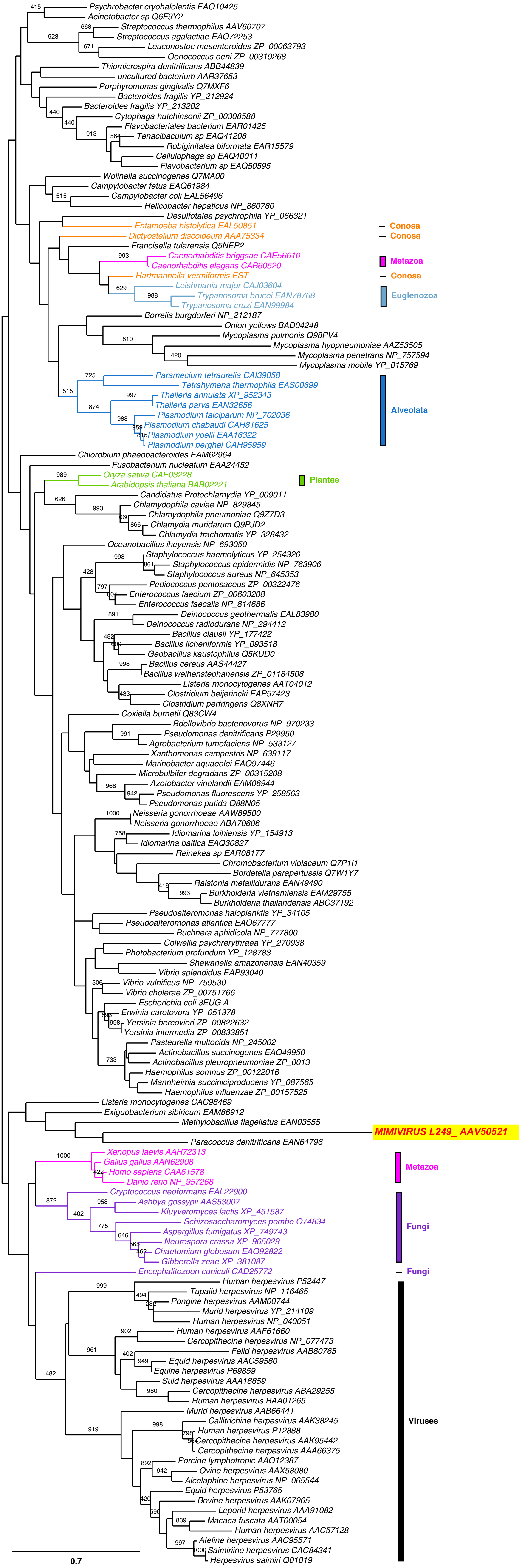

Figure 20:
L249 (Uracil DNA glycosylase)
(157 sequences, 139 positions)

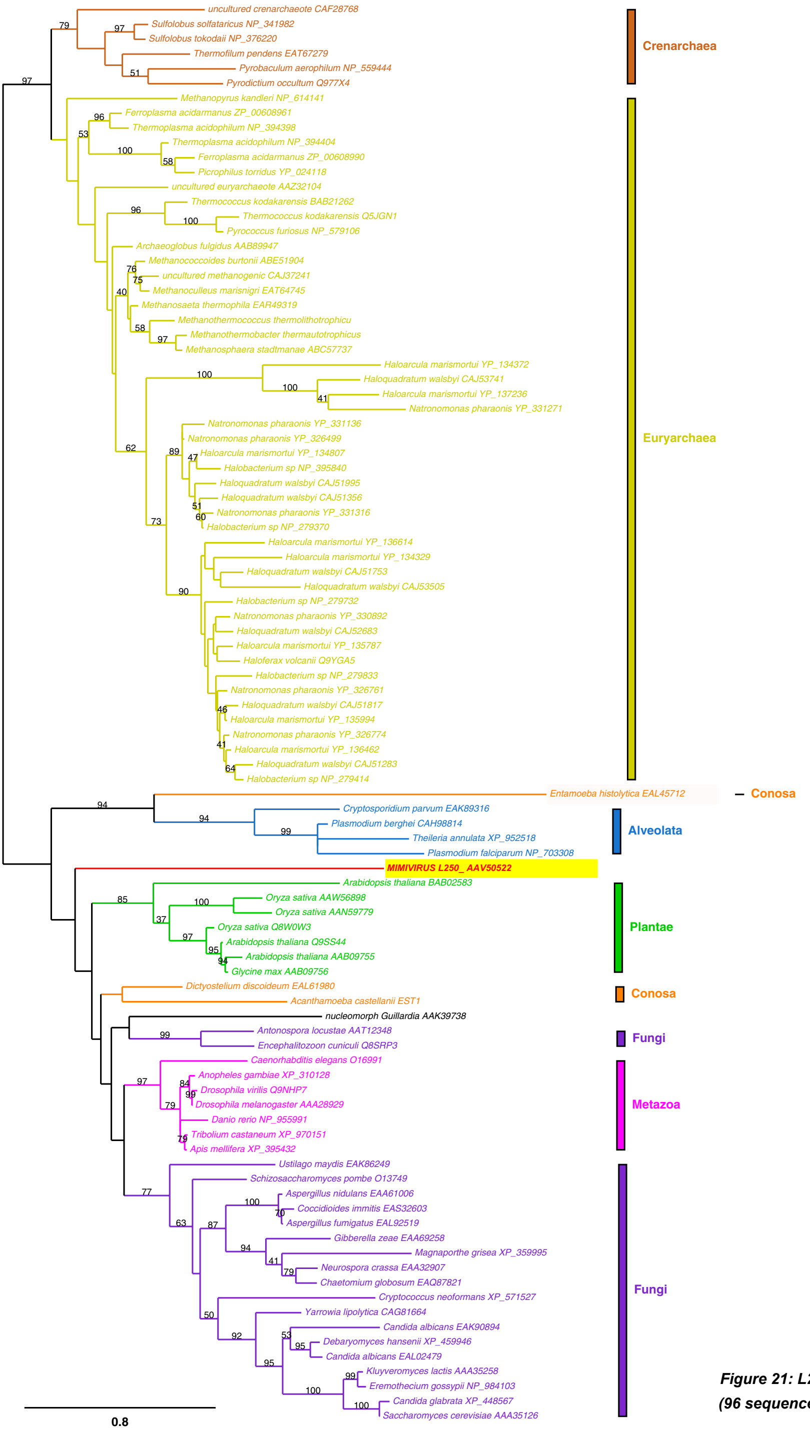

Figure 21: L250 (Transcription initiation factor TFIIIB, Brf1 subunit/Transcription initiation factor TFIIB) (96 sequences, 152 positions)

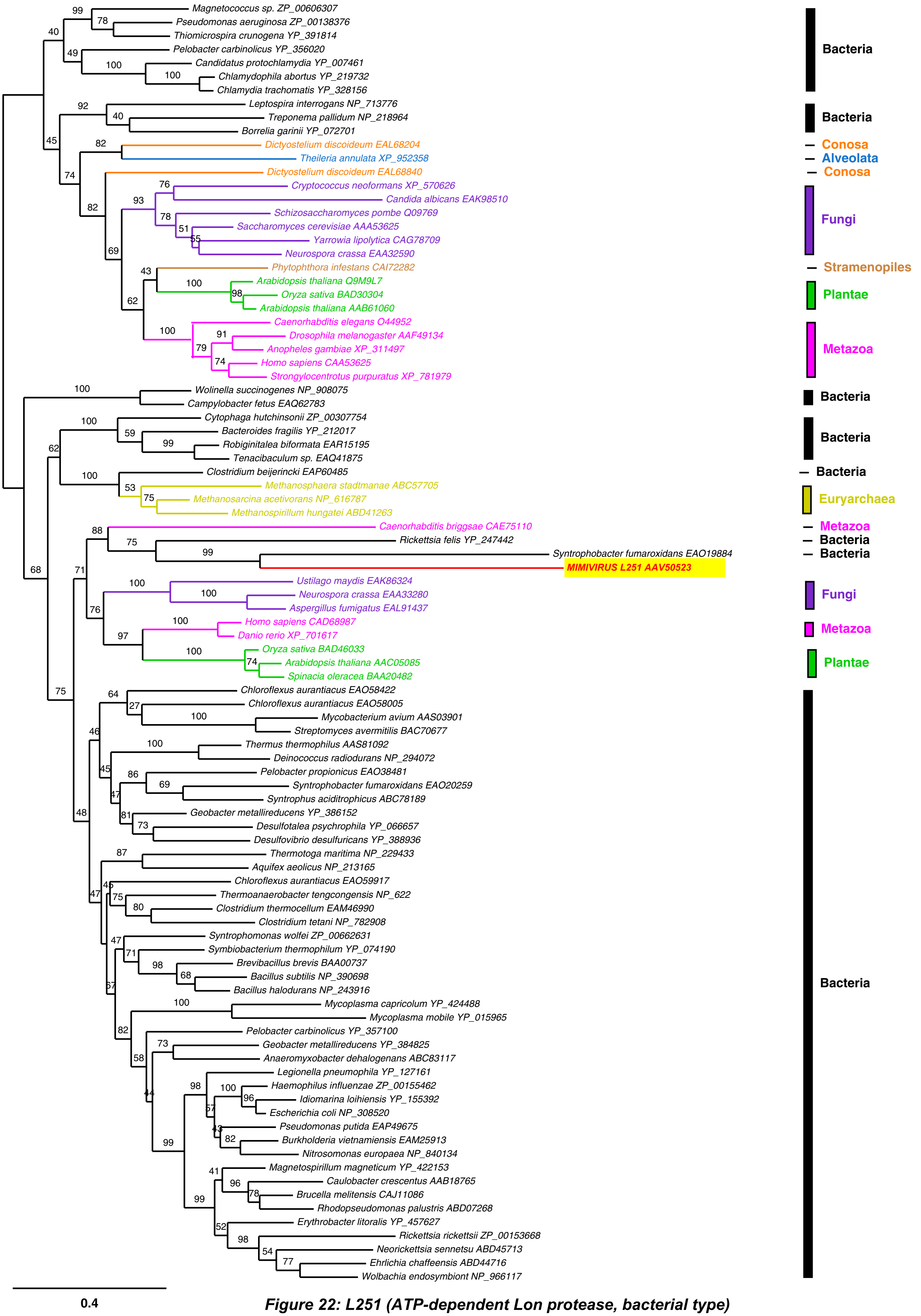

Figure 22: L251 (ATP-dependent Lon protease, bacterial type)

(94 sequences, 306 positions)

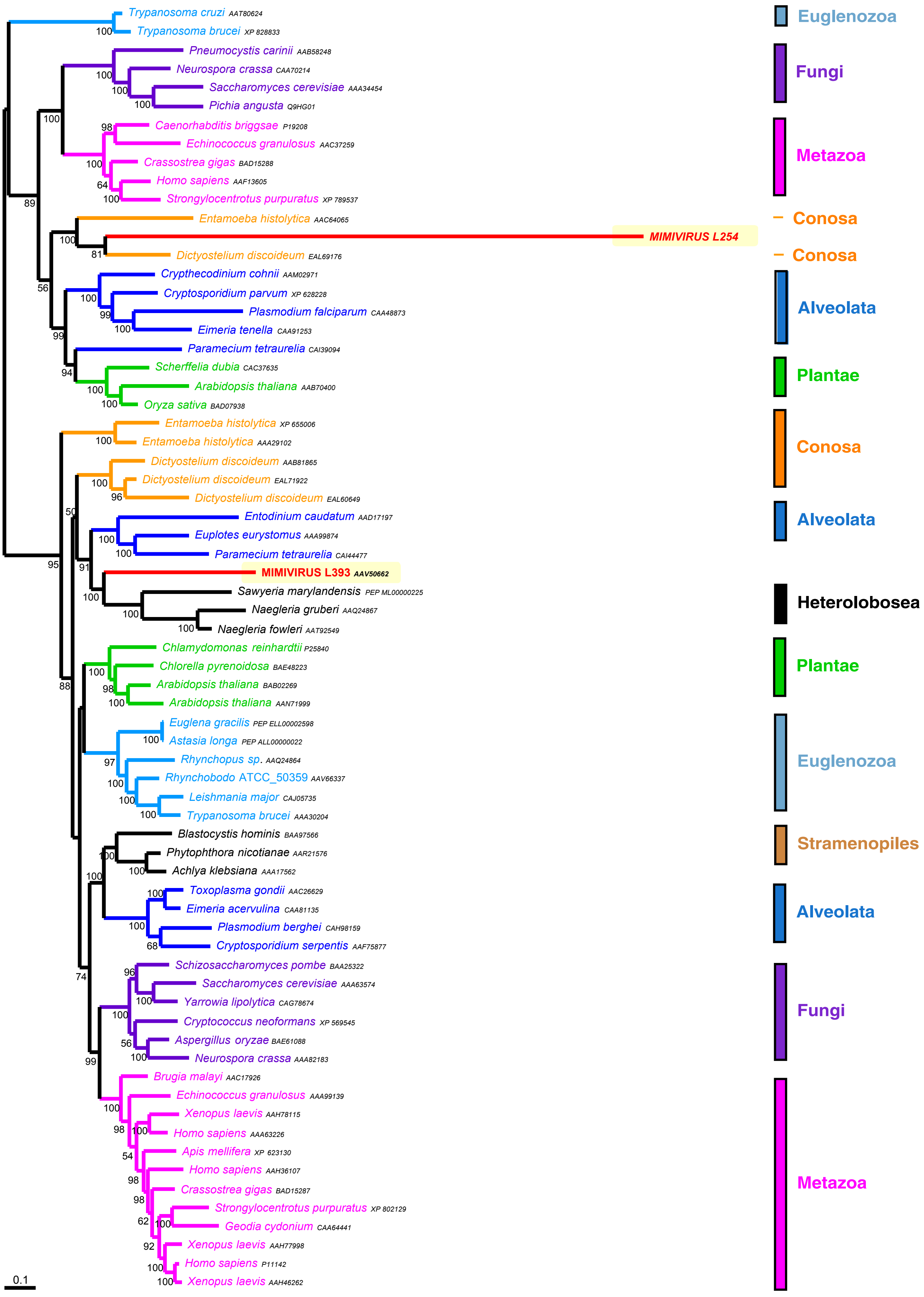

Figure 23: L254/L393 (Molecular chaperone)

(69 sequences, 570 positions)

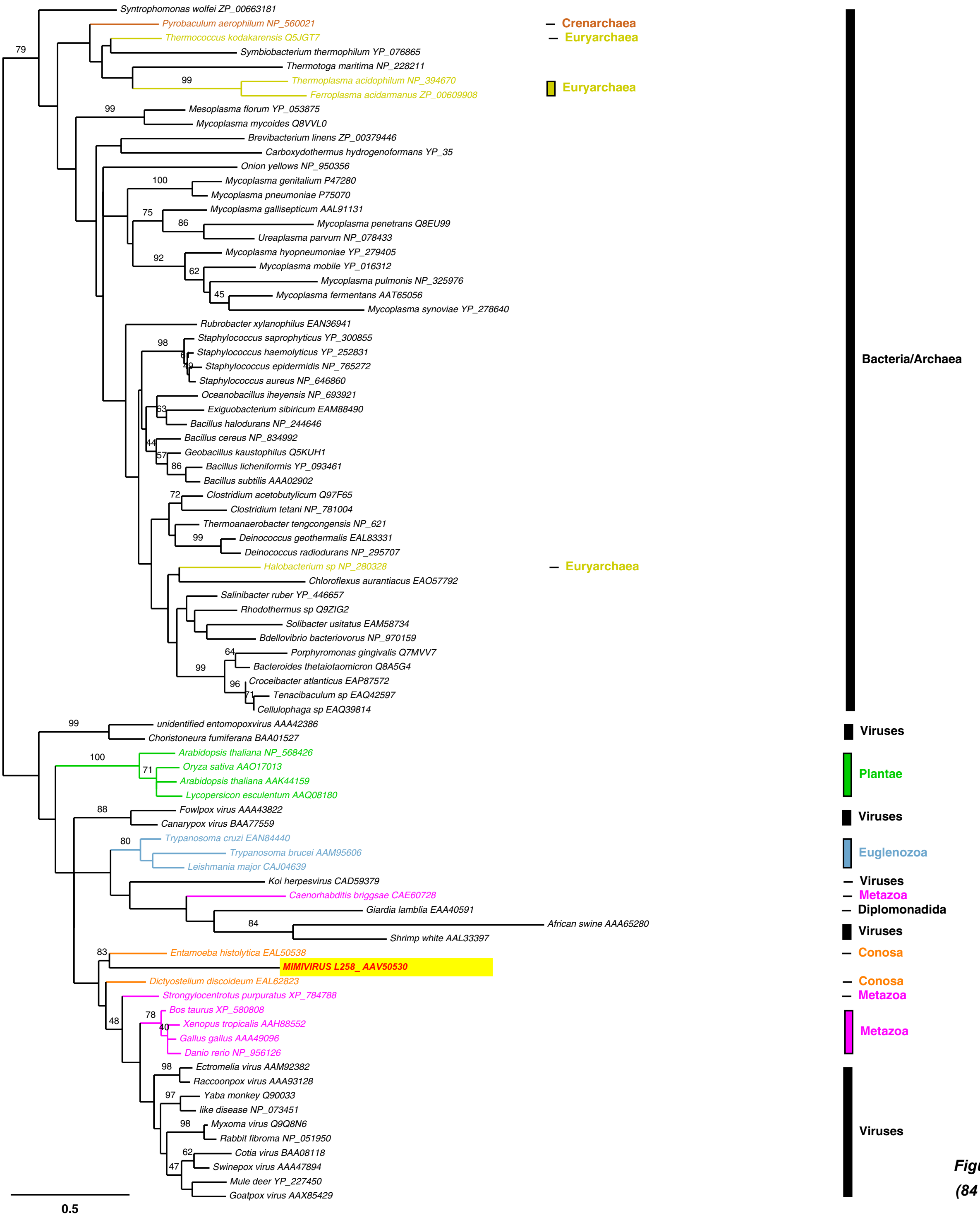

Figure 24: L258 (Thymidine kinase)
(84 sequences, 106 positions)

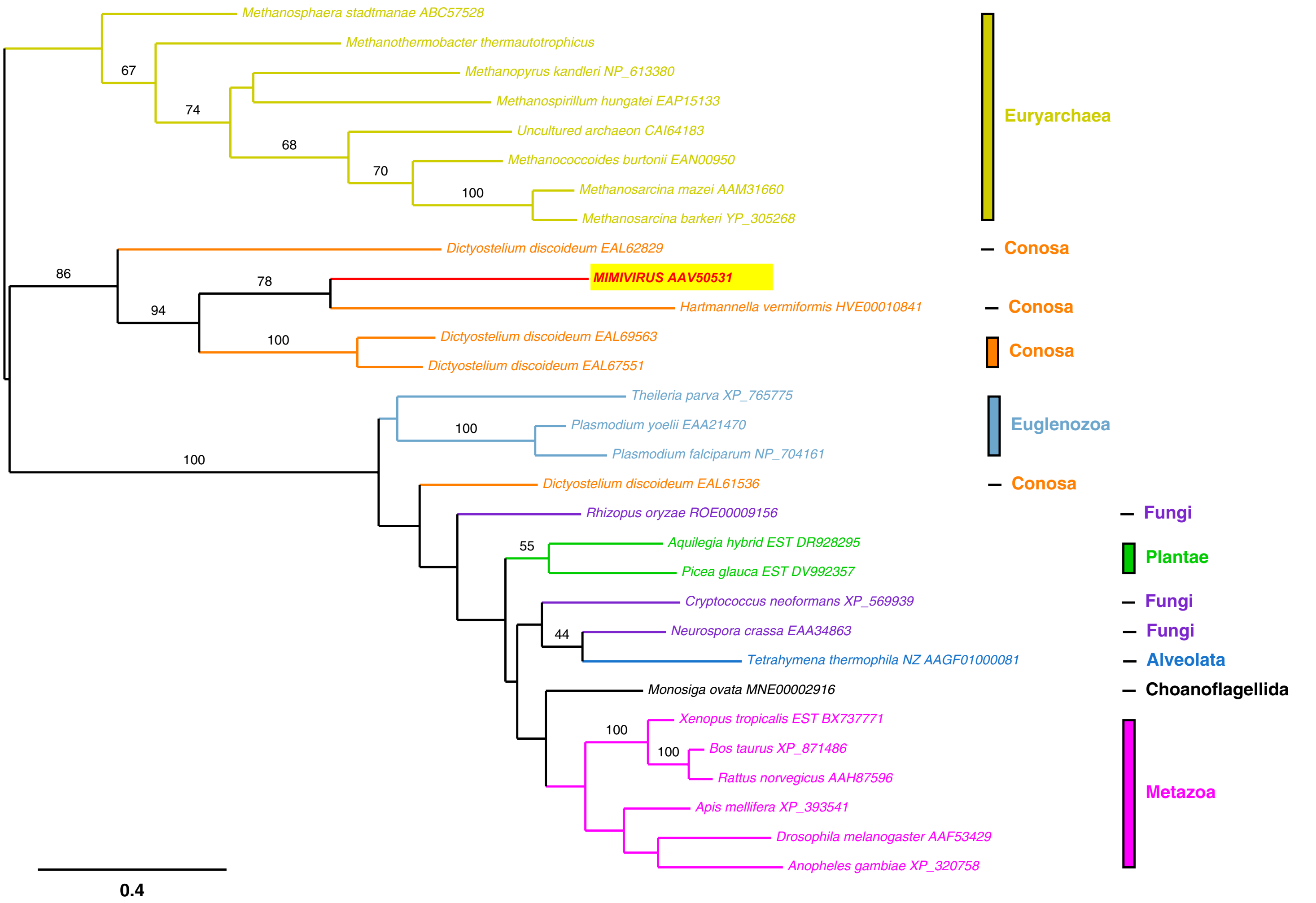

Figure 25: R259 (Uncharacterized conserved protein)
(30 sequences, 126 positions)

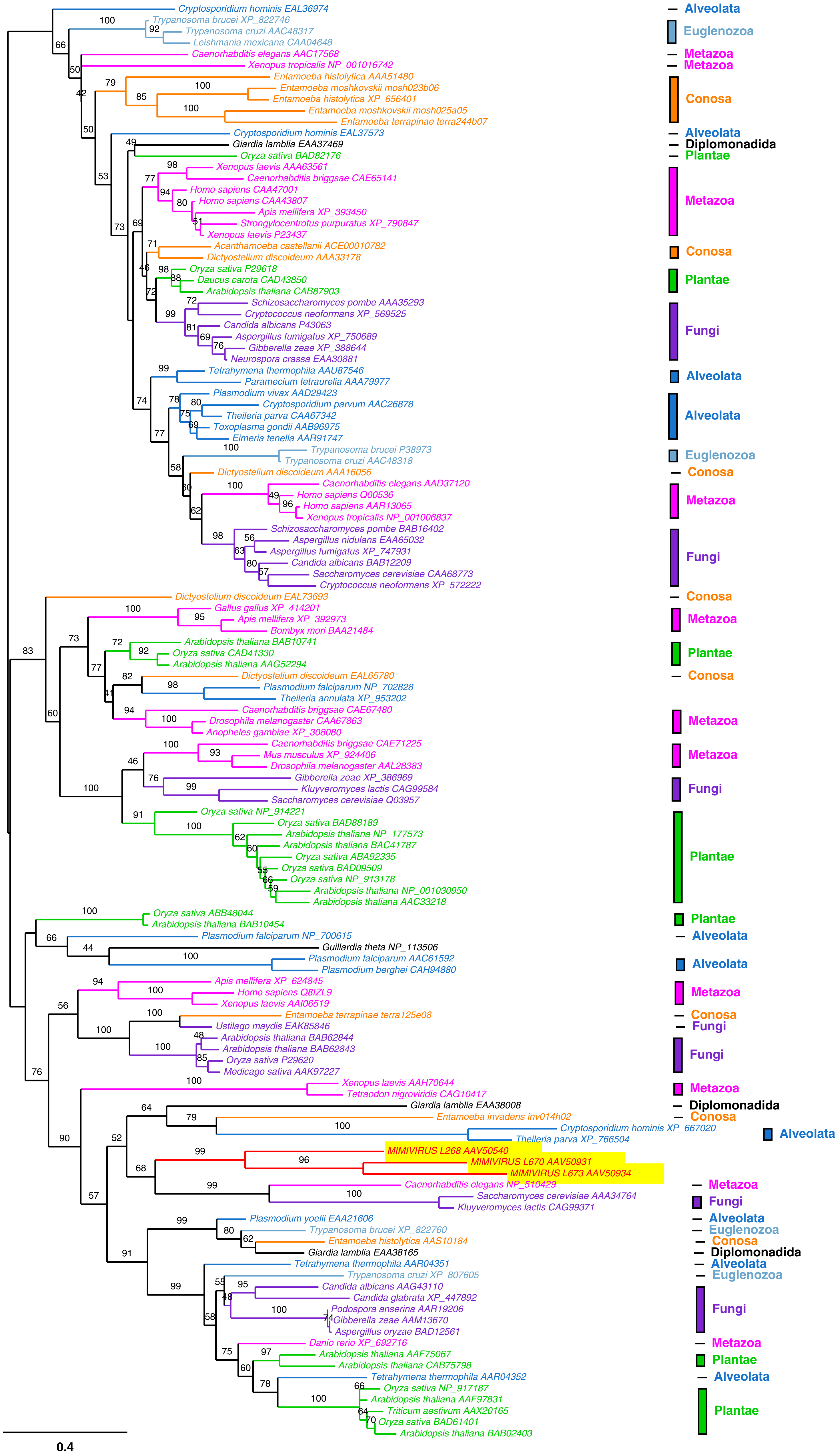

Figure 26: L268/L670/L673 (Serine / threonine protein kinase)
(127 sequences, 150 positions)

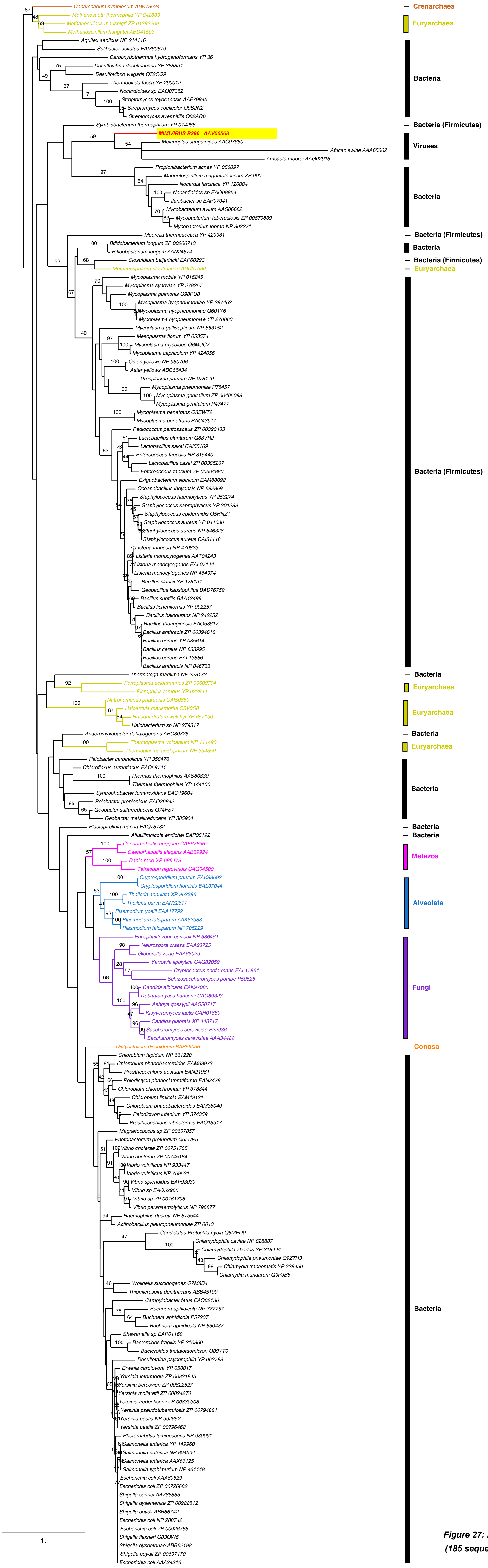

Figure 27: R296 (Endonuclease IV)
(185 sequences, 102 positions)

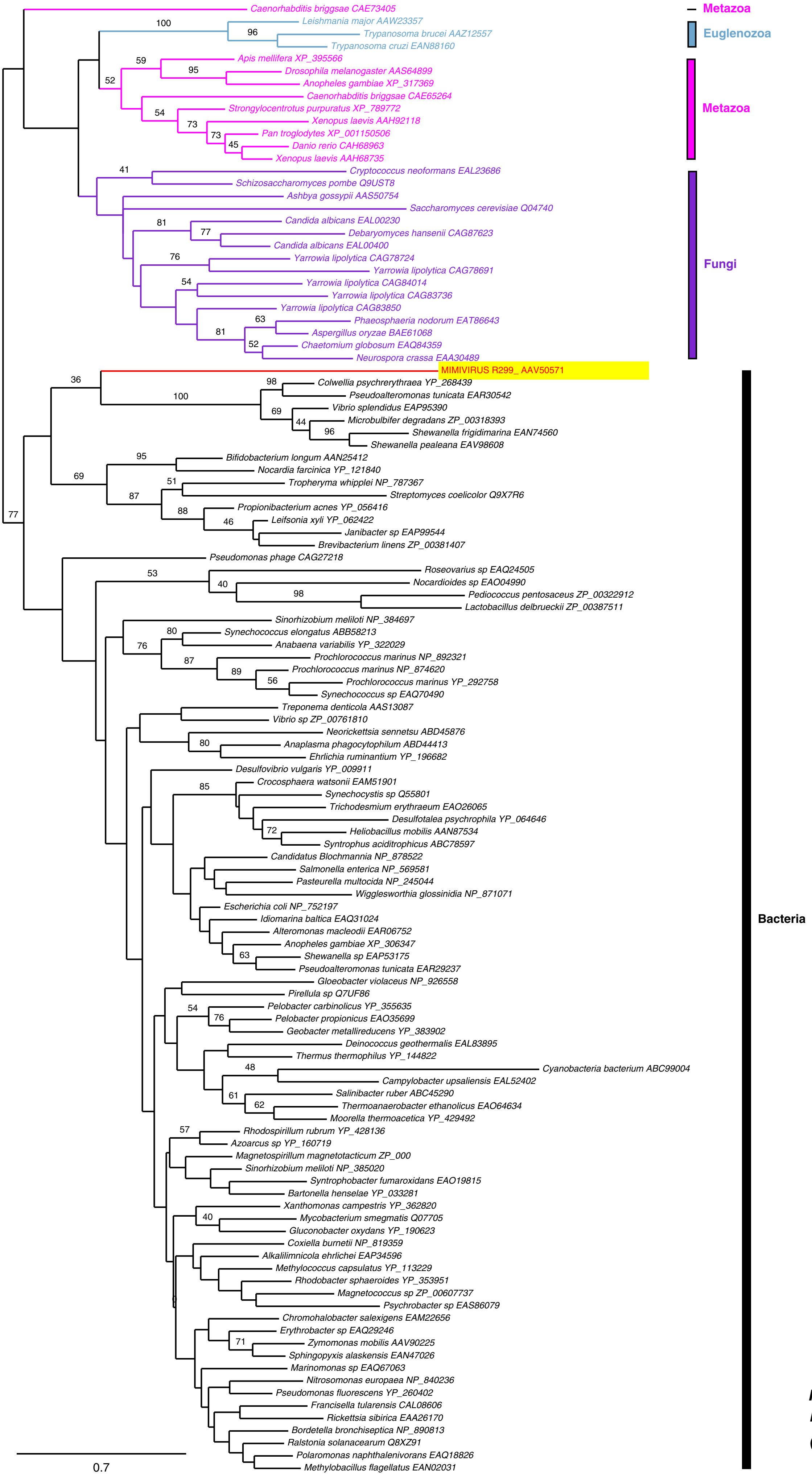

Figure 28:
R299 (Ribonucleotide reductase HI)
(118 sequences, 106 positions)

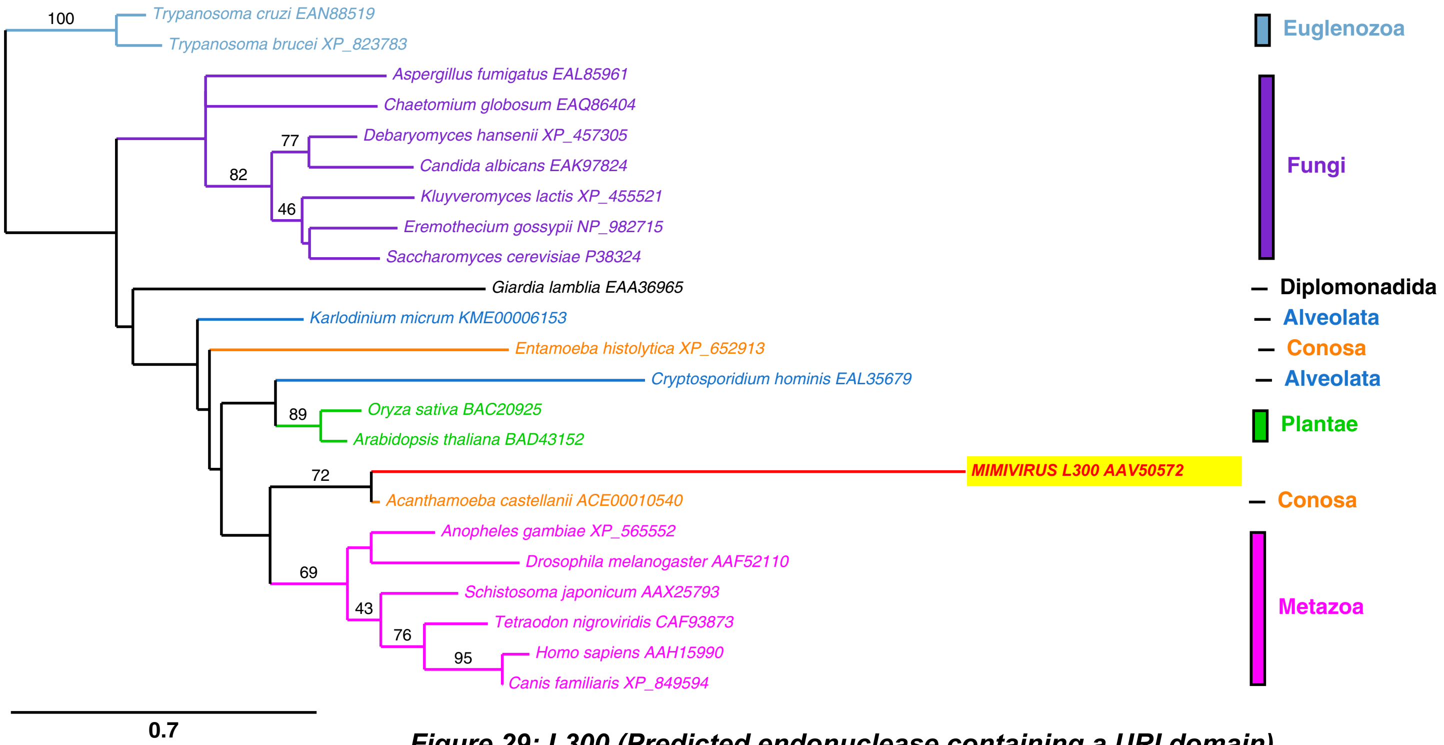

Figure 29: L300 (Predicted endonuclease containing a URI domain) (28 sequences, 67 positions)

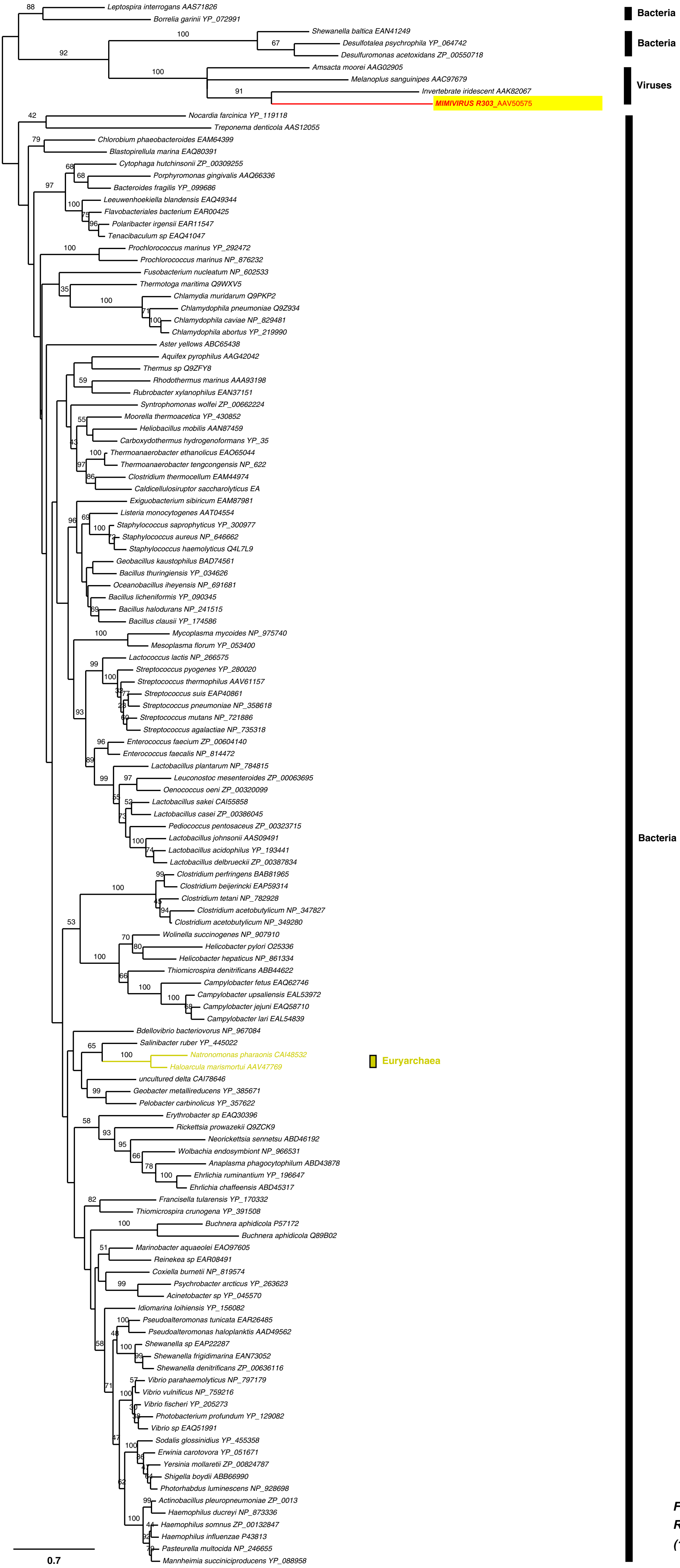

Figure 30:
R303 (NAD-dependent DNA ligase)
(130 sequences, 297 positions)

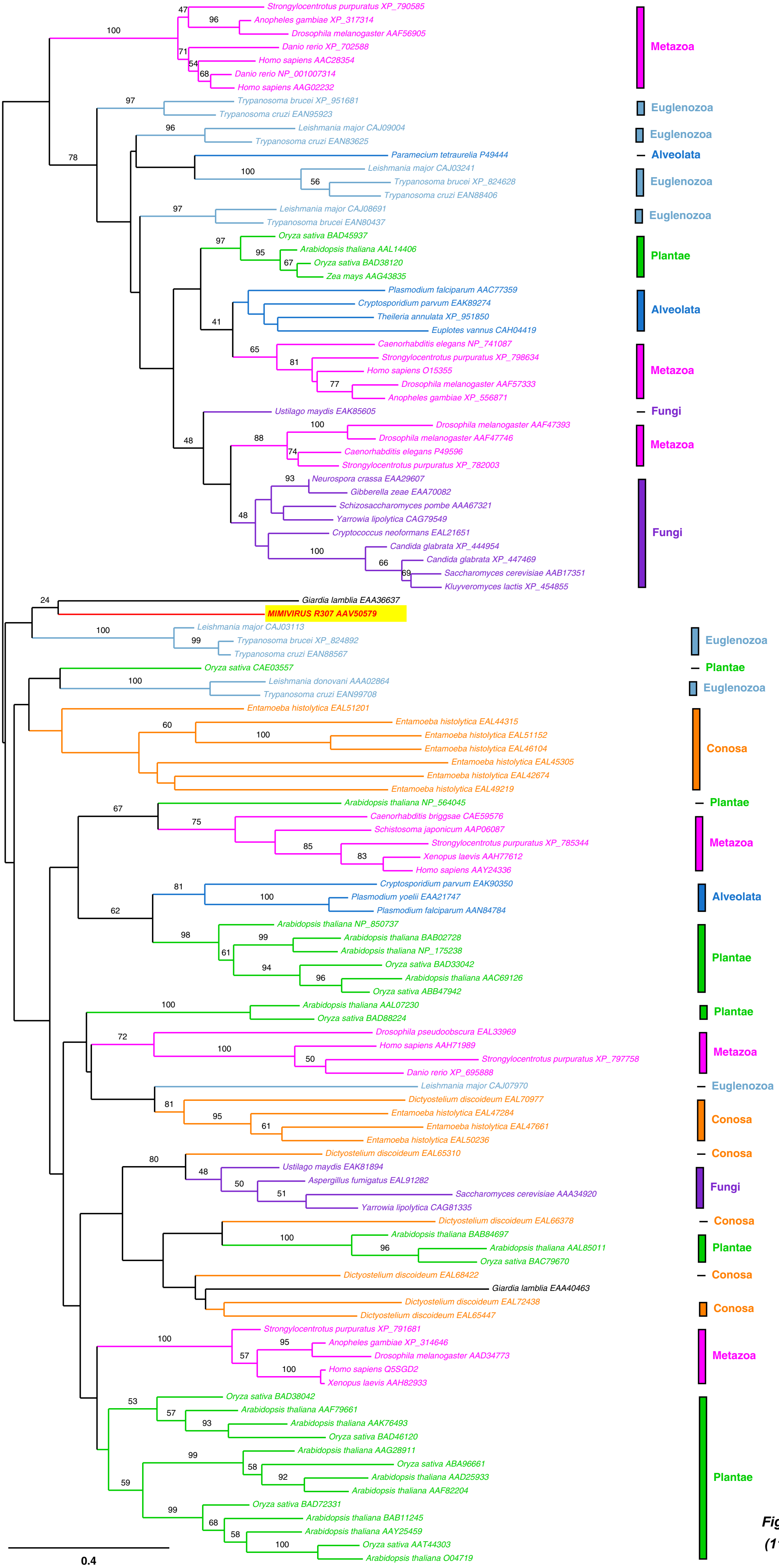

Figure 31:R307 (Serine/threonine protein phosphatase)
(116 sequences, 119 positions)

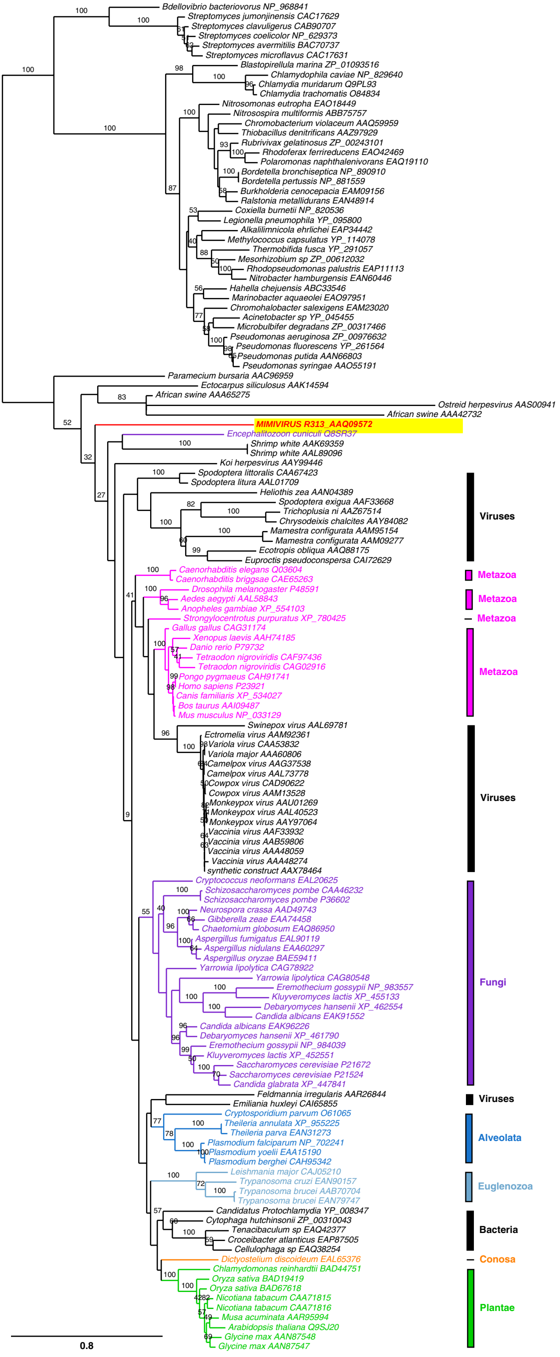

FIGURE 32: R313 (Ribonucleotide reductase, alpha subunit)
(139 sequences, 500 positions)

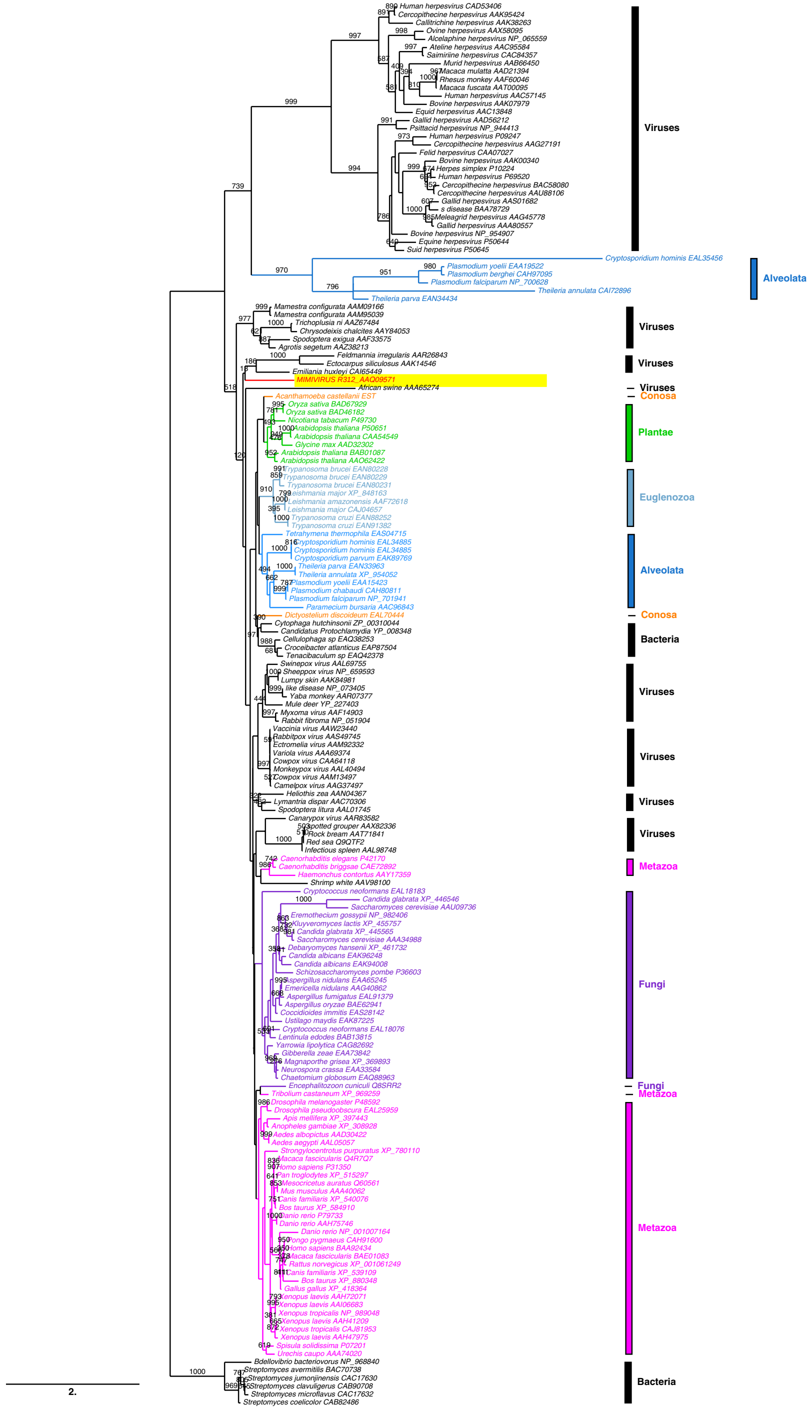

Figure 33: L312 (Ribonucleotide reductase, beta subunit)
(173 sequences, 183 positions)

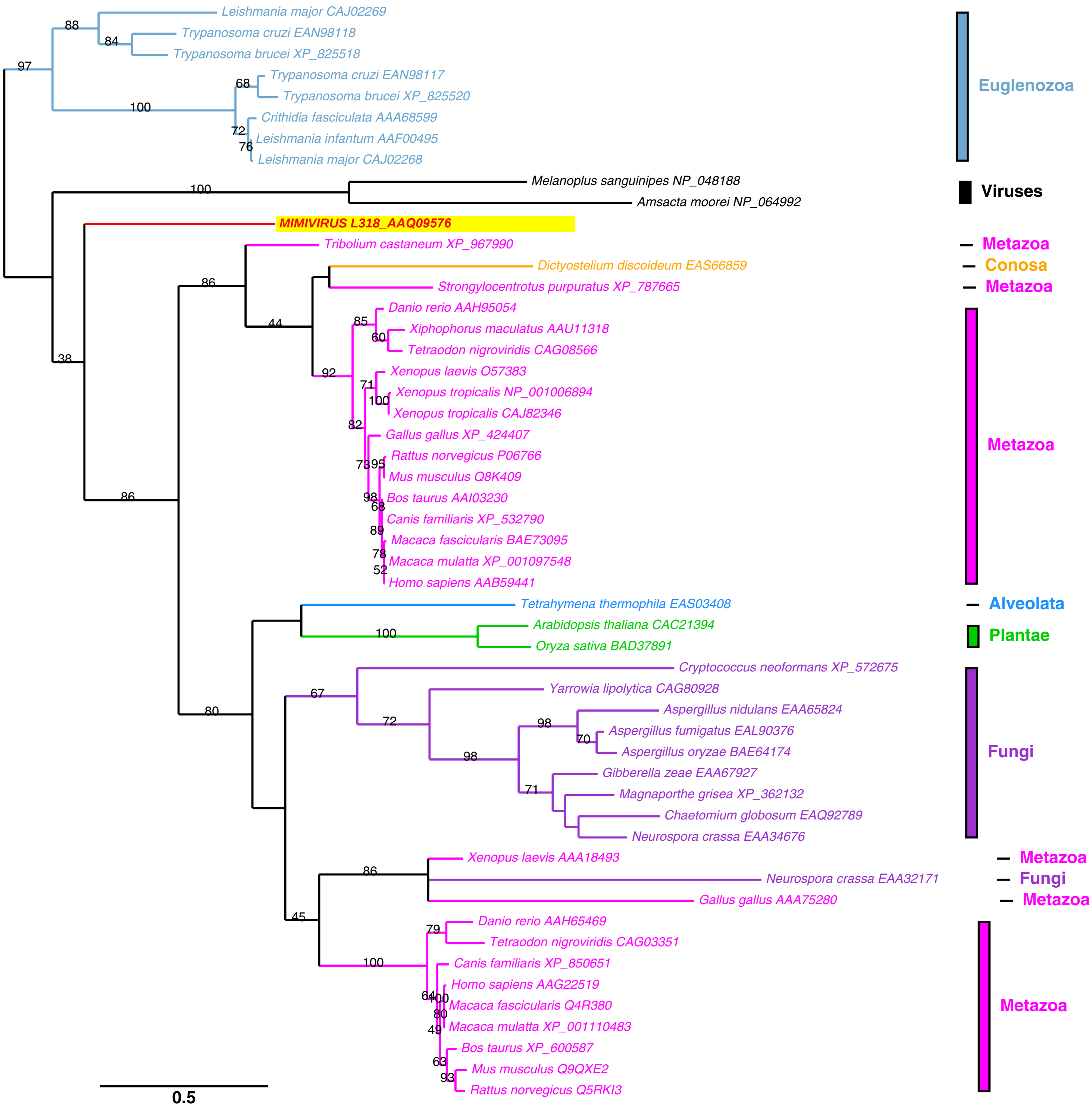

Figure 34: L318 (DNA polymerase IV - family X)
(52 sequences, 165 positions)

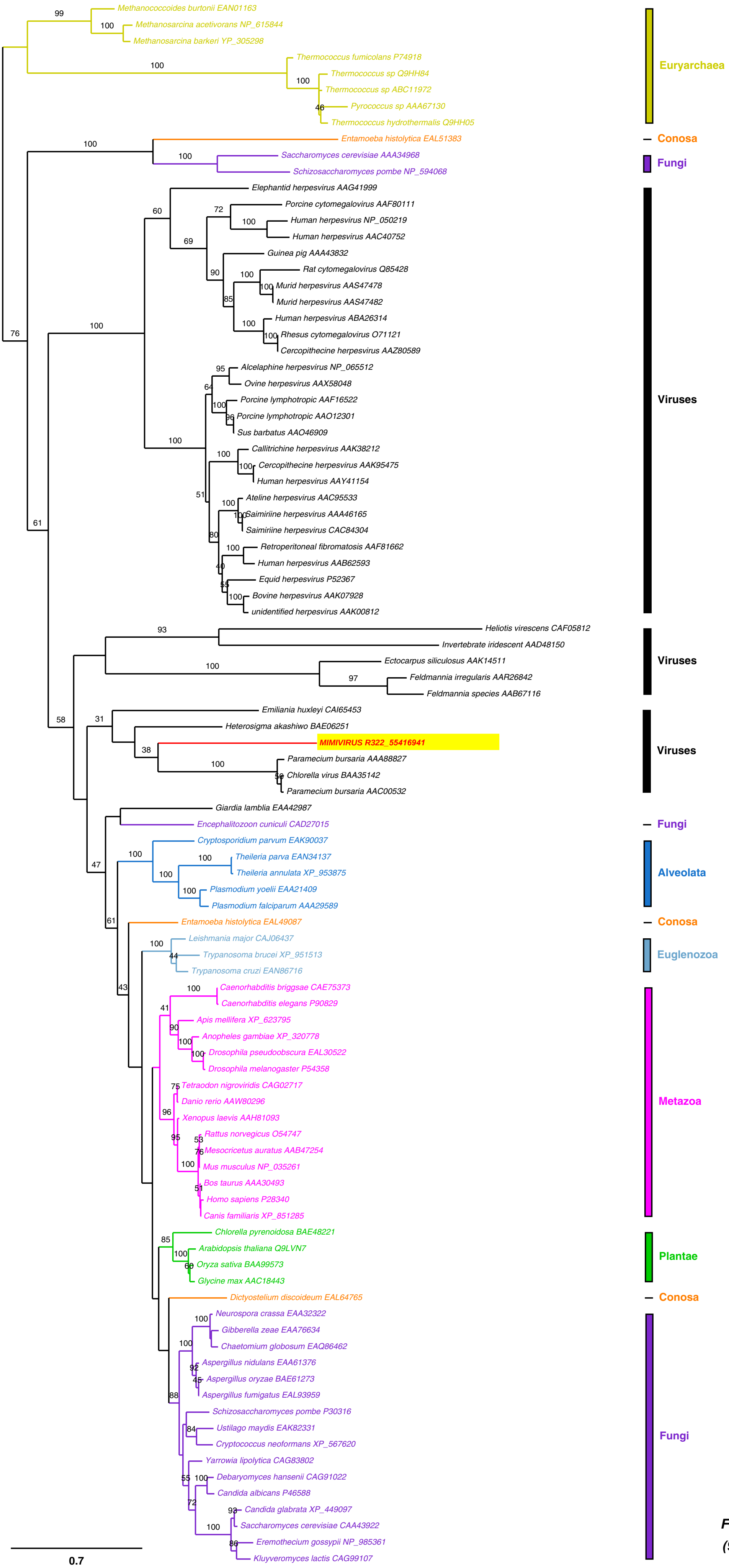

Figure 35: R322 (DNA polymerase elongation subunit, familiy B)
(96 sequences, 270 positions)

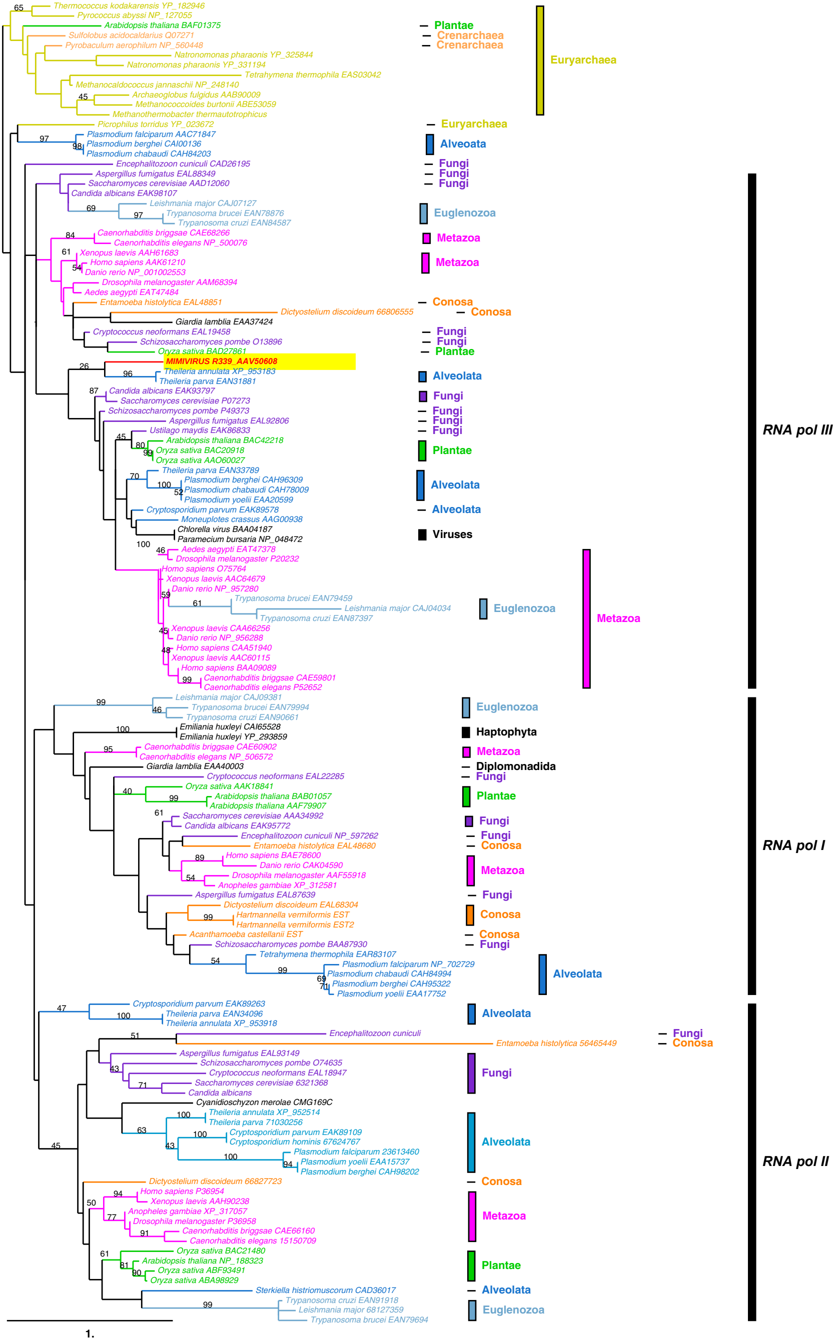

Figure 36: R339 (DNA-directed RNA polymersae, subunit M/Transcription elongation factor TFIIS) (134 sequences, 33 positions)

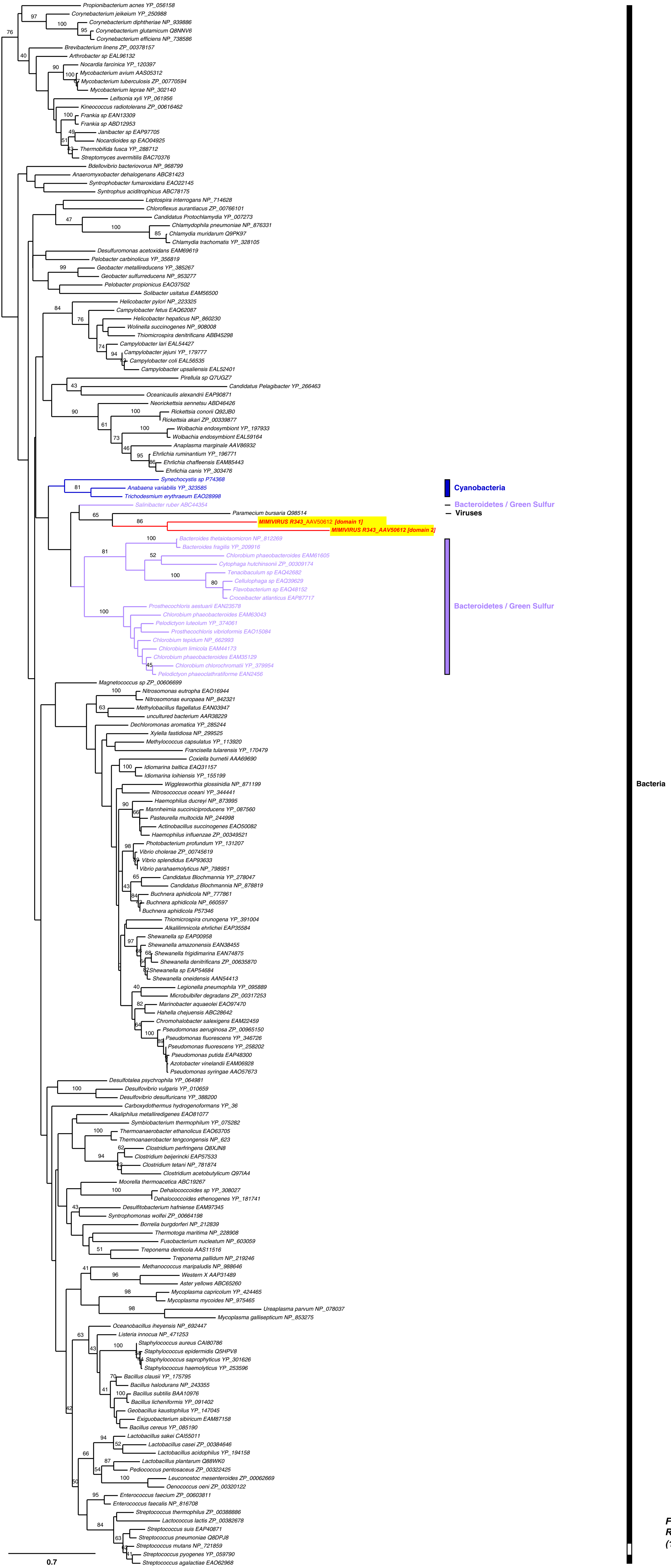

Figure 37:
R343 (dsRNA - specific Ribonuclease)
(185 sequences, 116 positions)

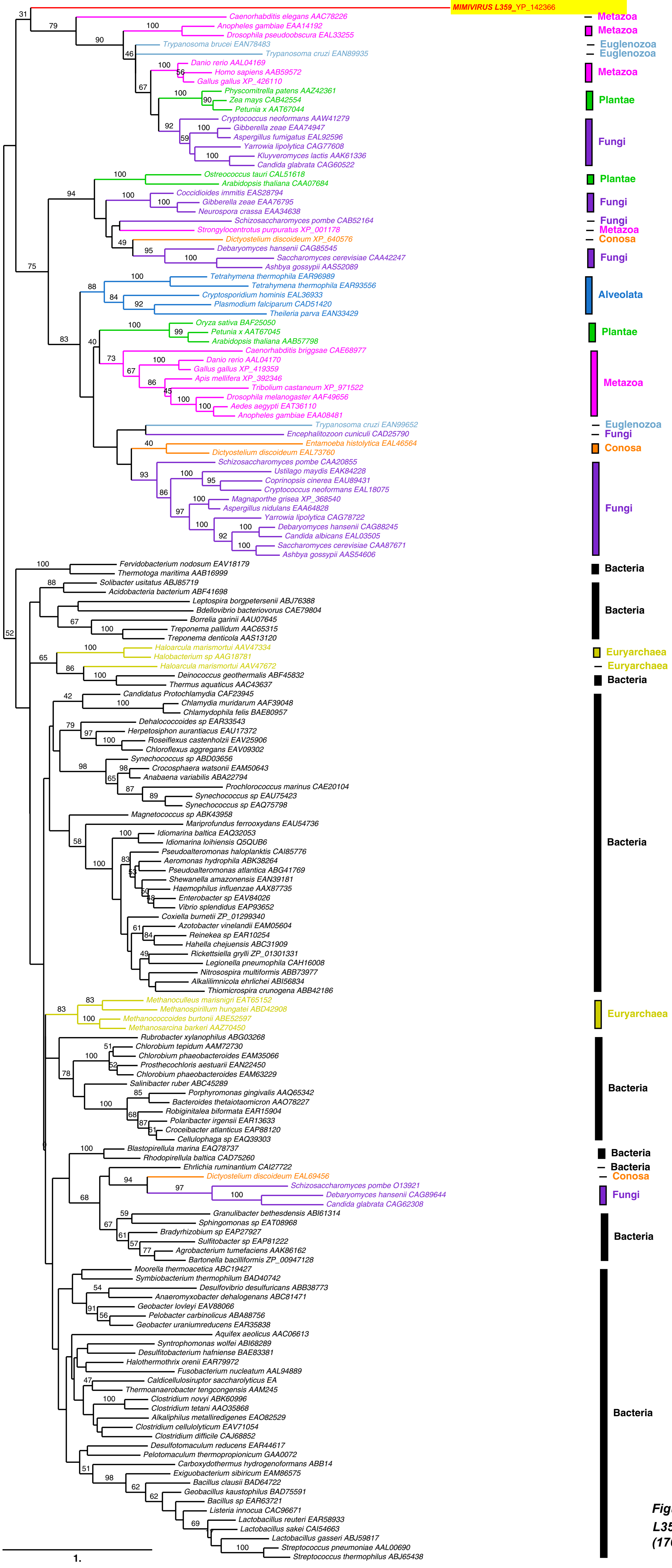

Figure 38:

L359 (Mismatch repair ATPase)
(176 sequences, 253 positions)

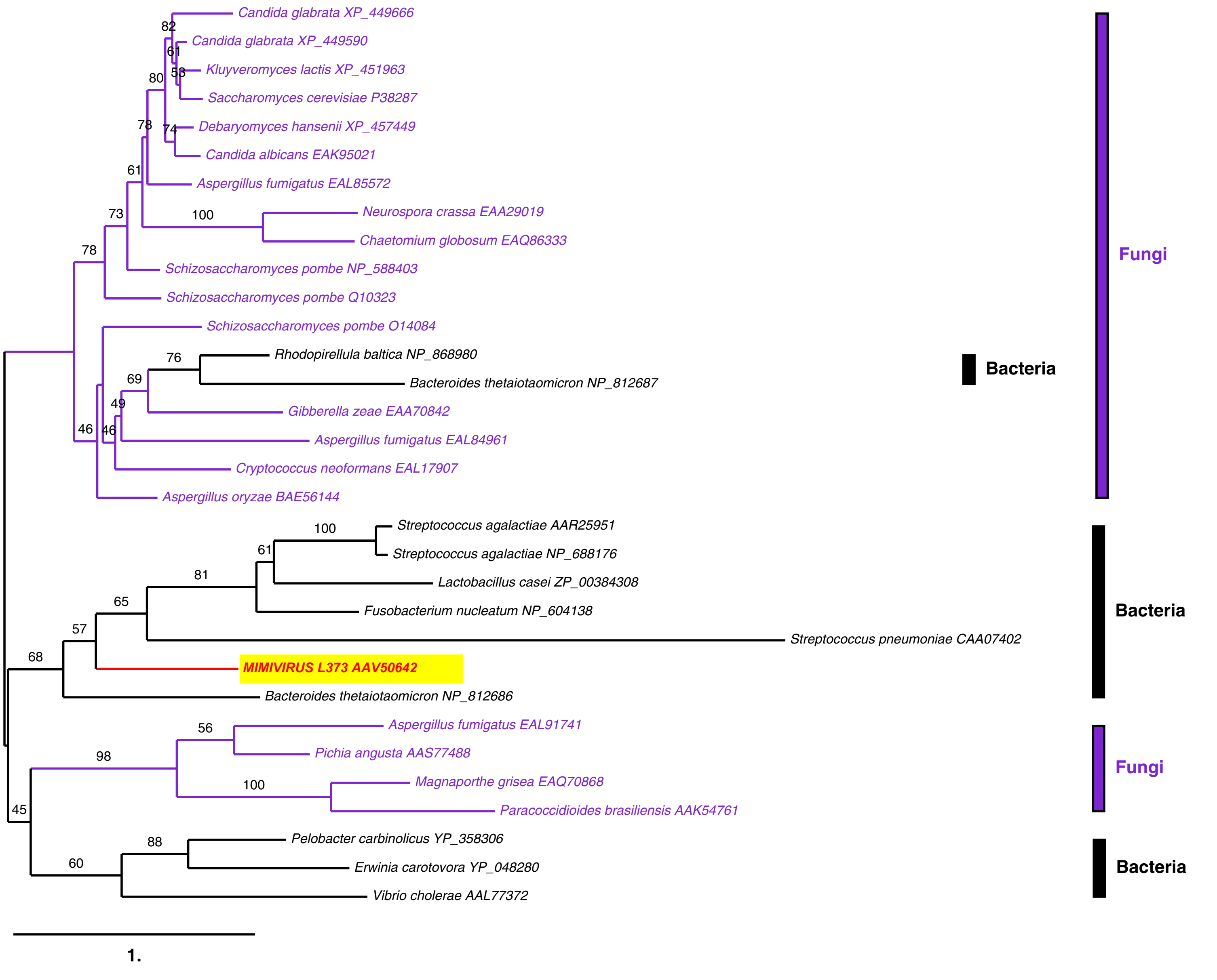

Figure 39: L373 (Mannosyltransferase OCH1 and related enzymes) (32 sequences, 77 positions)

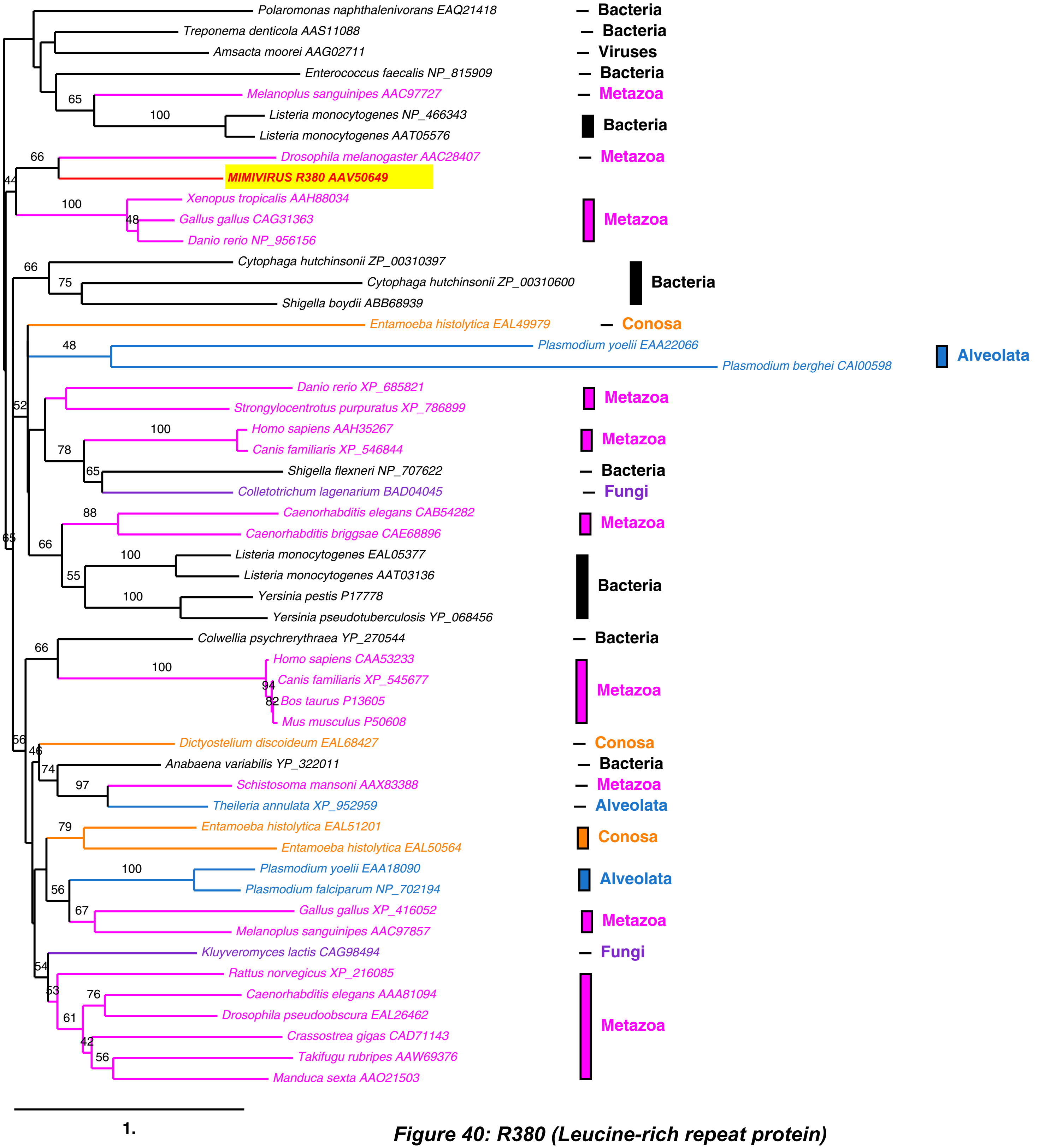

Figure 40: R380 (Leucine-rich repeat protein)
(52 sequences, 140 positions)

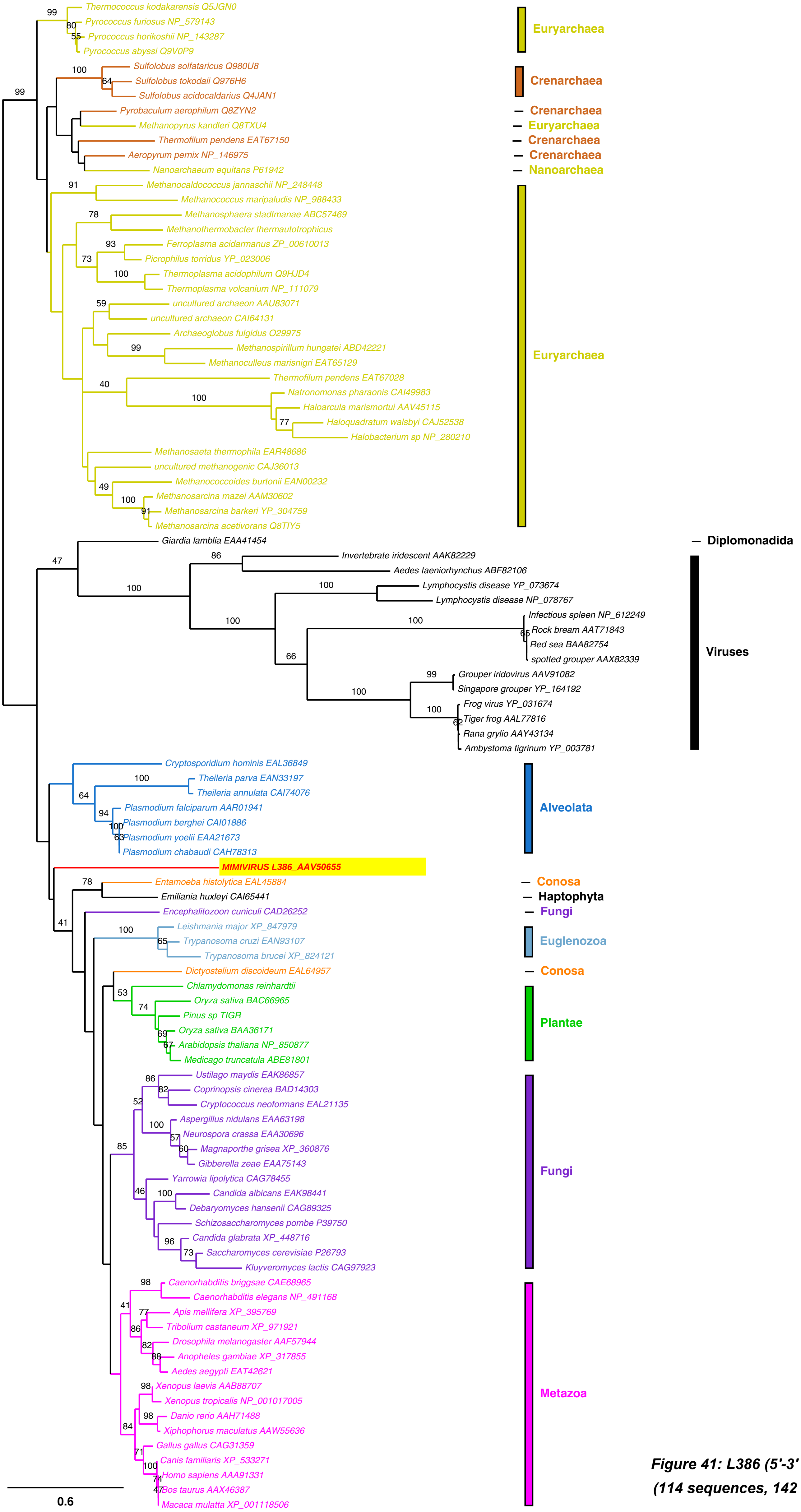

Figure 41: L386 (5'-3' exonuclease)
(114 sequences, 142 positions)

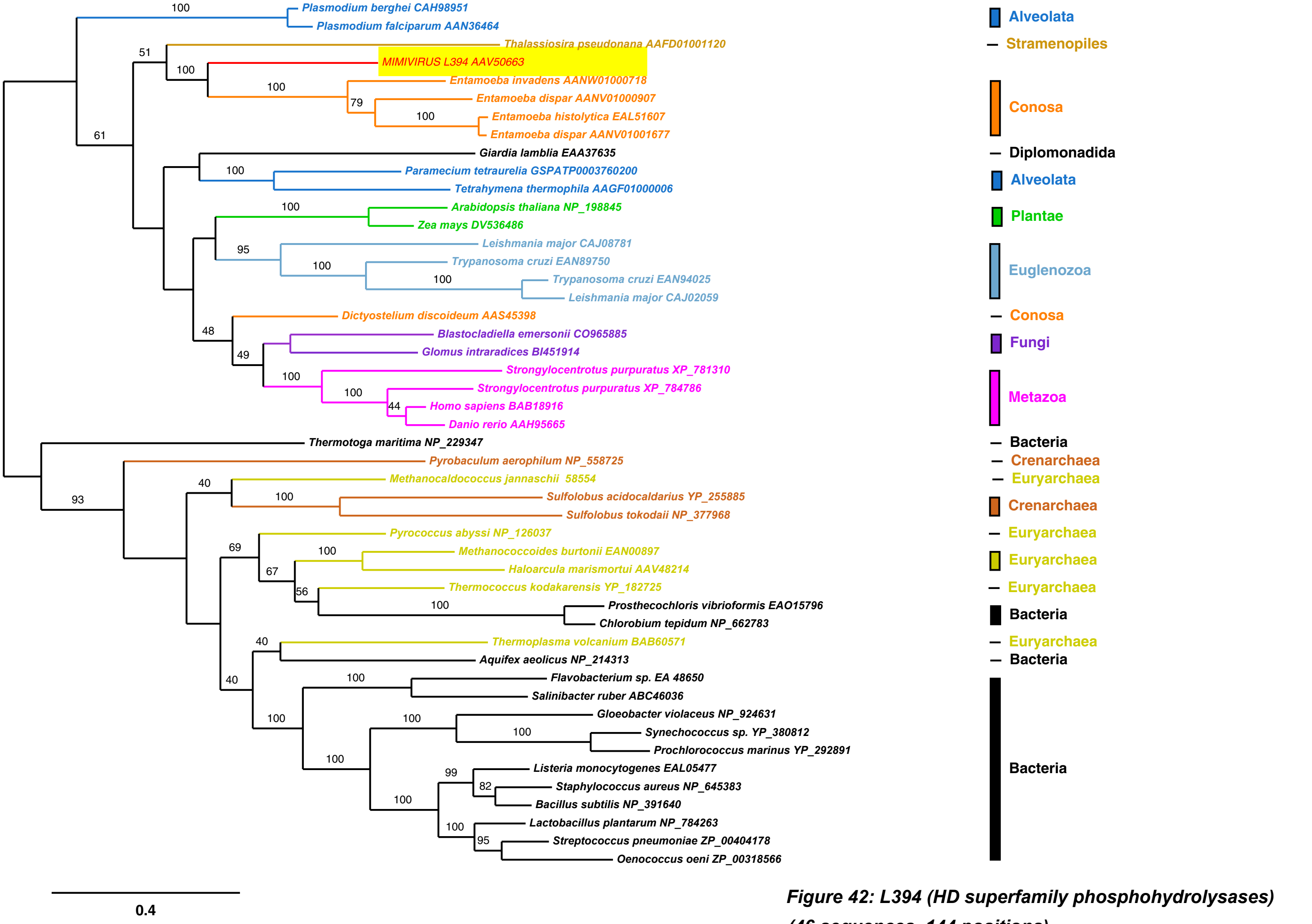

Figure 42: L394 (HD superfamily phosphohydrolysases)
(46 sequences, 144 positions)

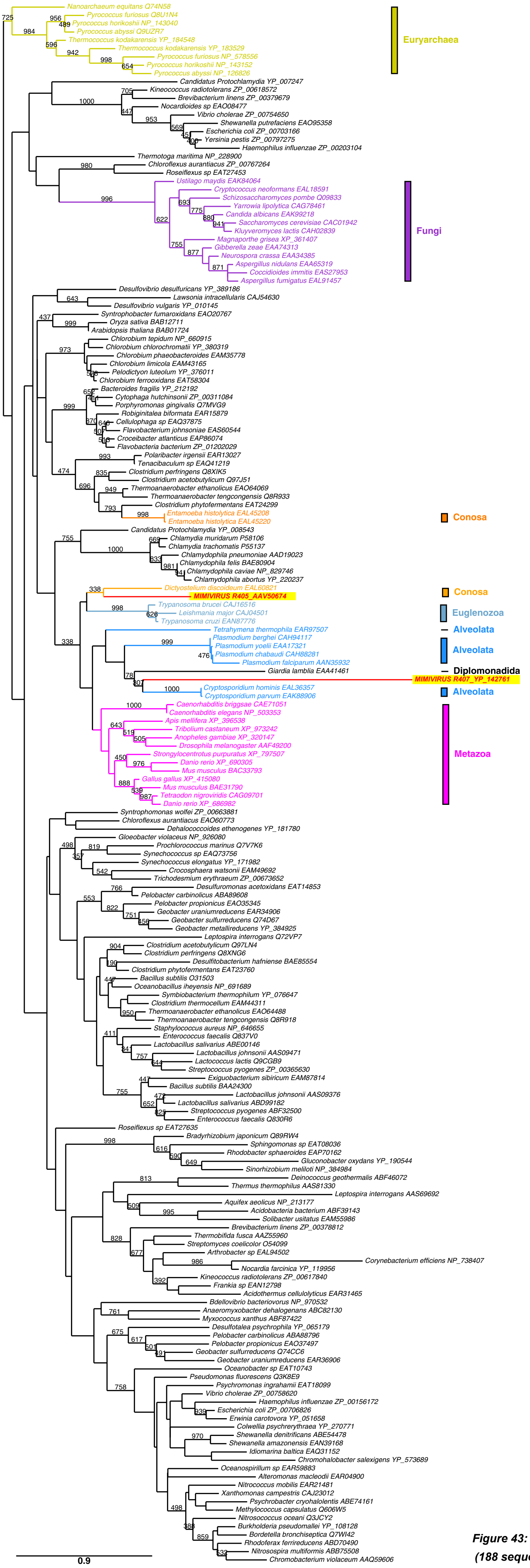

Figure 43: R405/R407 (Sam-dependent methyltransferases related to tRNA (uracil-5) methyltransferase)
(188 sequences, 94 positions)

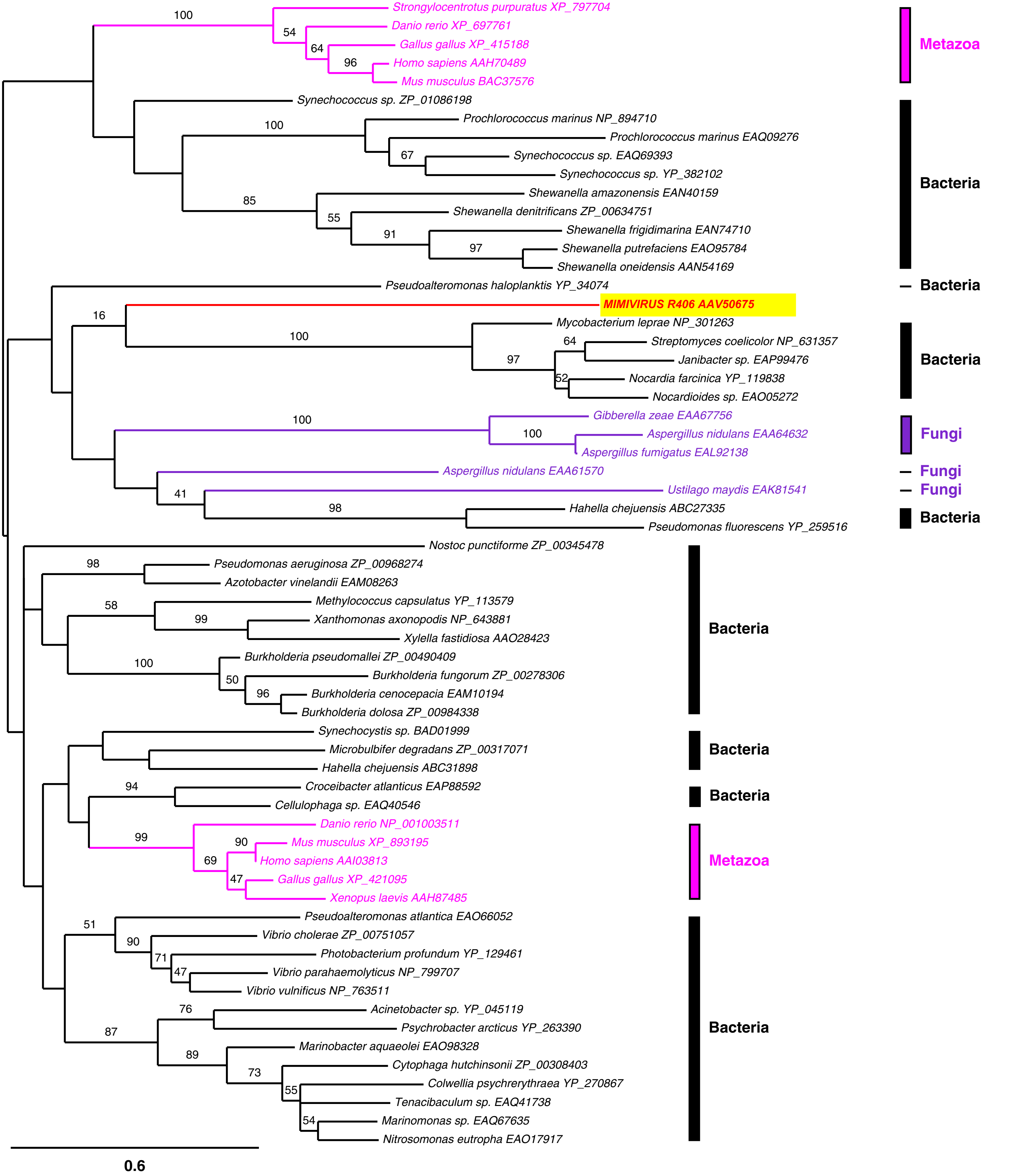

Figure 44: R406 (Alkylated NA repair protein)

(62 sequences, 140 positions)

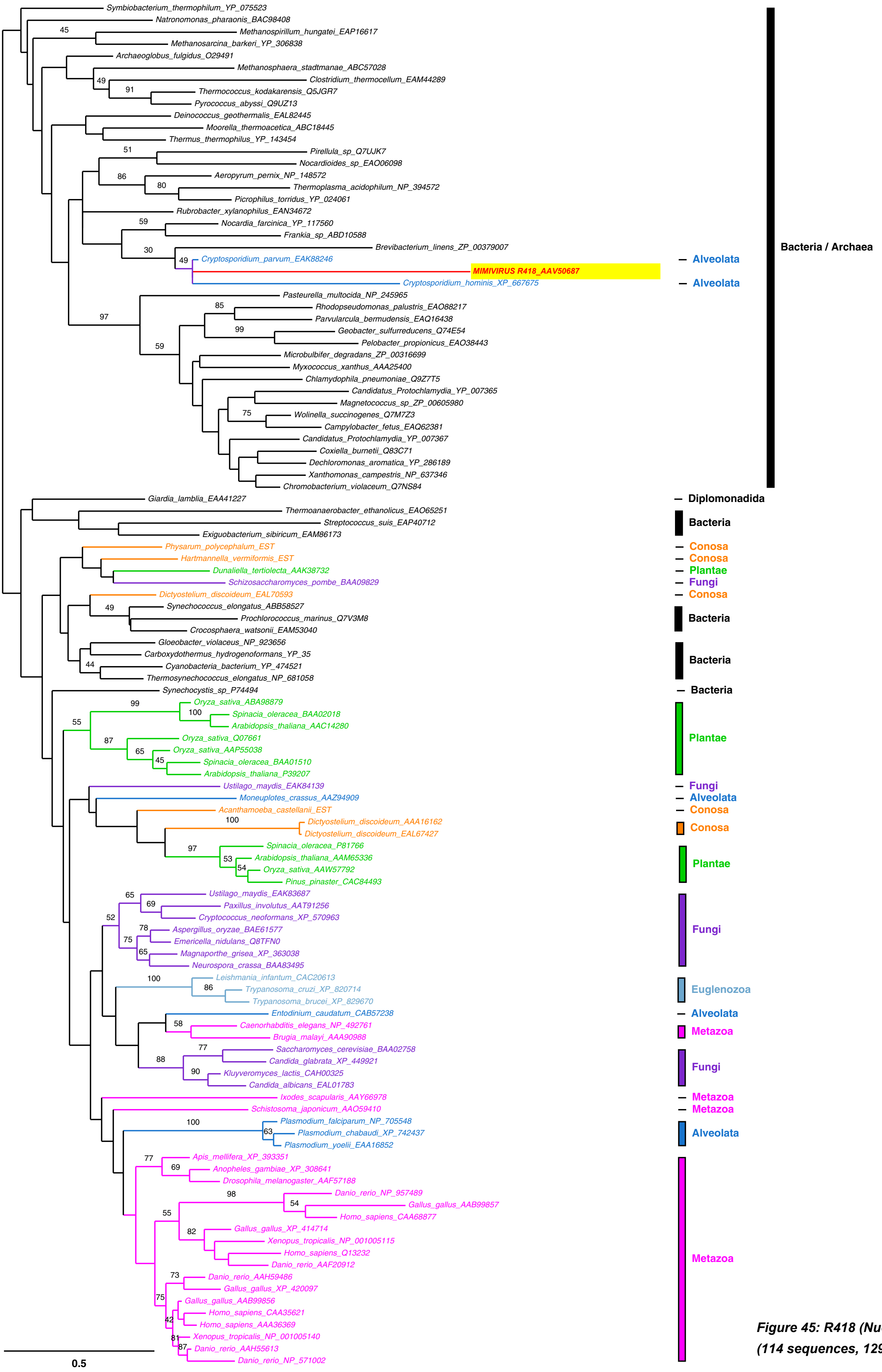

Figure 45: R418 (Nucleoside diphosphate kinase)
(114 sequences, 129 positions)

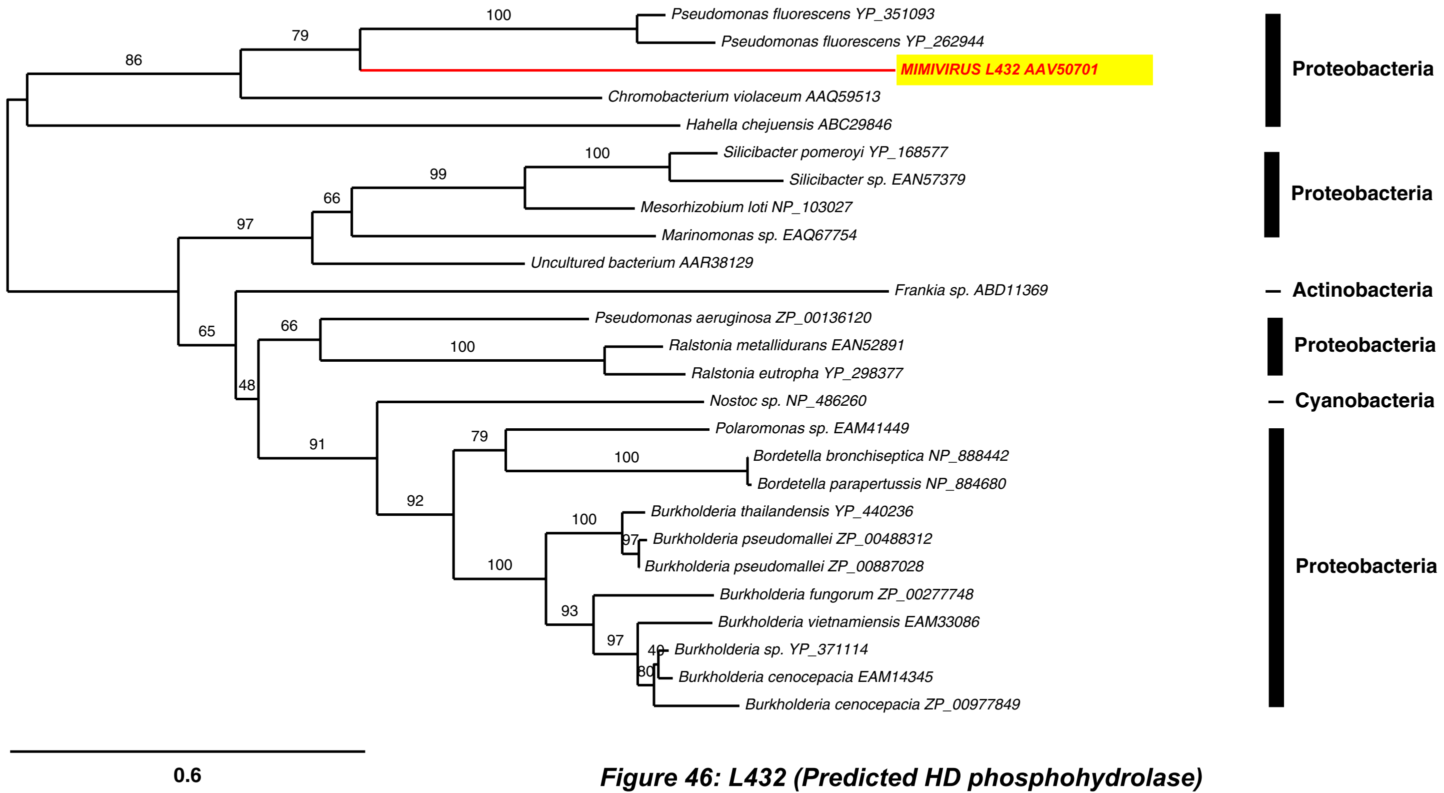

Figure 46: L432 (Predicted HD phosphohydrolase)

(26 sequences, 152 positions)

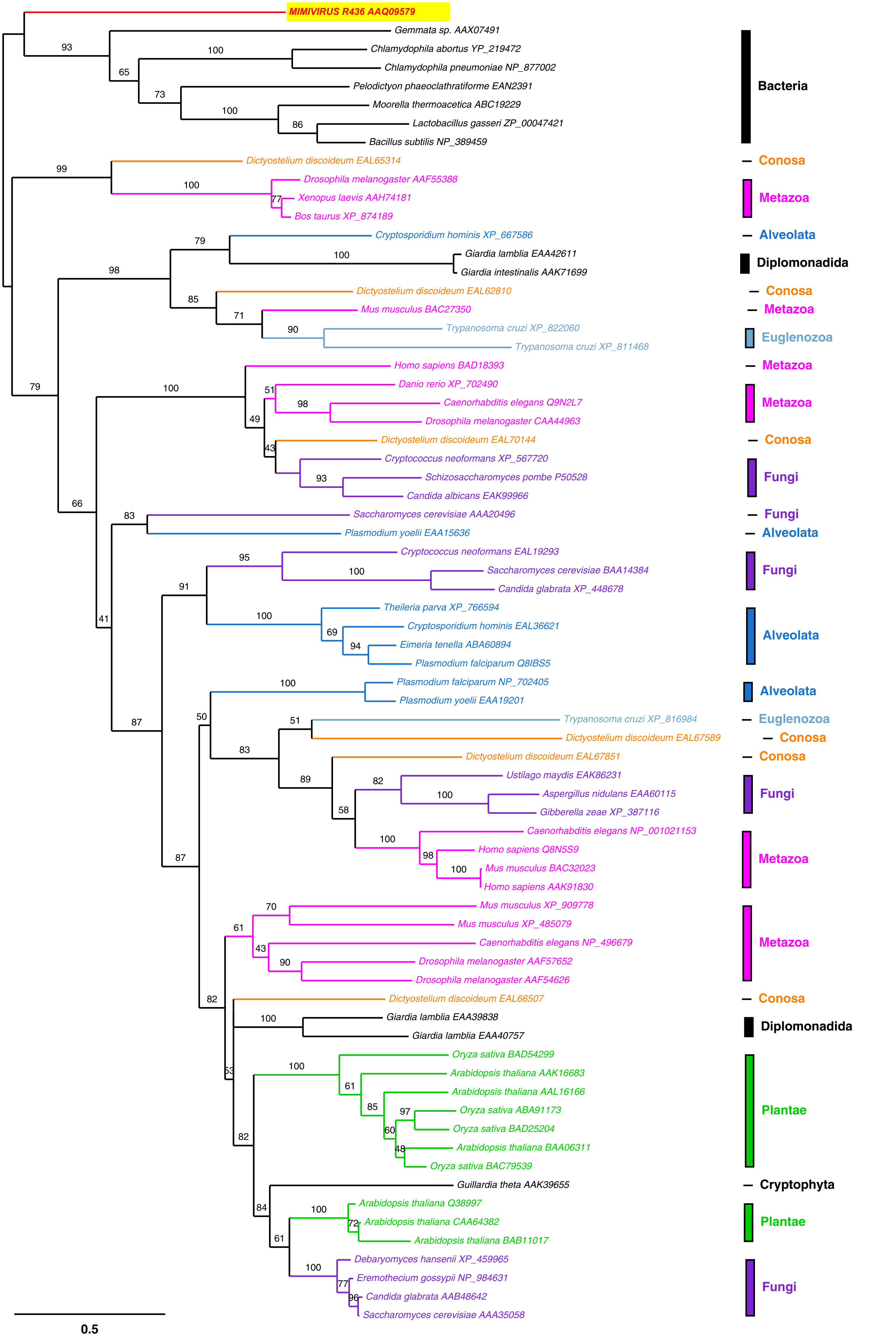

Figure 47: R436 (Serine / threonine protein kinase)
(71 sequences, 196 positions)

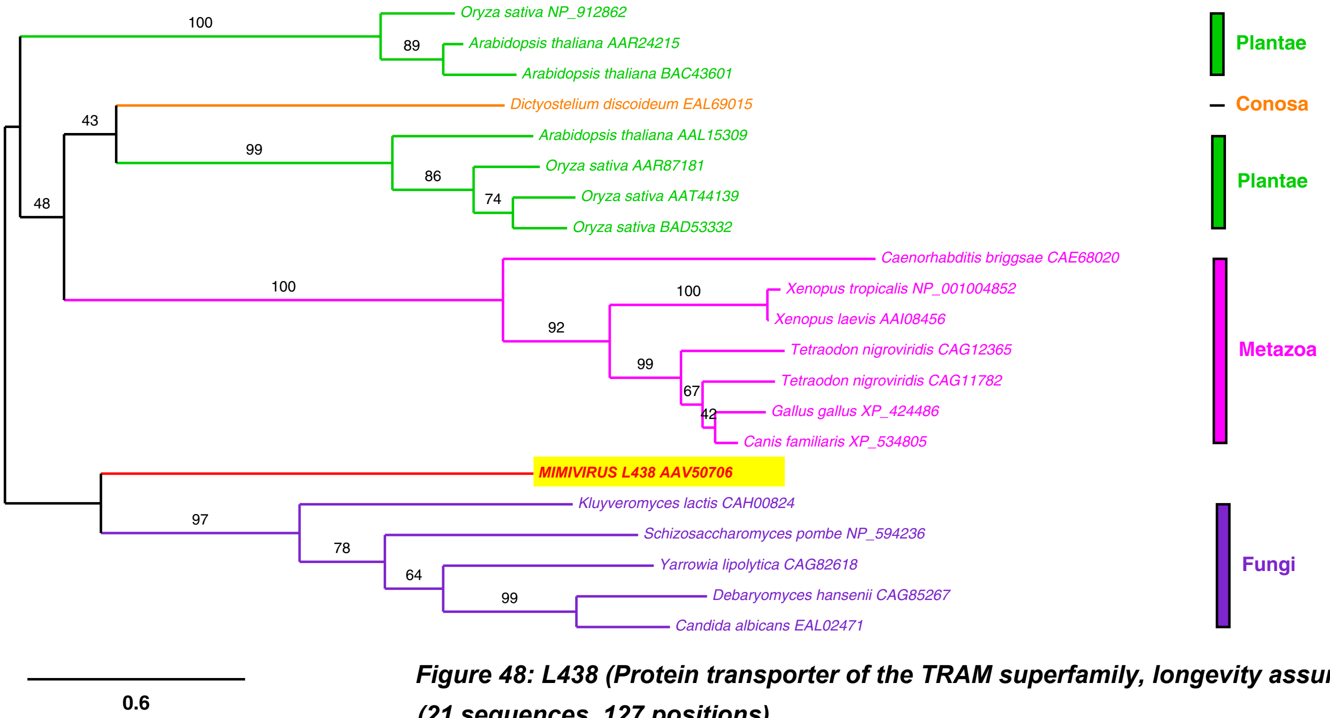

Figure 48: L438 (Protein transporter of the TRAM superfamily, longevity assurance factor) (21 sequences, 127 positions)

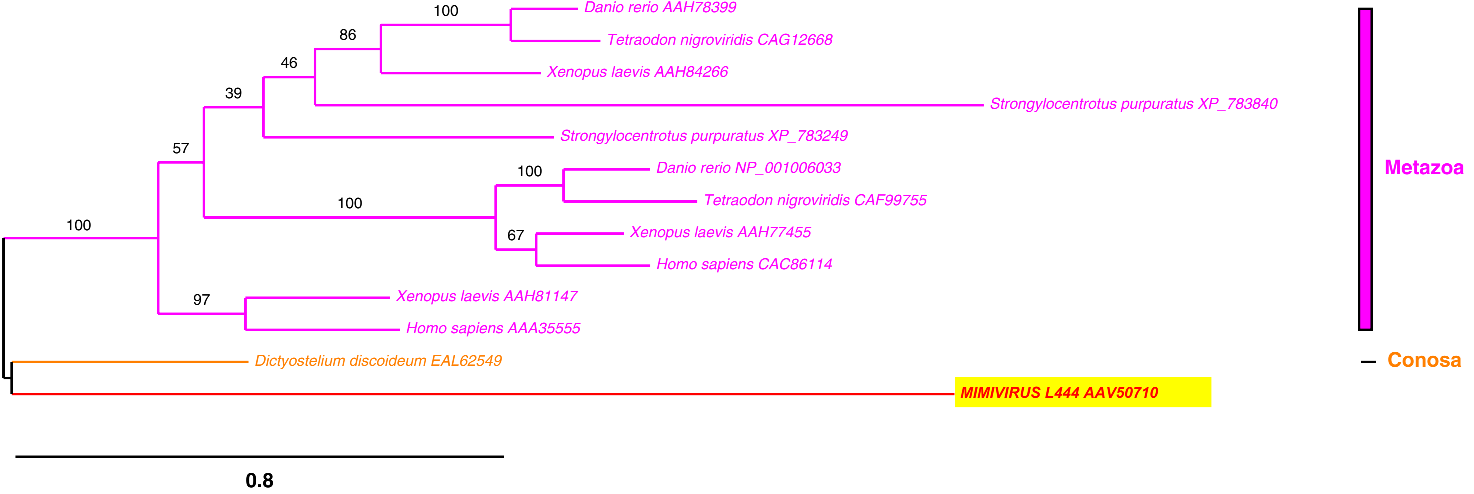

Figure 49: L444 (ADP-ribosylglycohydrolase)

(13 sequences, 316 positions)

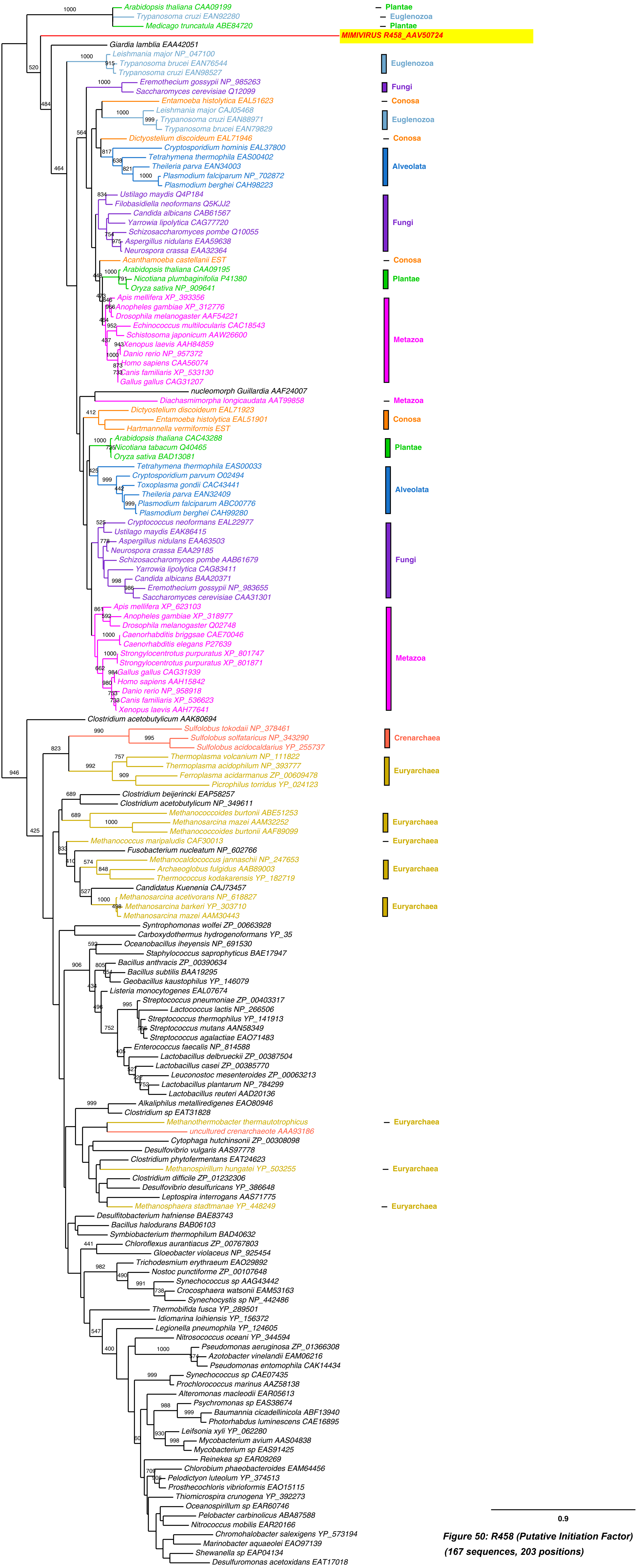

Figure 50: R458 (Putative Initiation Factor)
(167 sequences, 203 positions)

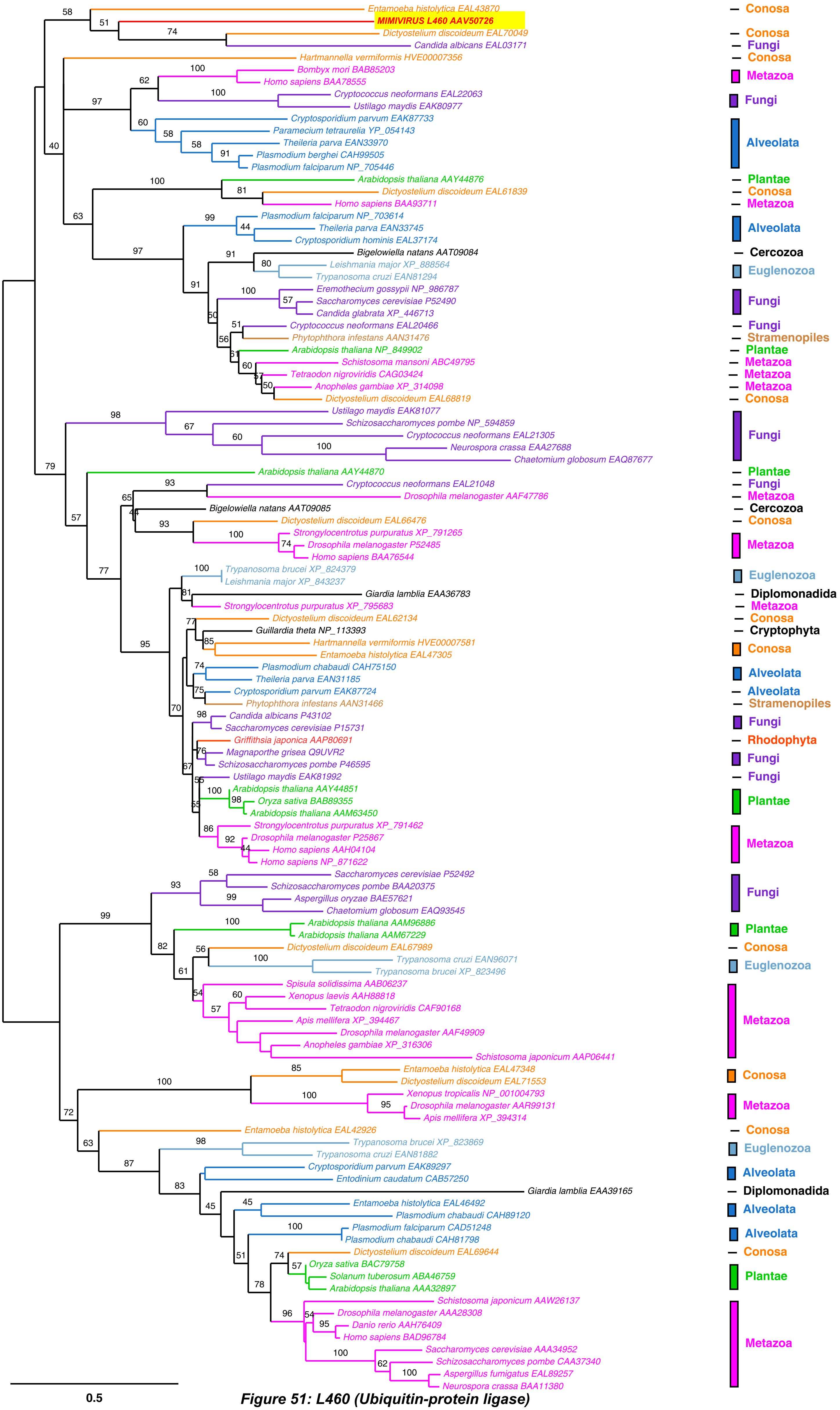

Figure 51: L460 (Ubiquitin-protein ligase)

(114 sequences, 105 positions)

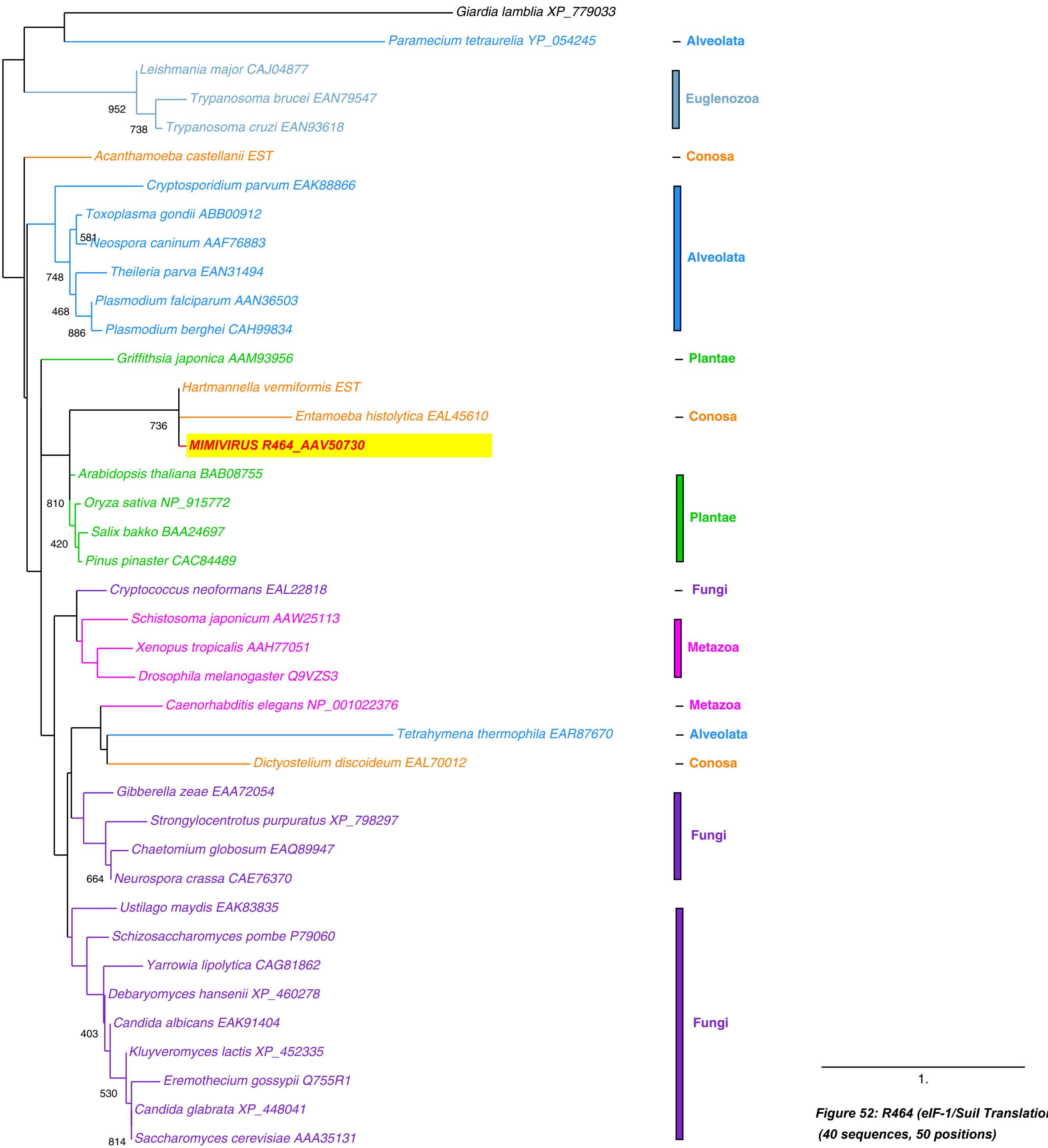

Figure 52: R464 (eIF-1/Suil Translation Initiation Factor)
(40 sequences, 50 positions)

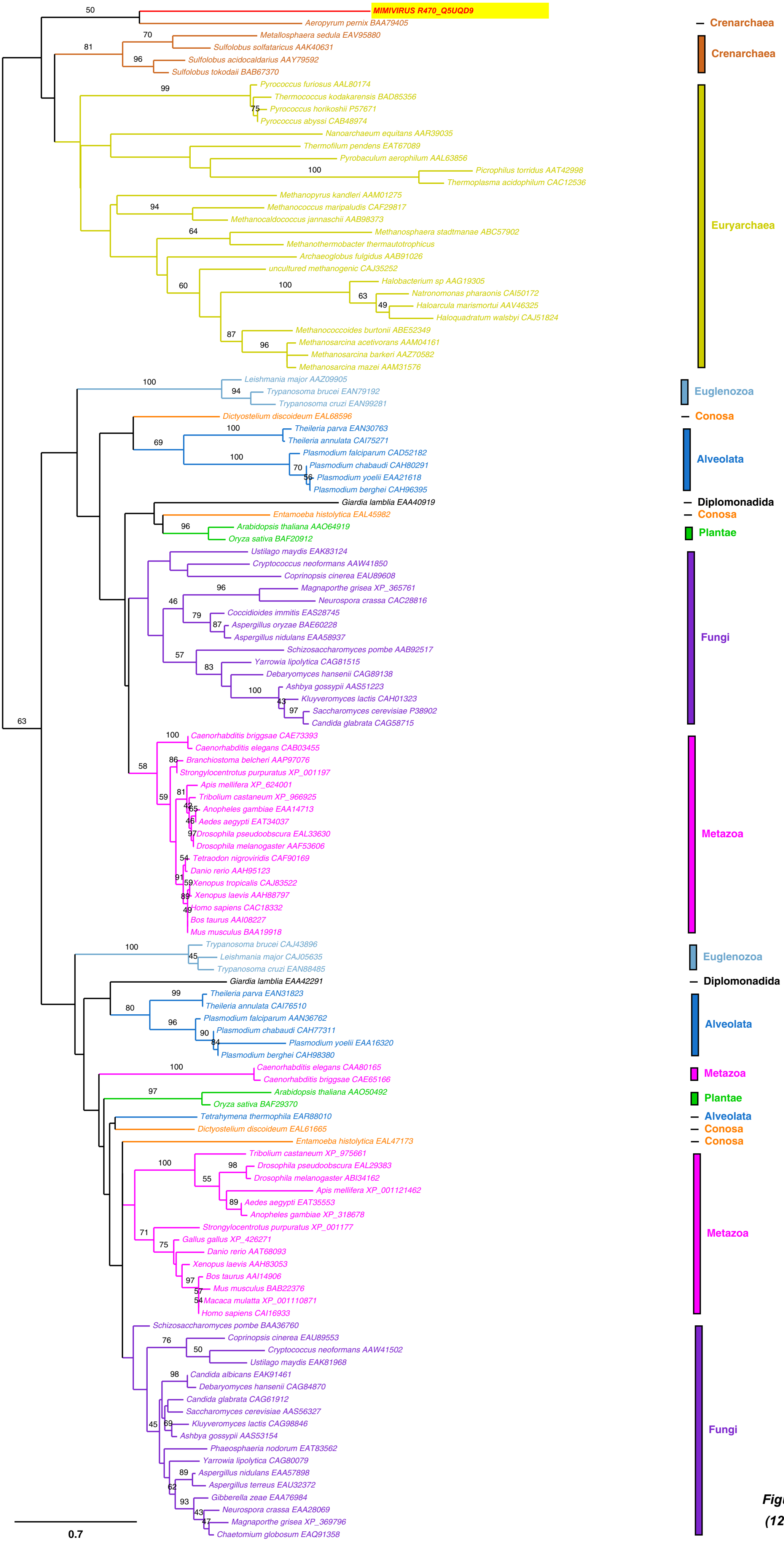

Figure 53: R470 (DNA-directed RNA polymerase, subunit L)
(125 sequences, 72 positions)

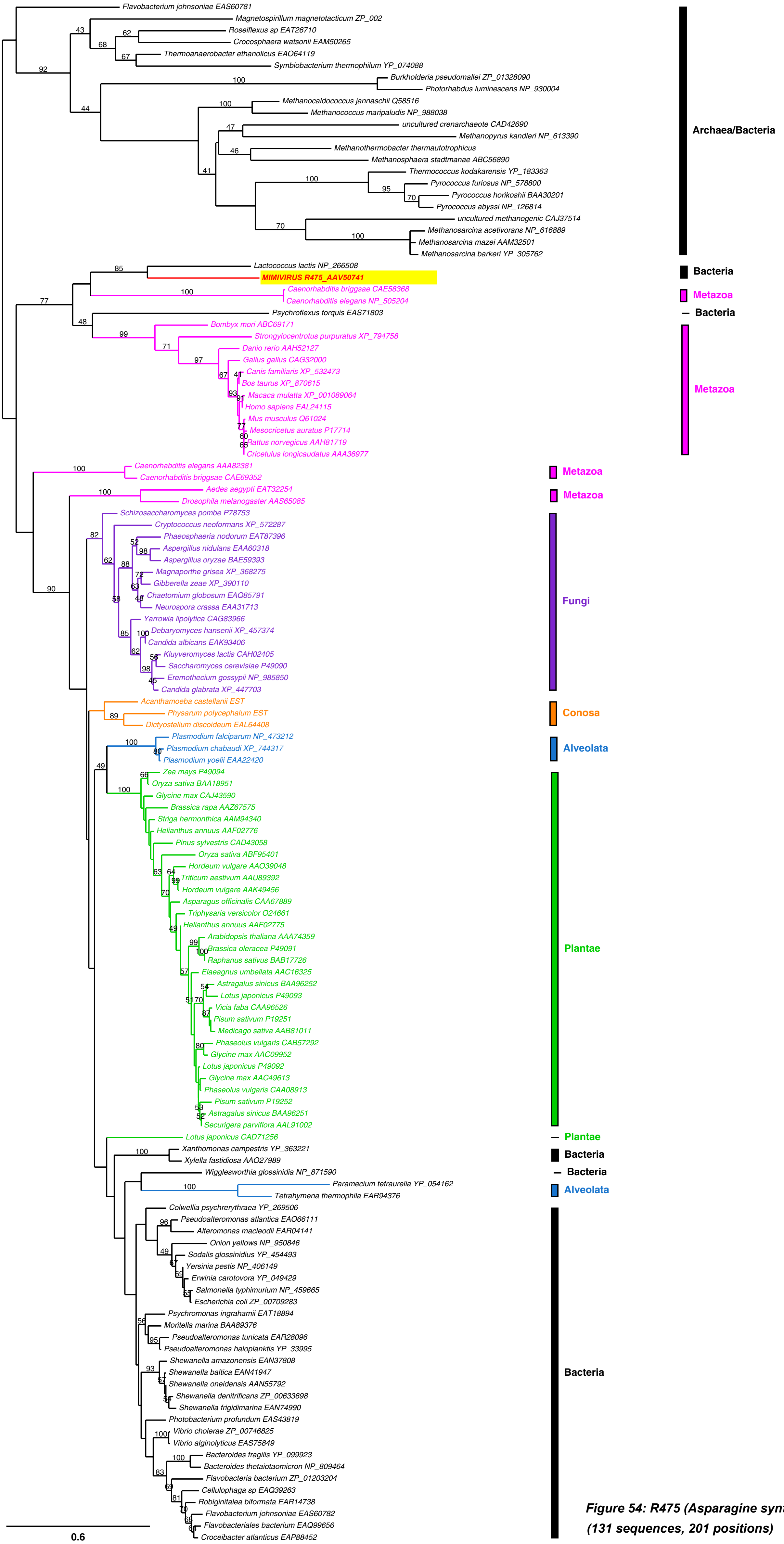

Figure 54: R475 (Asparagine synthase (glutamine-hydrolyzing)) (131 sequences, 201 positions)

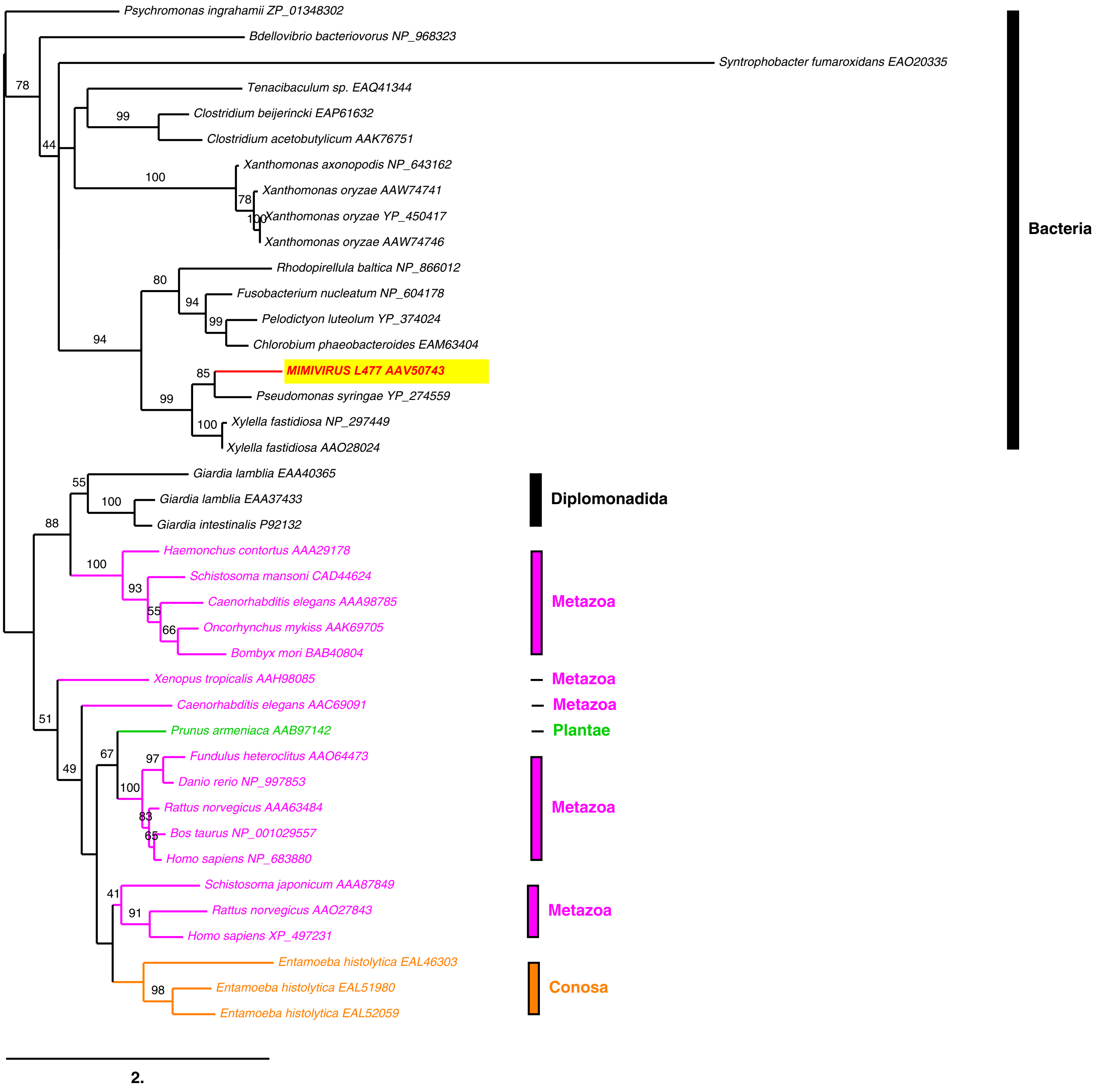

Figure 55: L477 (Cysteine protease)

(40 sequences, 162 positions)

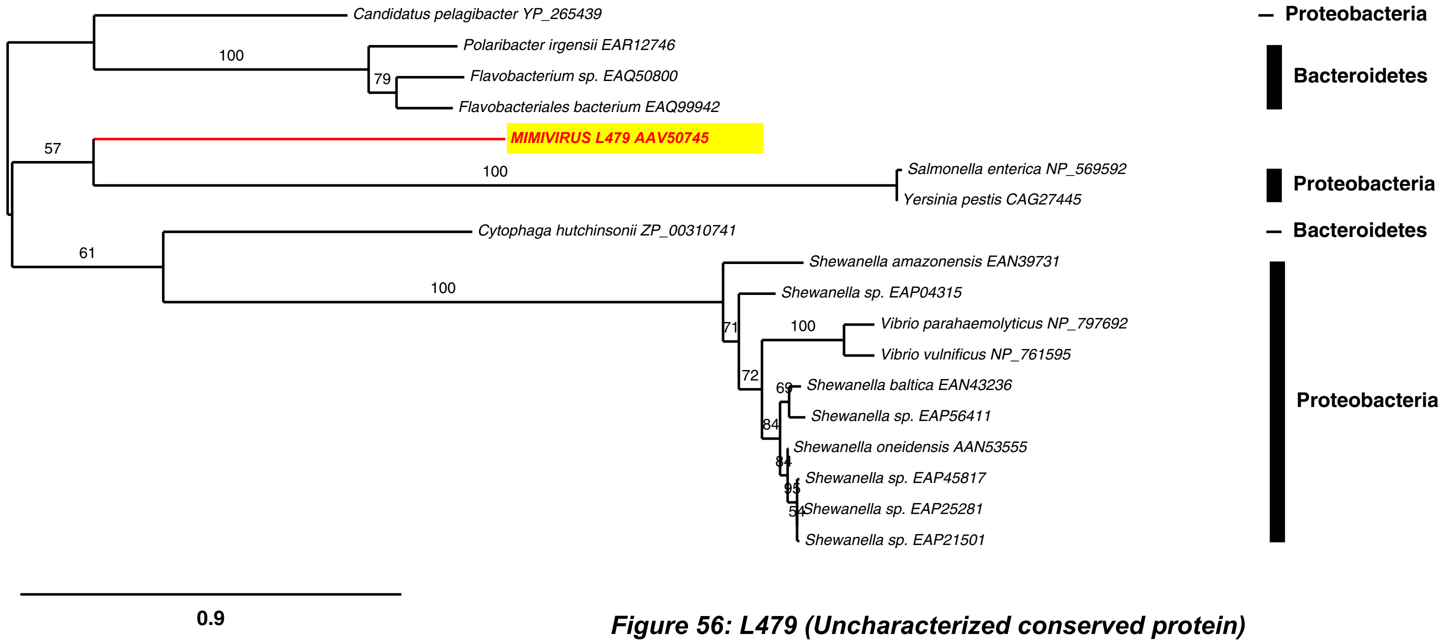

Figure 56: L479 (Uncharacterized conserved protein)
(18 sequences, 181 positions)

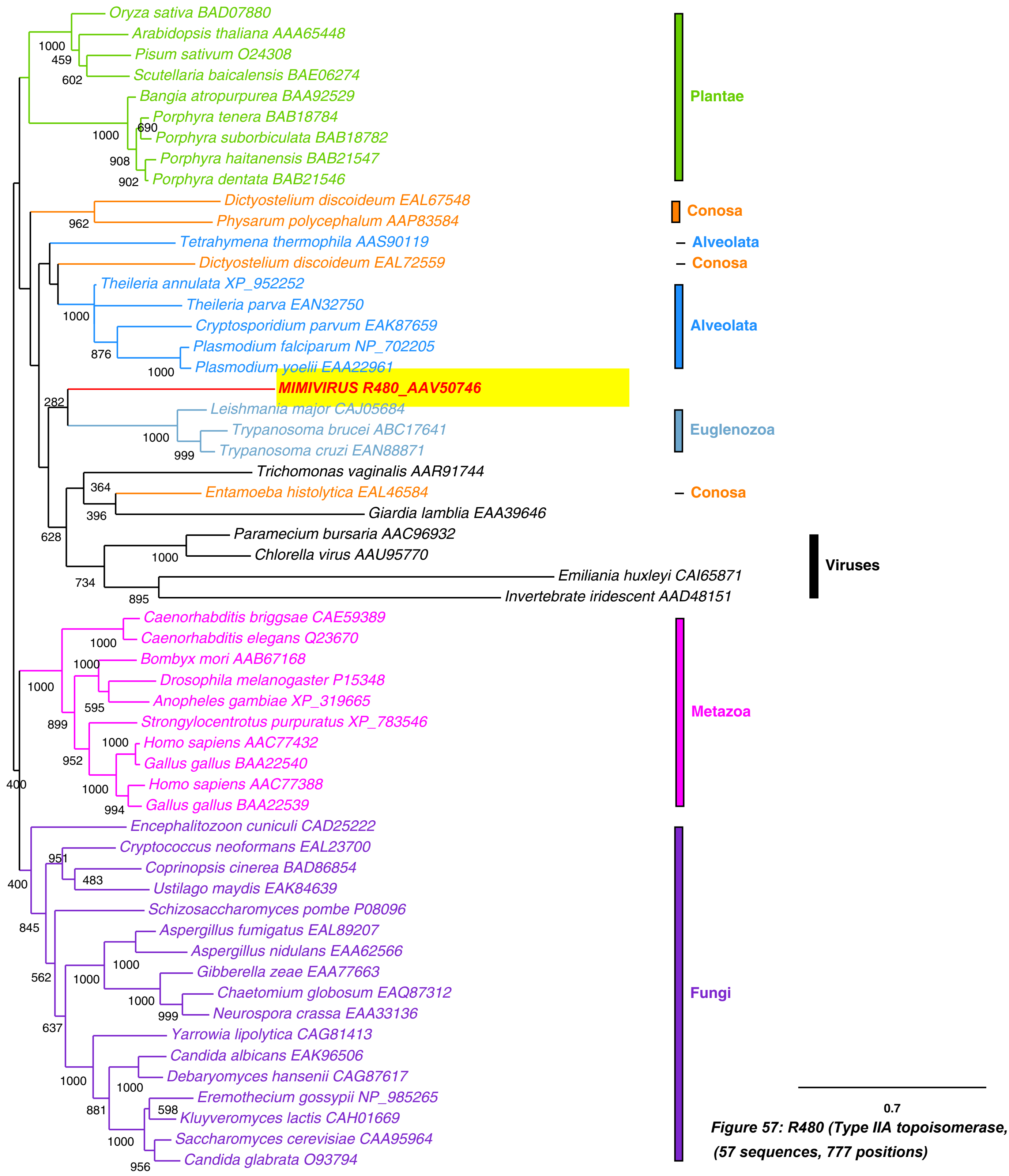

Figure 57: R480 (Type IIA topoisomerase, B subunit)
(57 sequences, 777 positions)

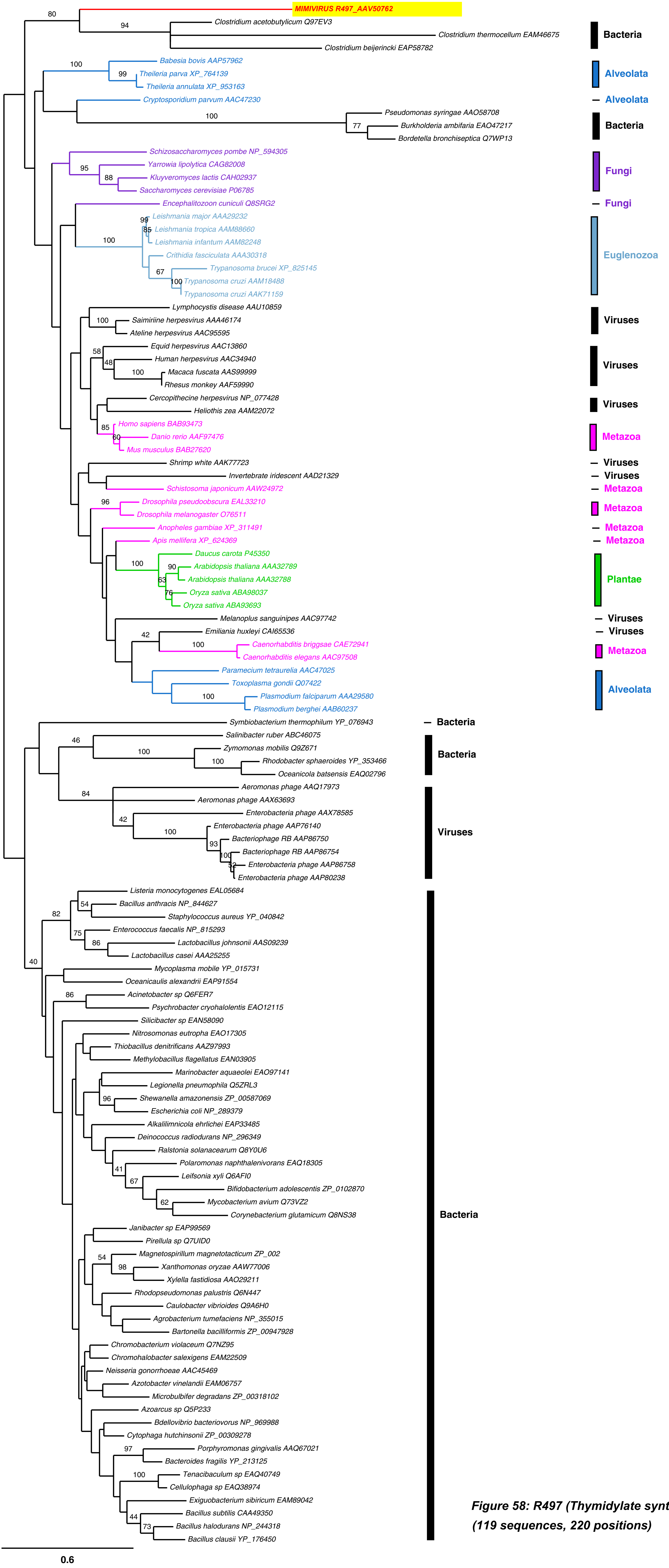

Figure 58: R497 (Thymidylate synthase)
(119 sequences, 220 positions)

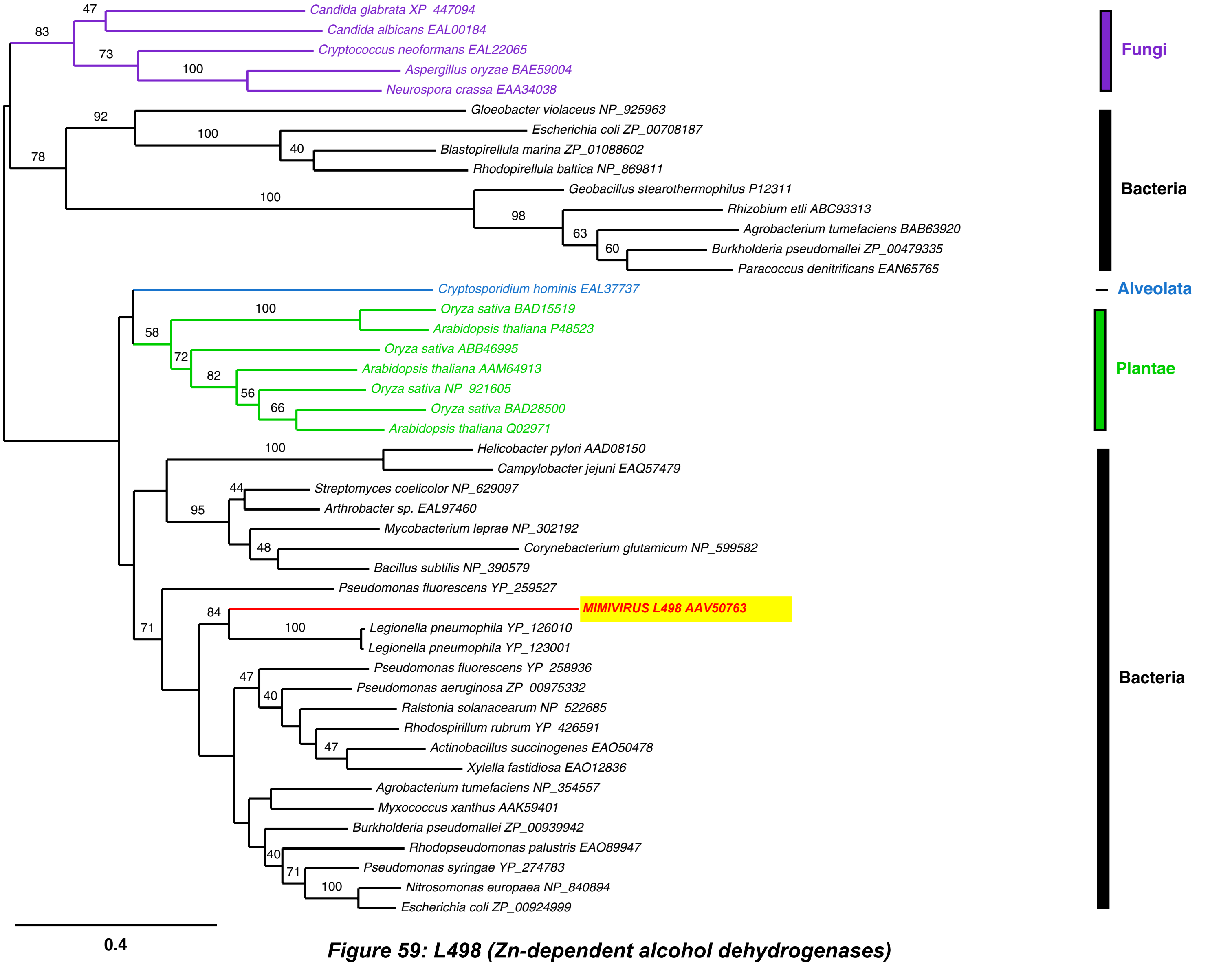

Figure 59: L498 (Zn-dependent alcohol dehydrogenases)

(46 sequences, 233 positions)

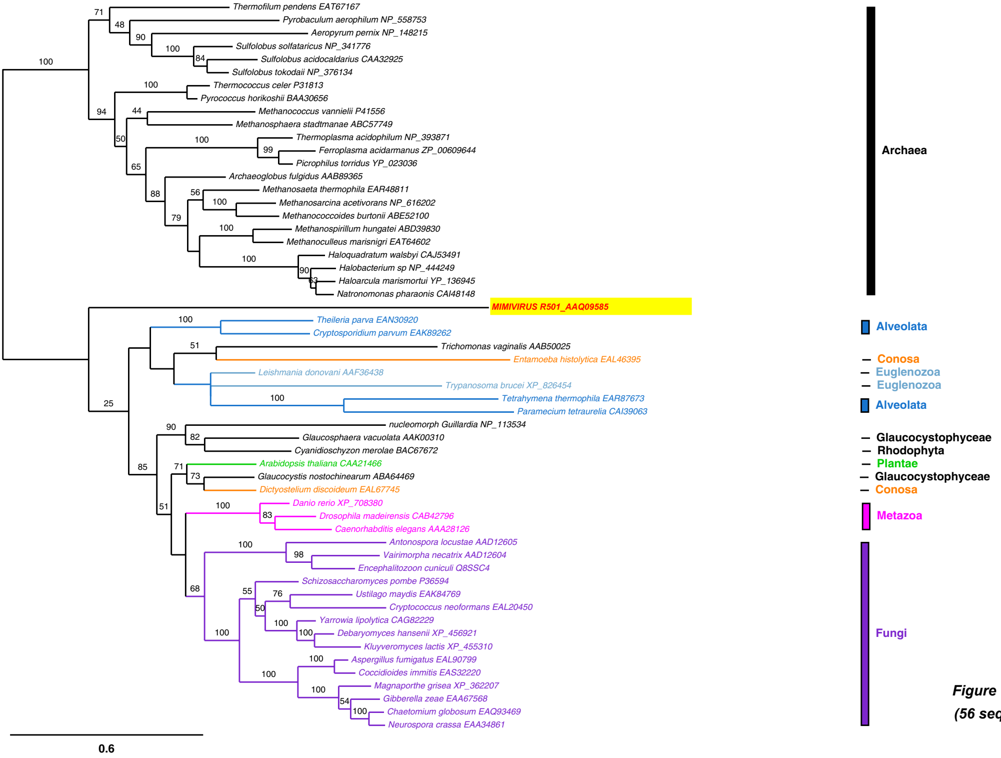

Figure 60: R501 (DNA-directed RNA polymerase, beta' subunit/160kD subunit) (56 sequences, 655 positions)

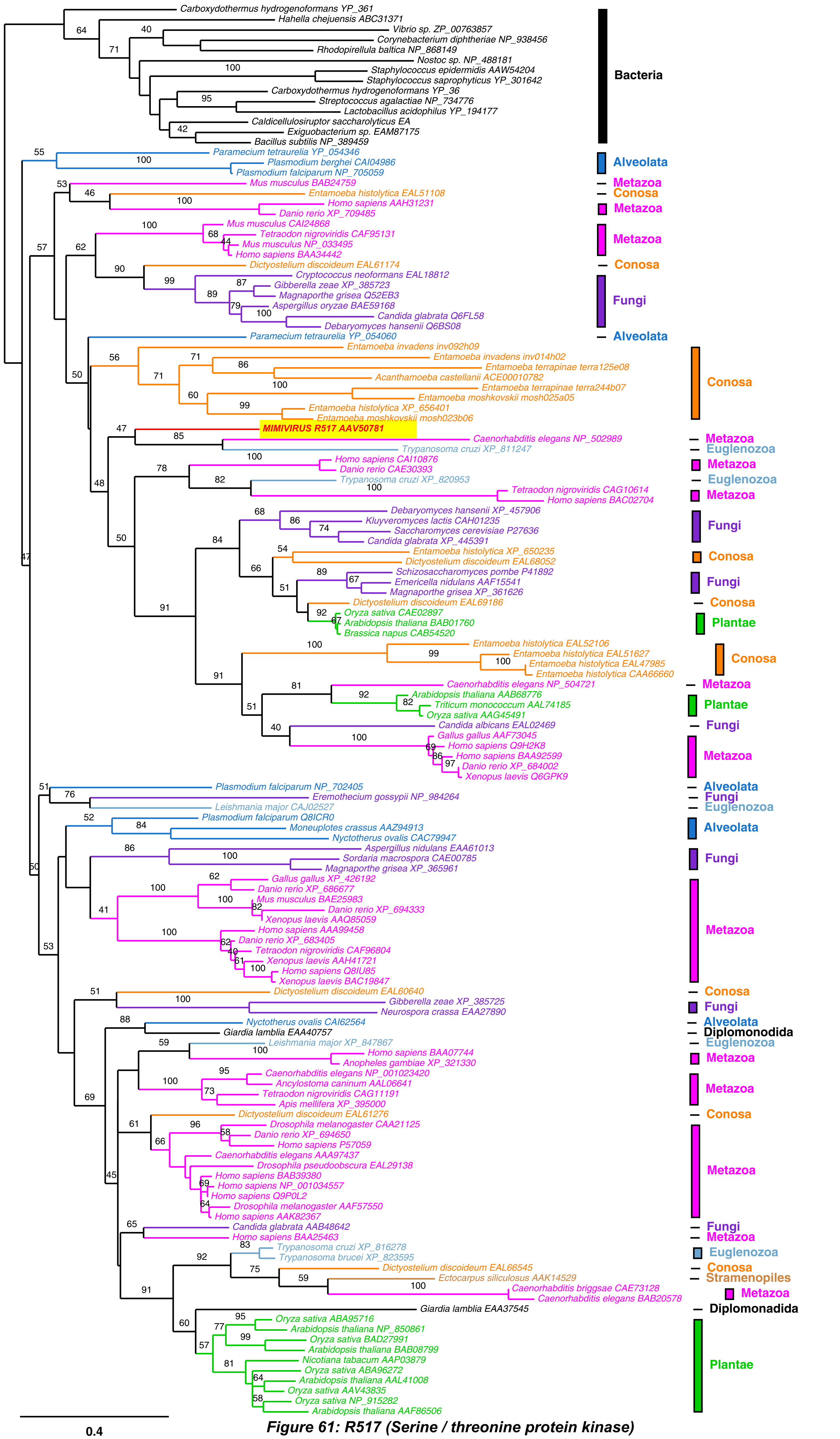

Figure 61: R517 (Serine / threonine protein kinase)

(138 sequences, 84 positions)

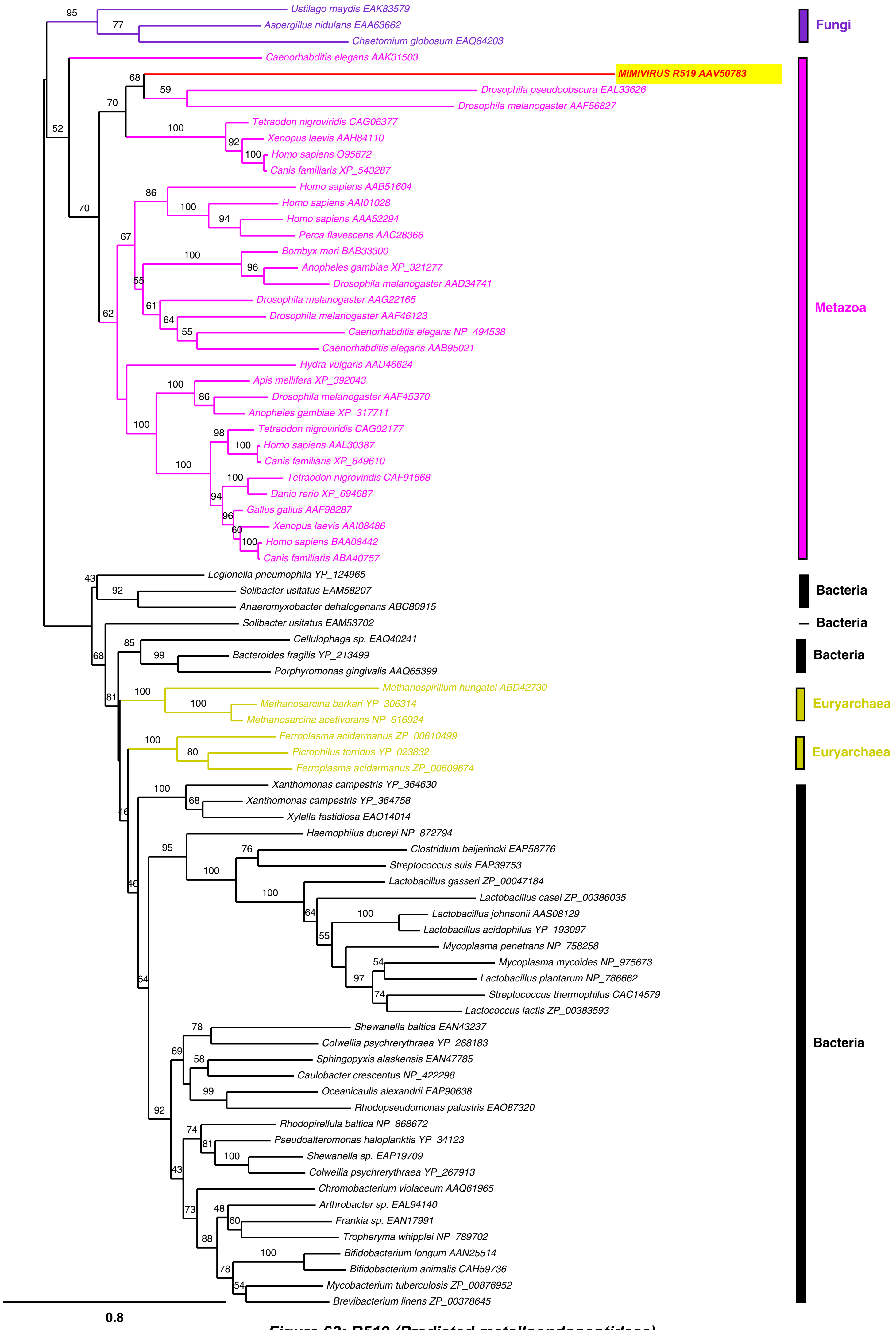

Figure 62: R519 (Predicted metalloendopeptidase)

(81 sequences, 339 positions)

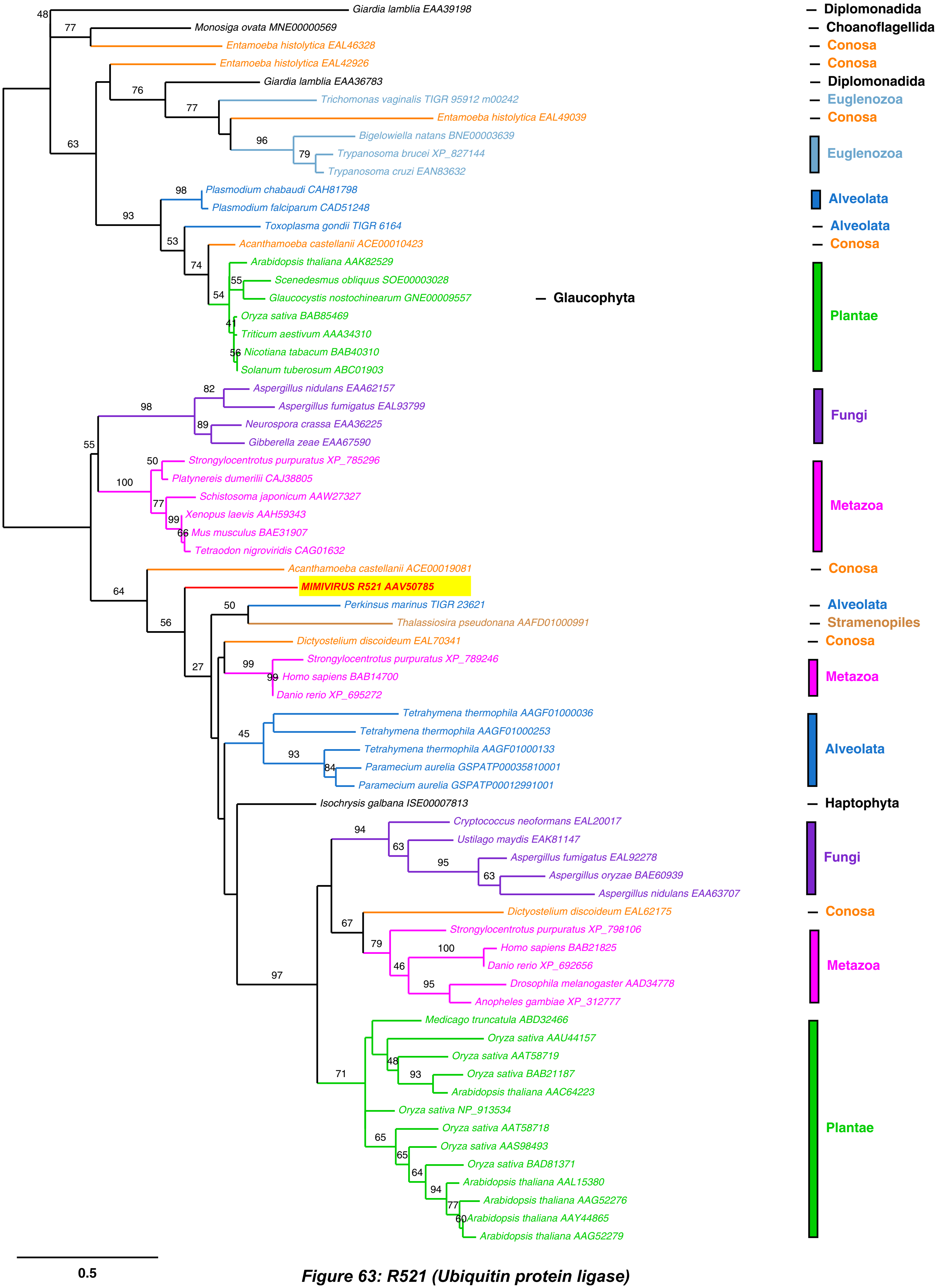

Figure 63: R521 (Ubiquitin protein ligase)

(69 sequences, 91 positions)

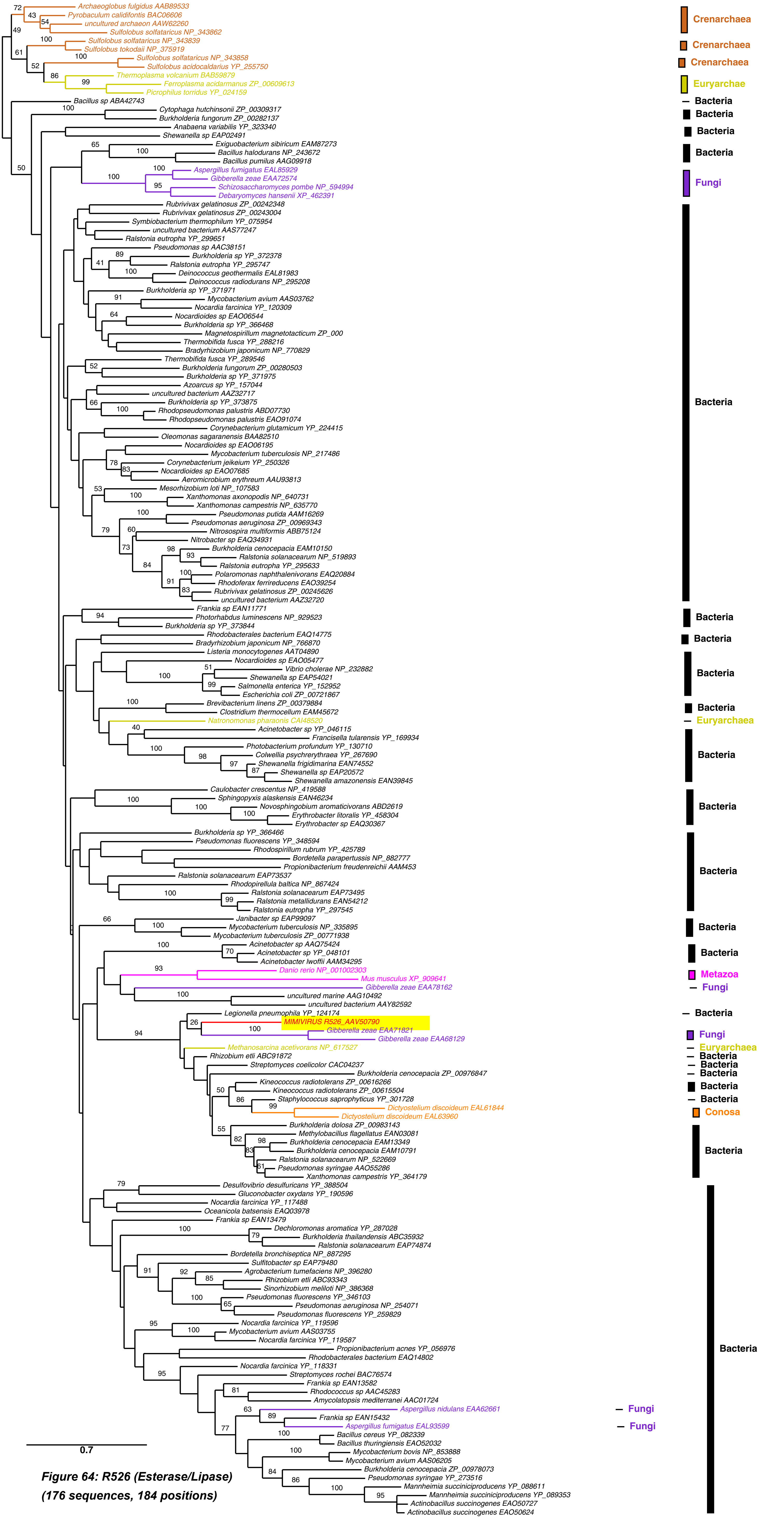

Figure 64: R526 (Esterase/Lipase)
(176 sequences, 184 positions)

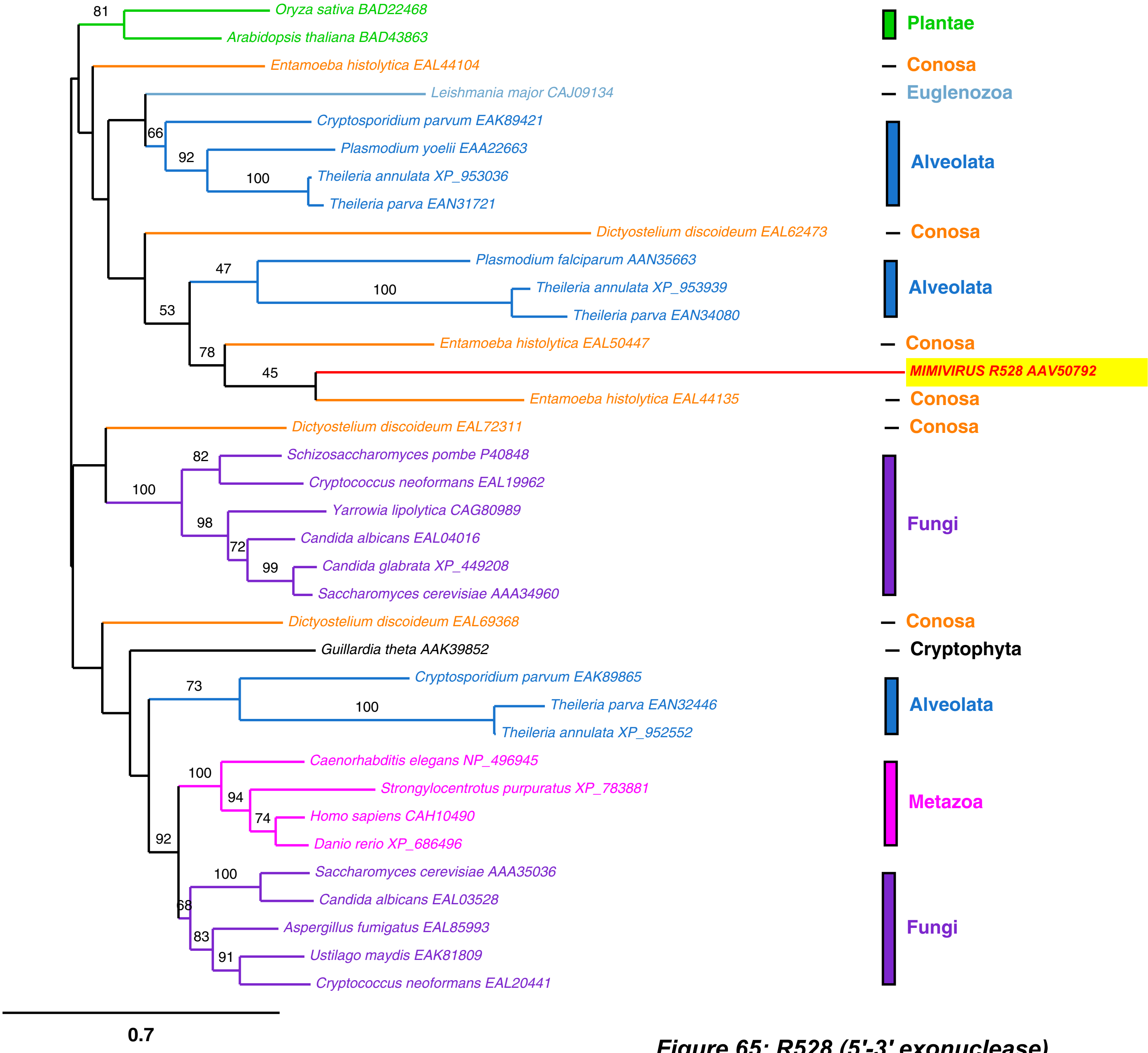

Figure 65: R528 (5'-3' exonuclease)

(36 sequences, 192 positions)

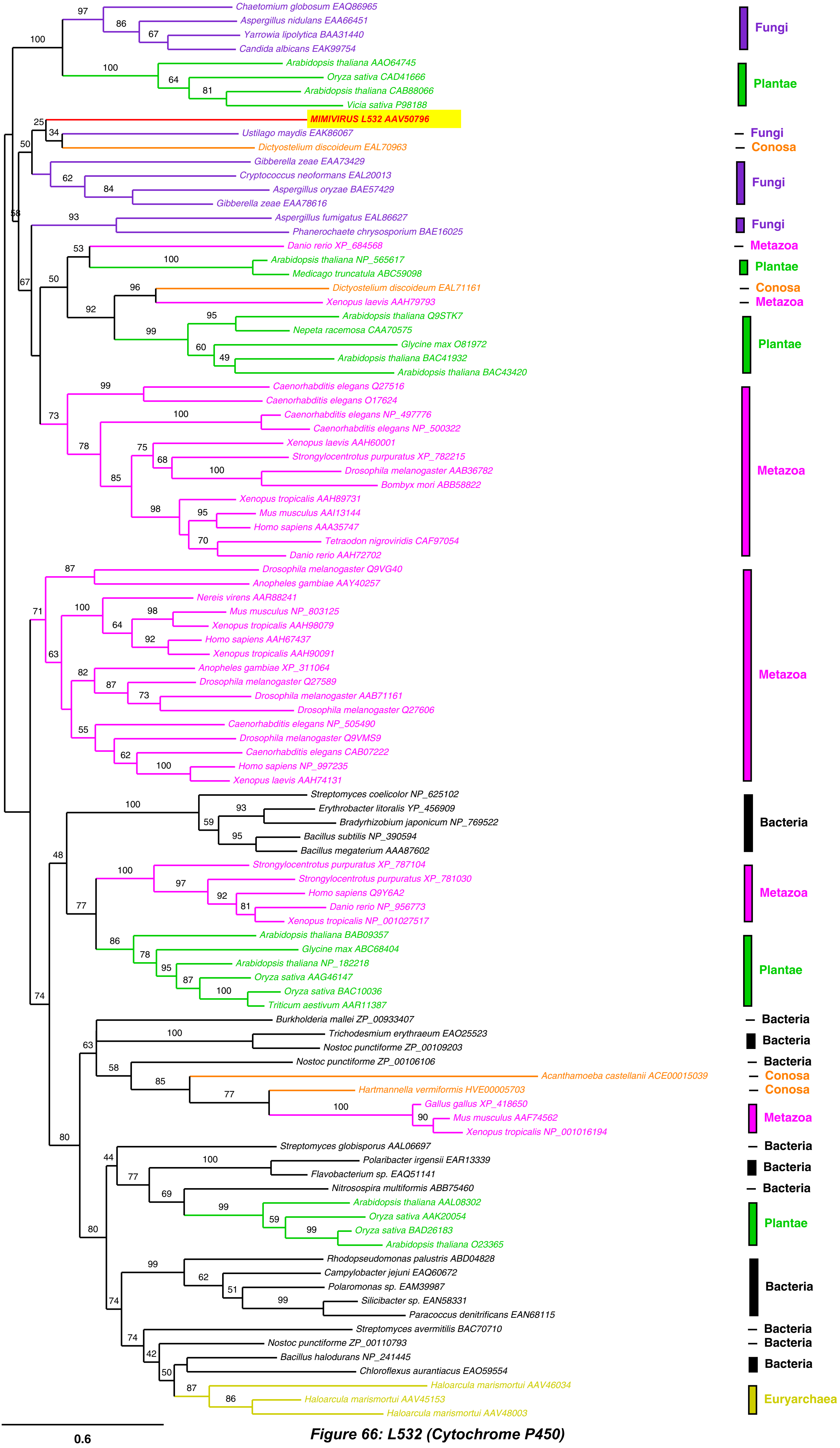

Figure 66: L532 (Cytochrome P450)
(101 sequences, 145 positions)

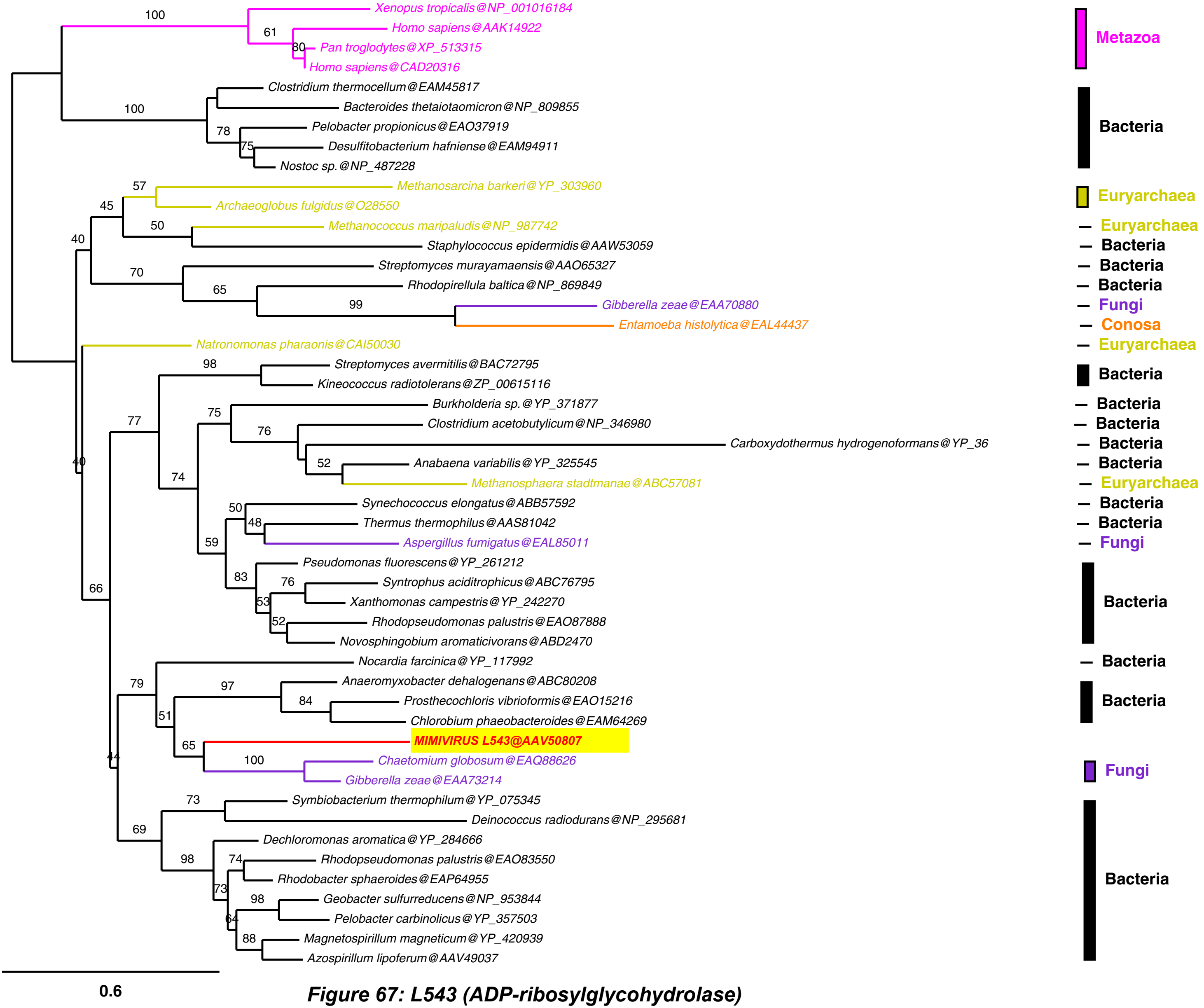

Figure 67: L543 (ADP-ribosylglycohydrolase)
(49 sequences, 79 positions)

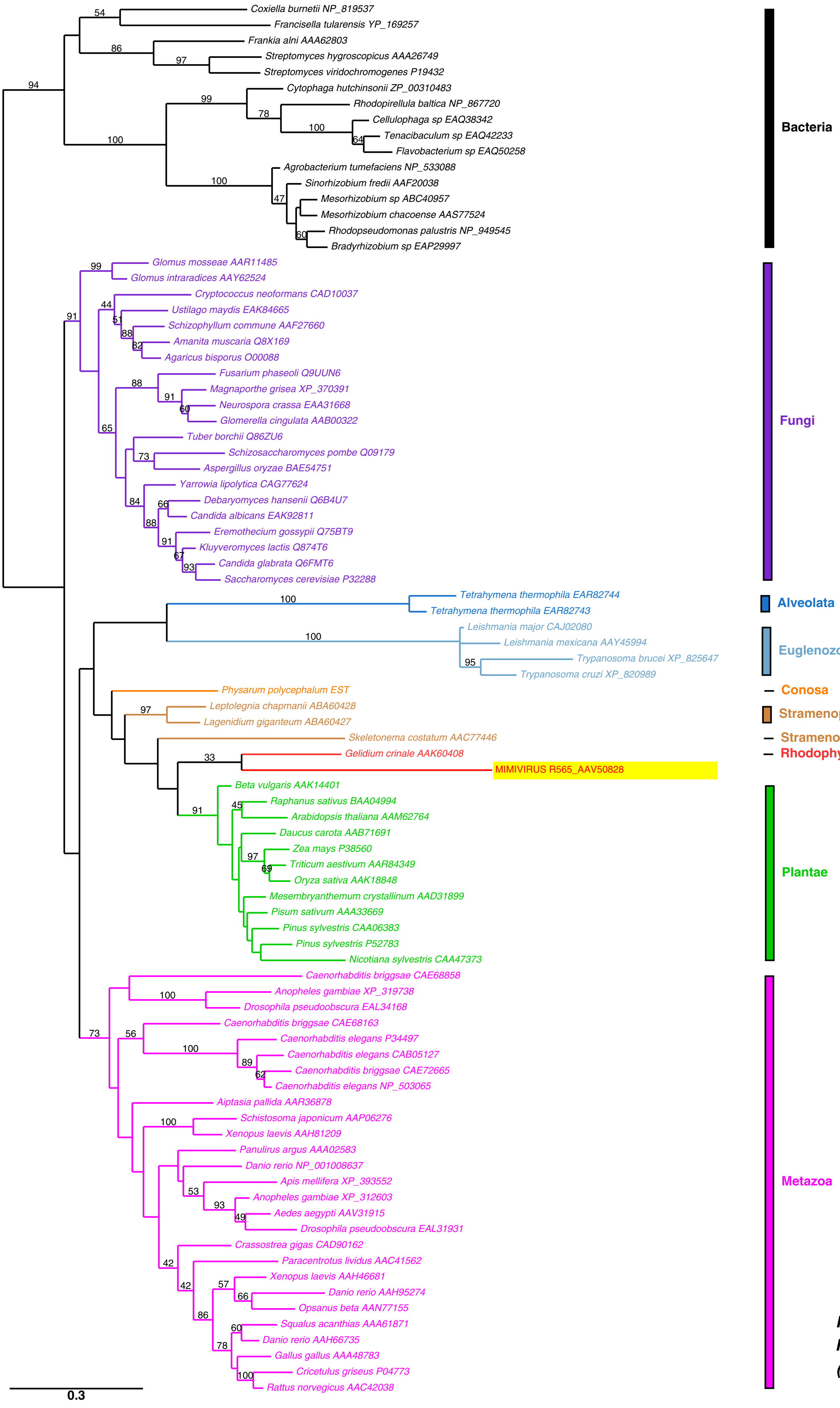

Figure 68:
R565 (Glutamine synthetase)
(88 sequences, 221 positions)

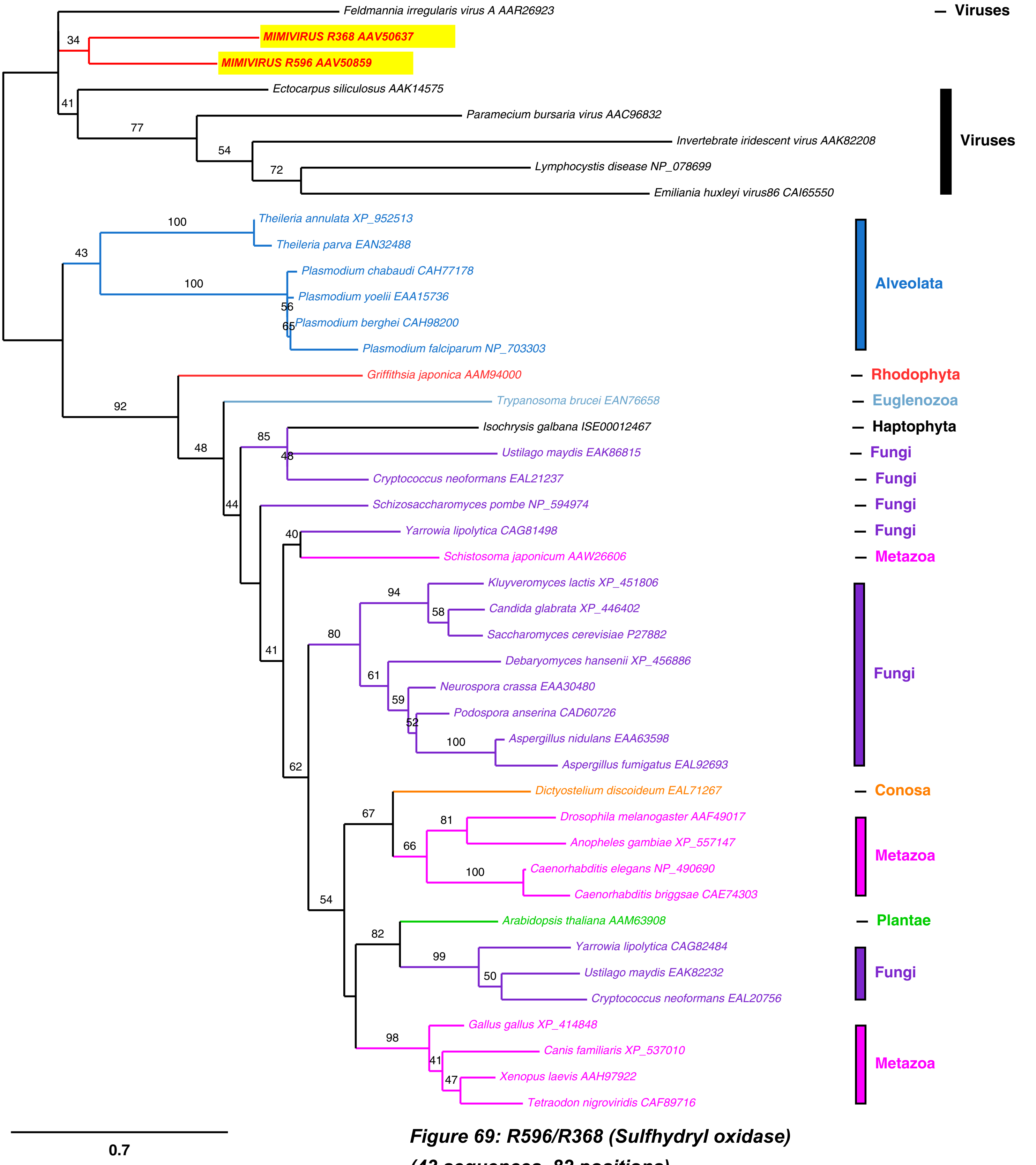

Figure 69: R596/R368 (Sulfhydryl oxidase)
(43 sequences, 82 positions)

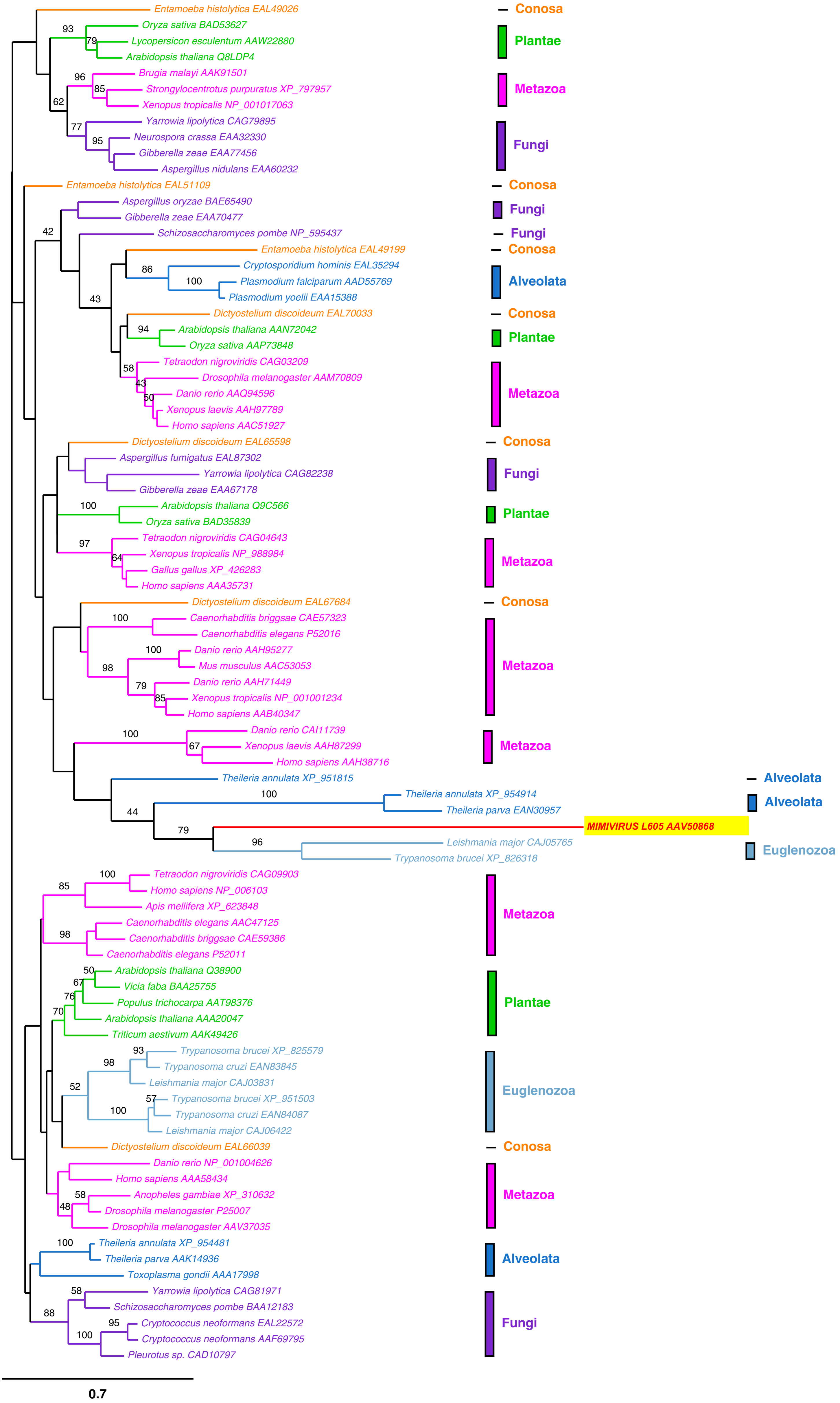

Figure 70: L605 (Peptidyl-prolyl cis-trans isomerase (rotamase) - cyclophilin family) (85 sequences, 134 positions)

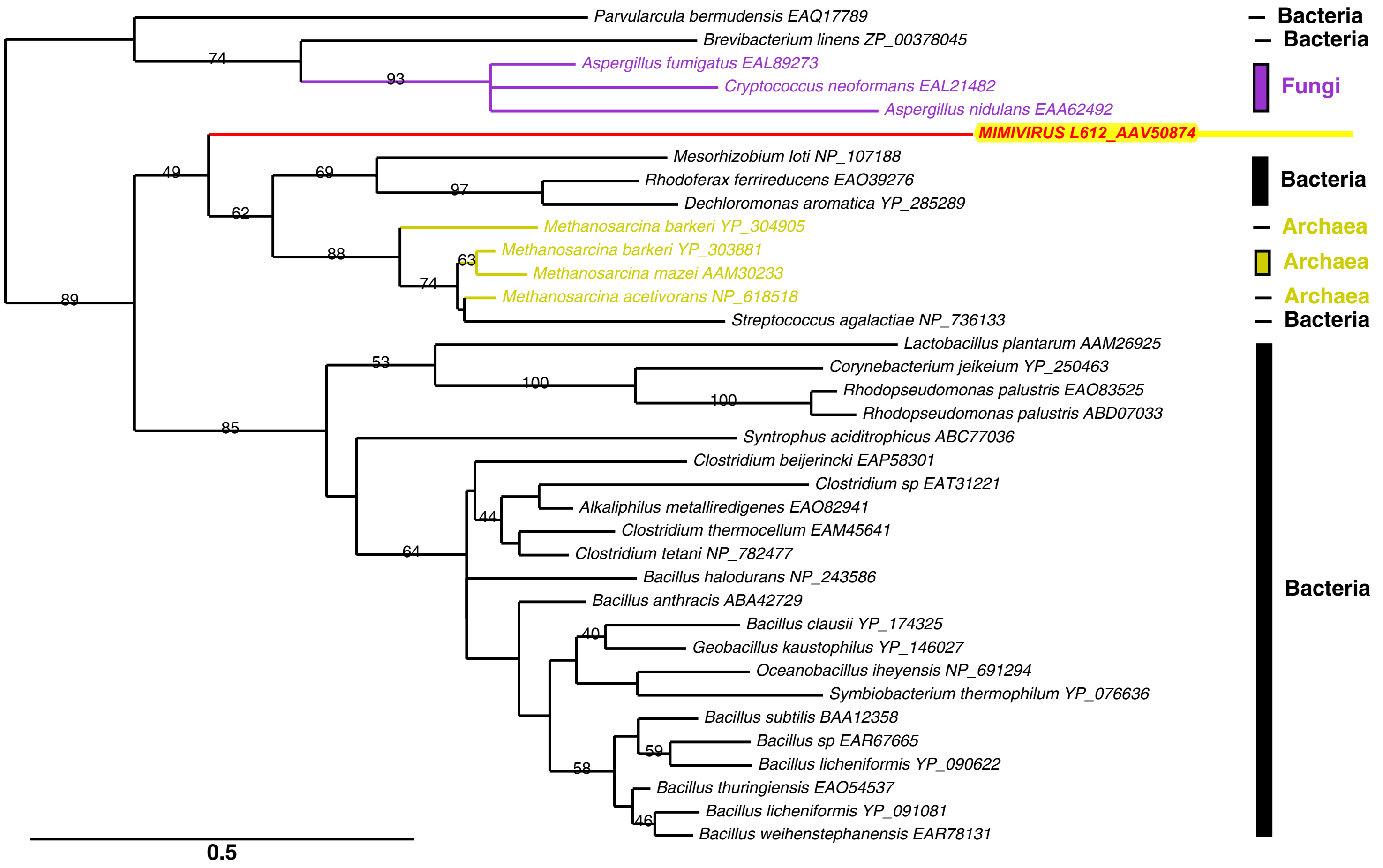

Figure 71: L612 (Mannose-6-Phosphate Isomerase)

(36 sequences, 107 positions)

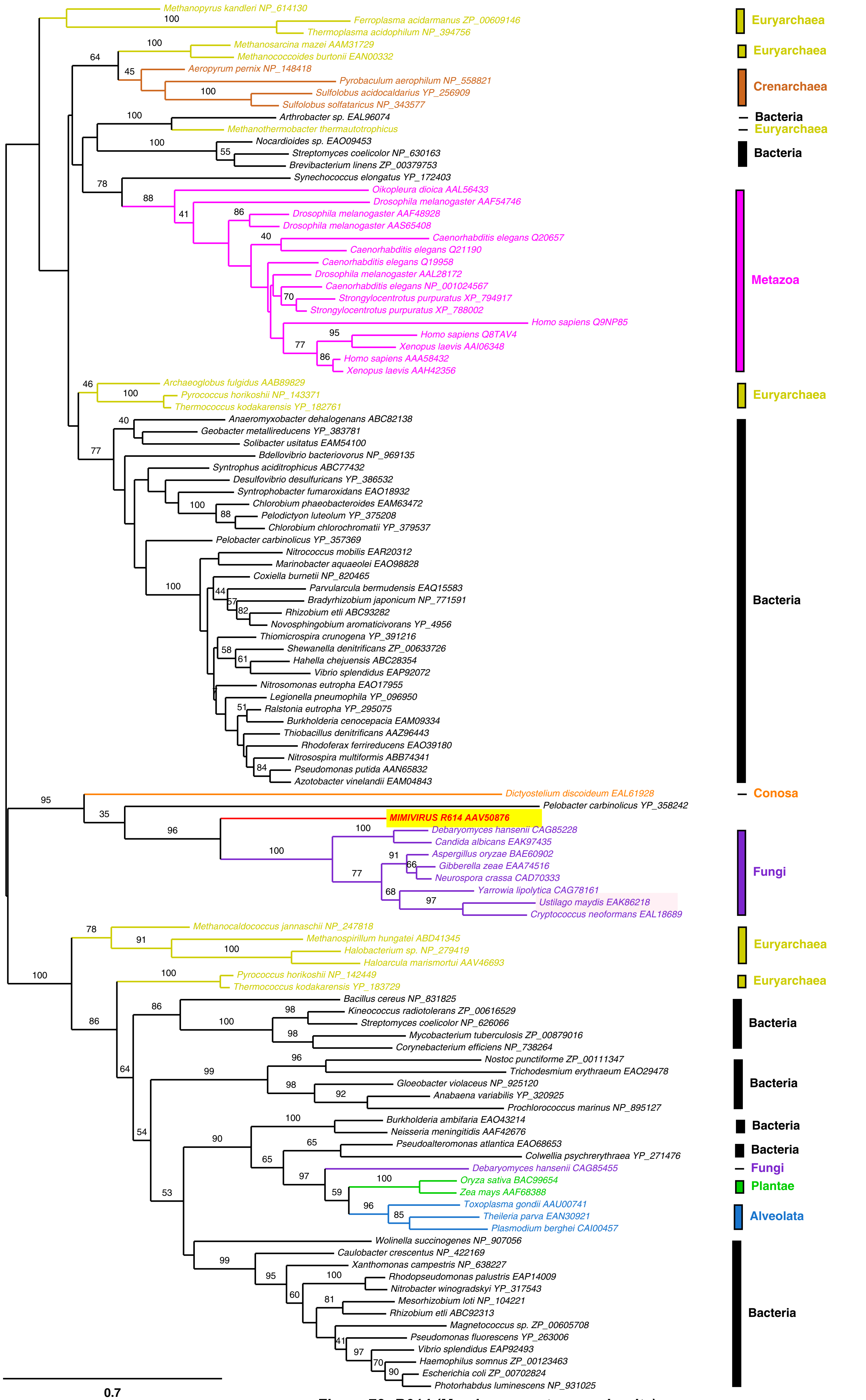

Figure 72: R614 (Membrane protease subunits)

(115 sequences, 204 positions)

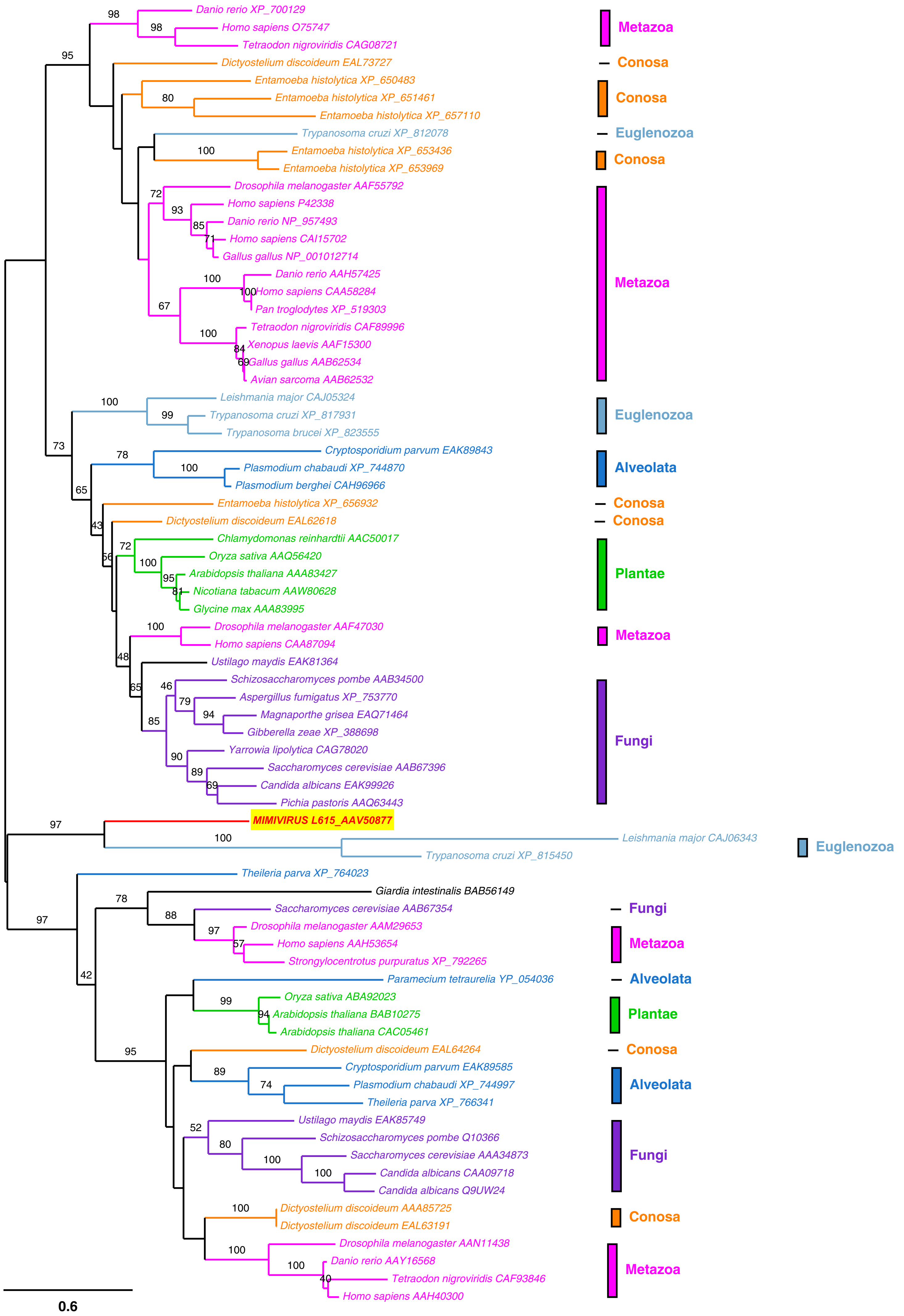

Figure 73: L615 (Phosphatidylinositol kinase and protein kinase of the PI-3 kinase family) (74 sequences, 175 positions)

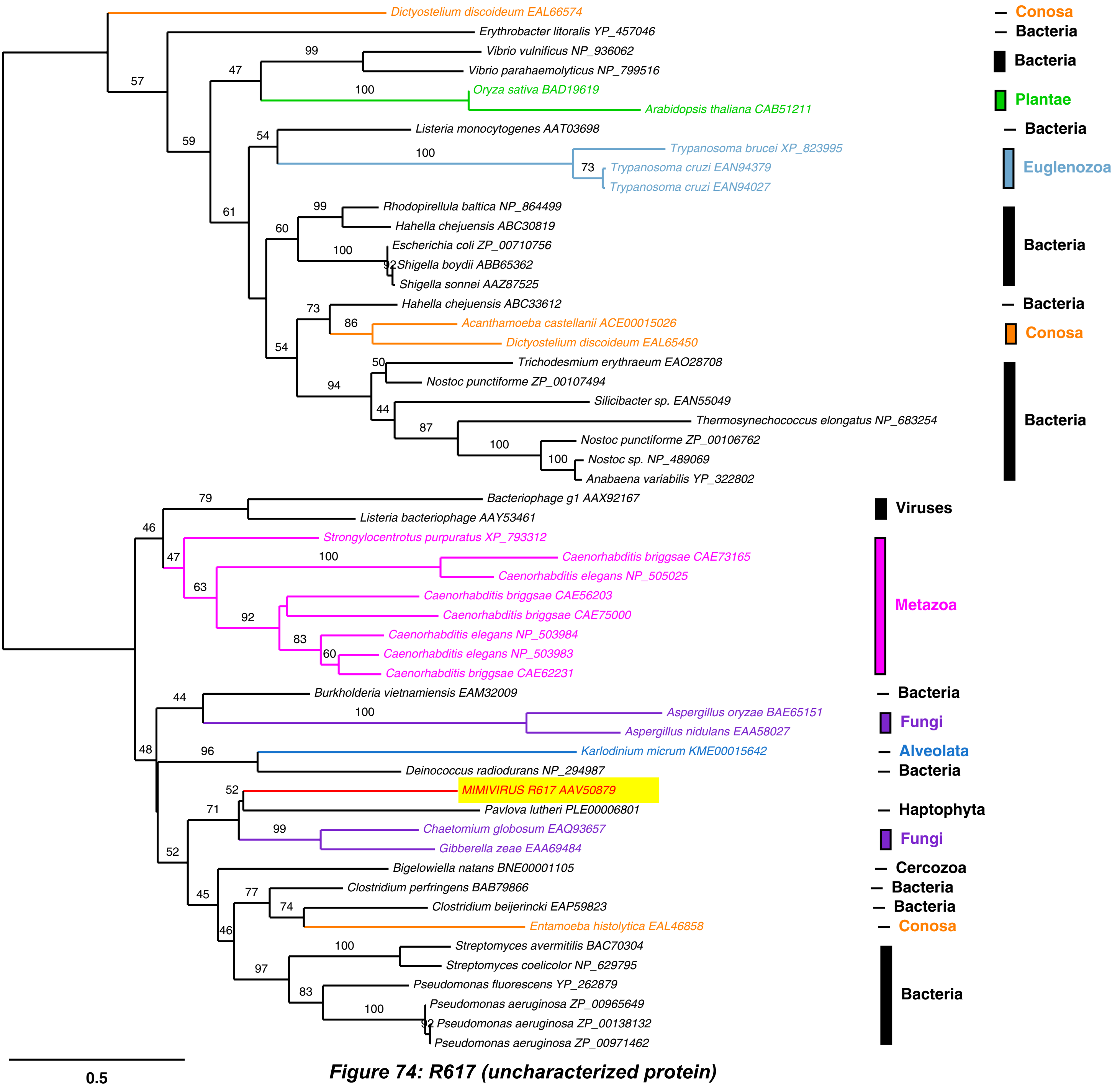

Figure 74: R617 (uncharacterized protein)

(54 sequences, 133 positions)

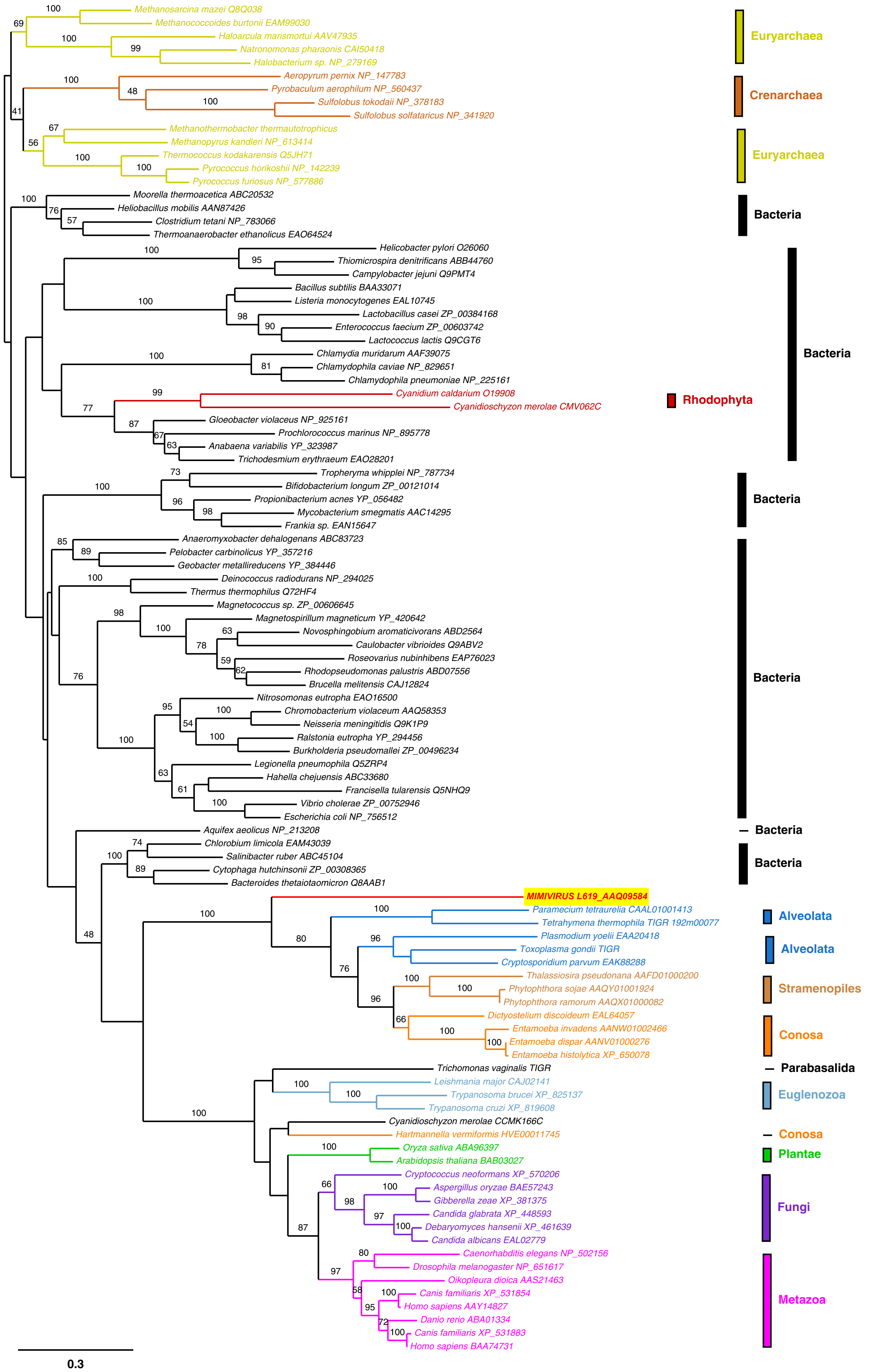

Figure 75: L619 (Glucosamine 6-phosphate synthetase)

(102 sequences, 407 positions)

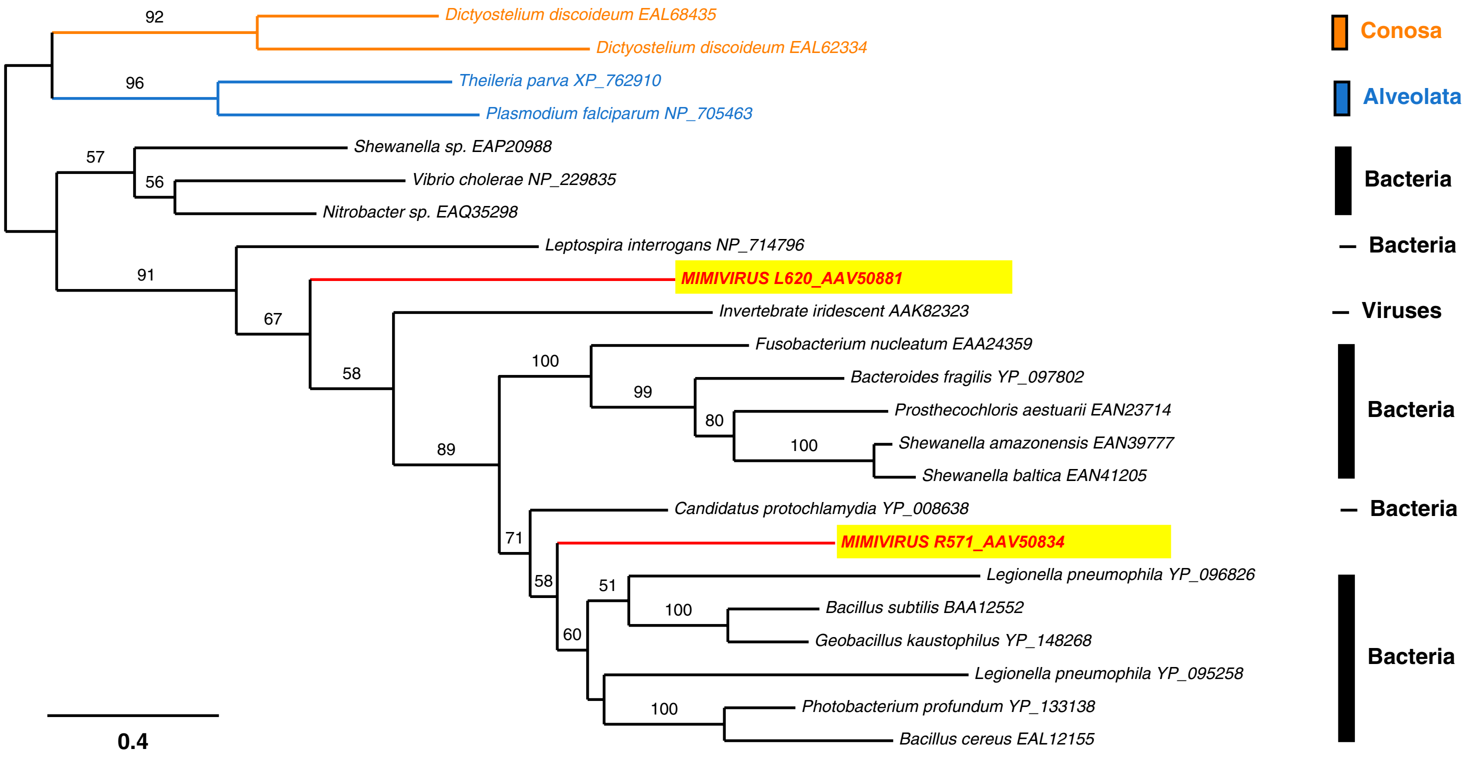

Figure 76: L620/R571 (Patatin)

(23 sequences, 107 positions)

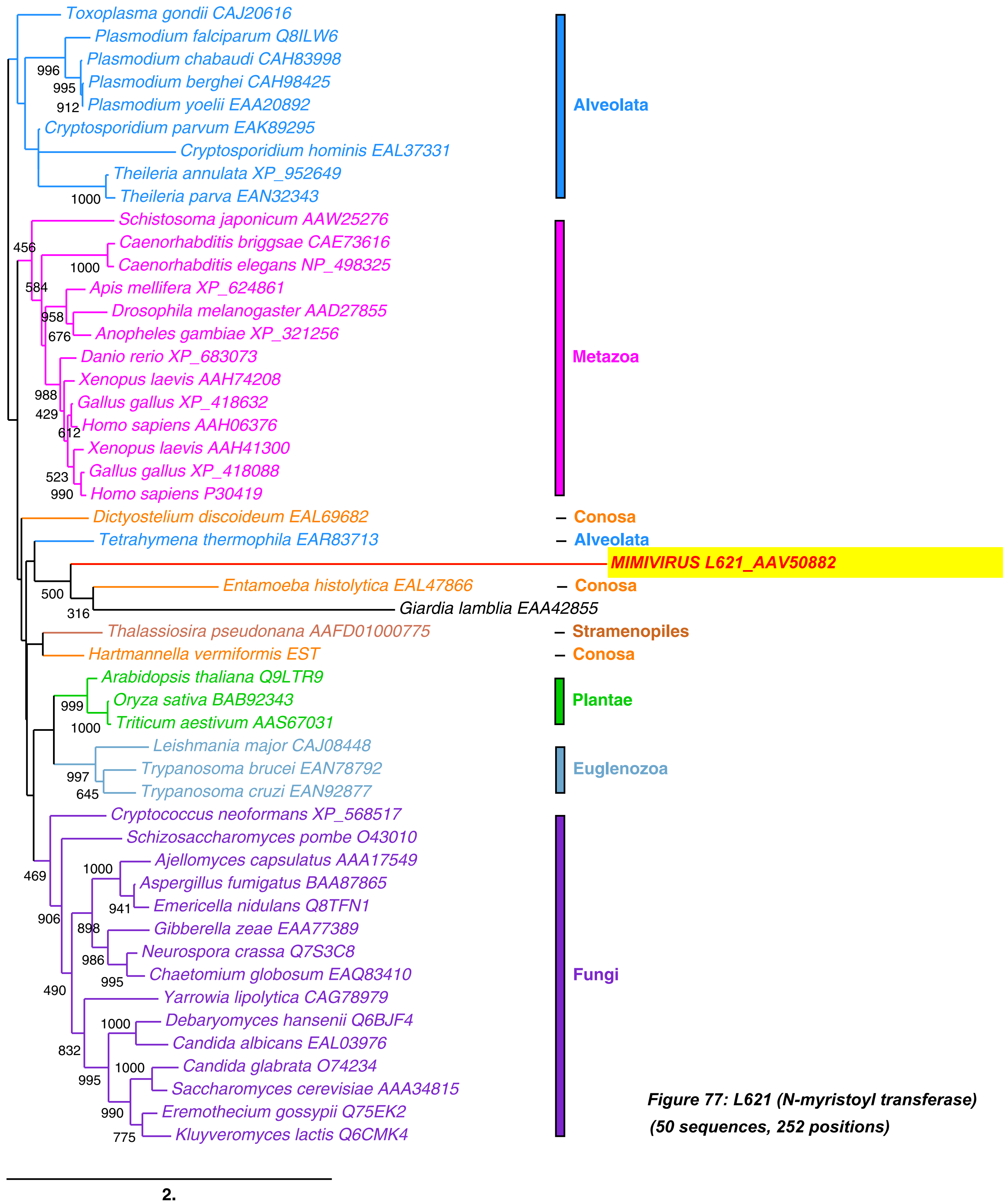

Figure 77: L621 (N-myristoyl transferase)
(50 sequences, 252 positions)

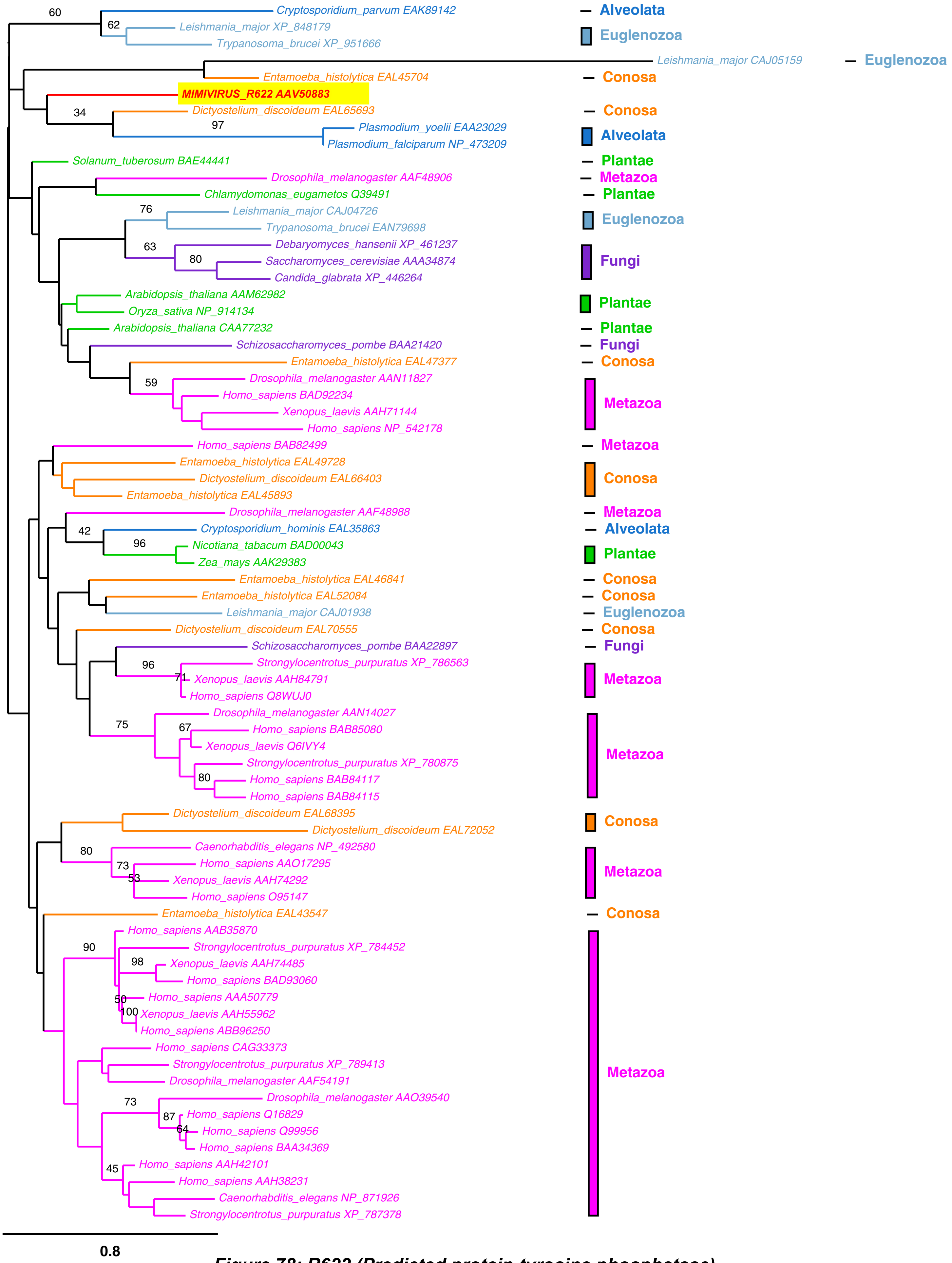

Figure 78: R622 (Predicted protein-tyrosine phosphatase)
(73 sequences, 61 positions)

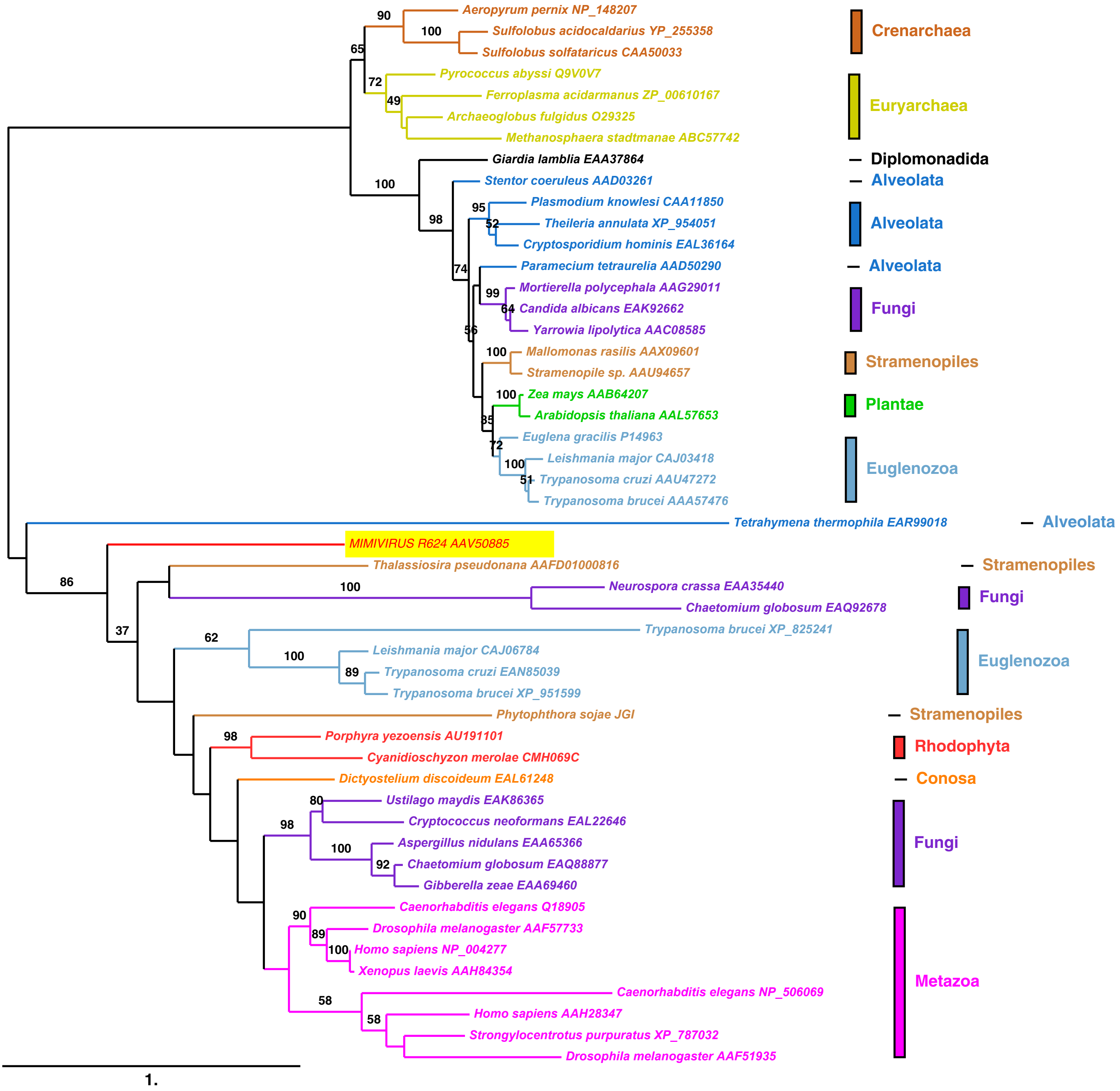

Figure 79: R624 (GTPase)

(50 sequences, 243 positons)

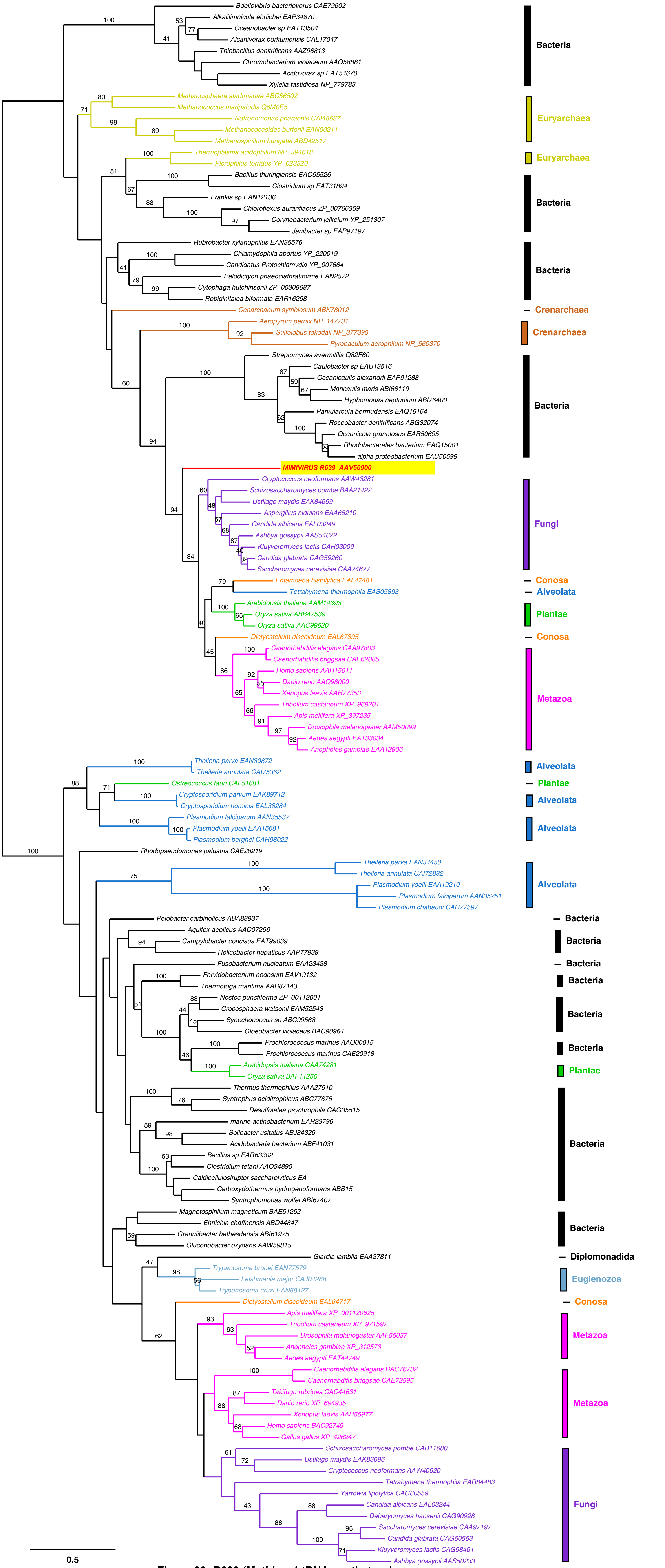

Figure 80: R639 (Methionyl-tRNA synthetase)
(139 sequences, 212 positions)

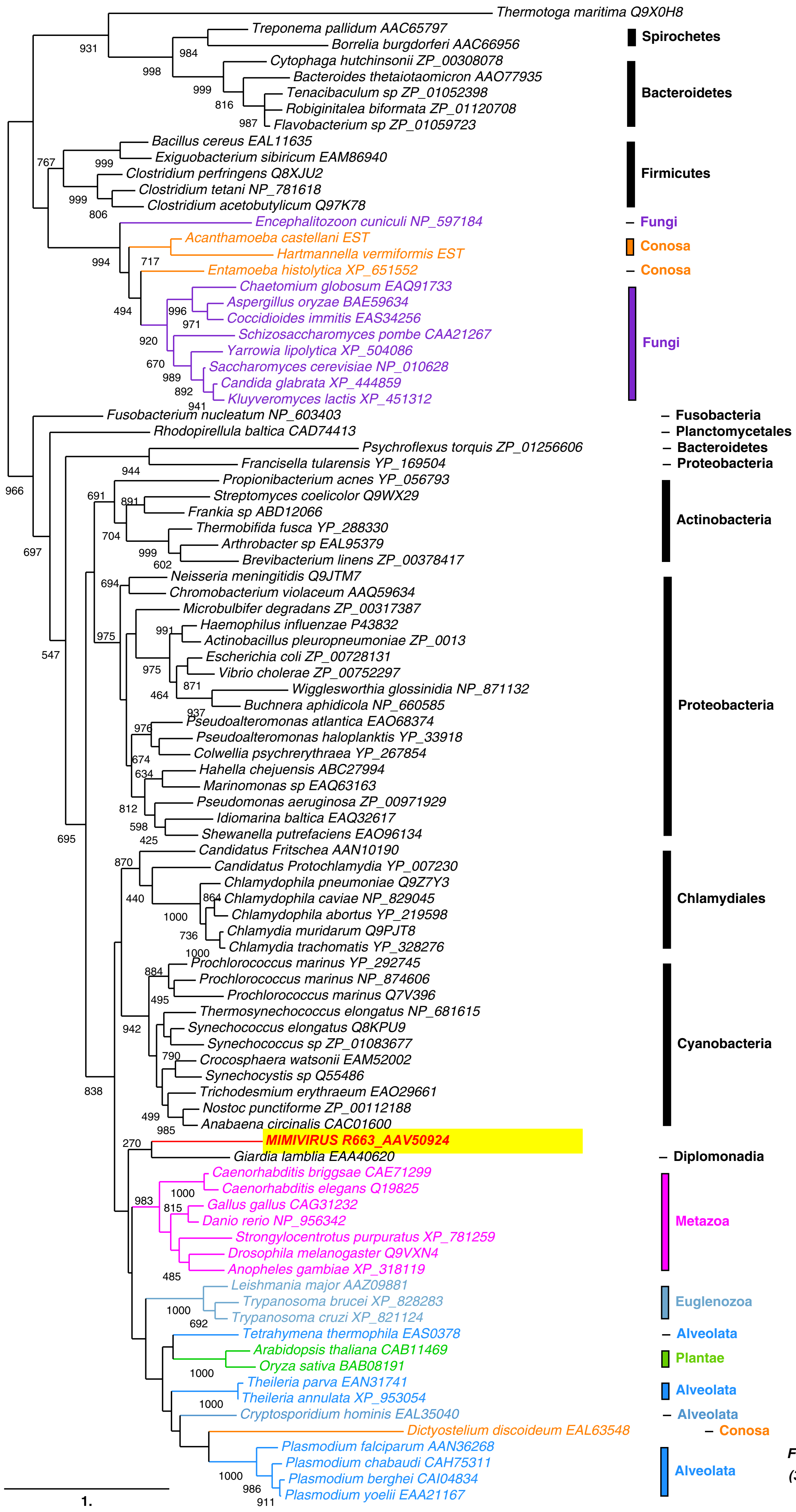

Figure 81: R663 (Arginyl-tRNA synthetase)
(39 sequences, 236 positions)

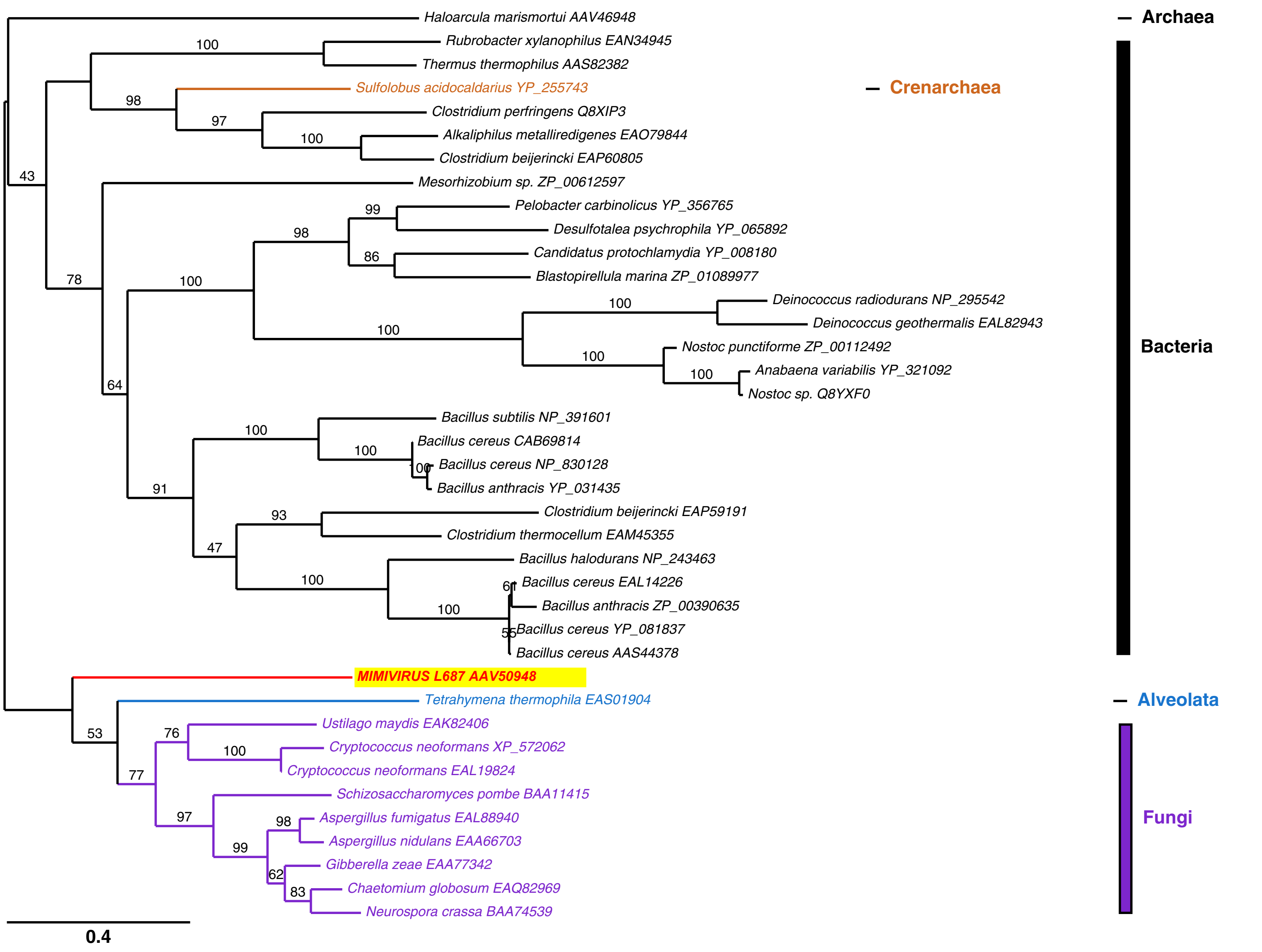

Figure 82: L687 (UV damage repair endonuclease)

(39 sequences, 221 positions)

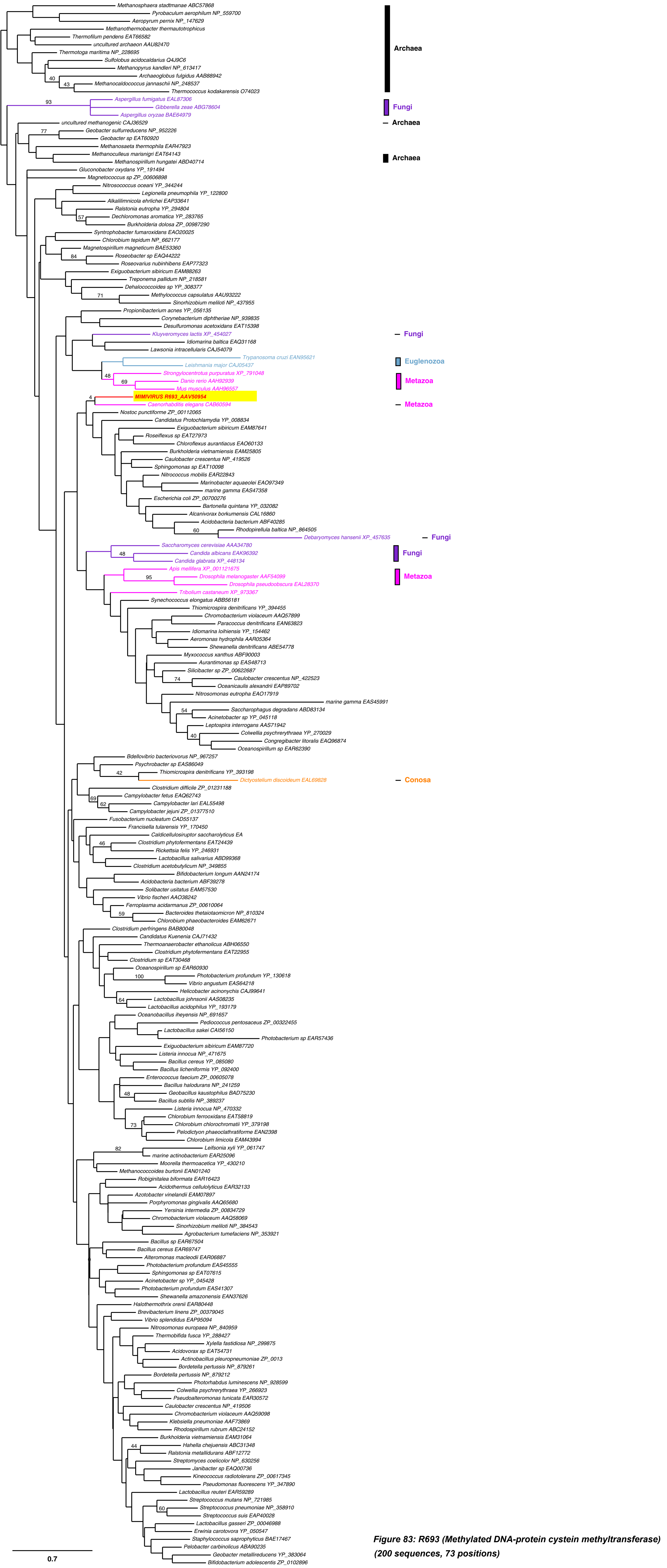

Figure 83: R693 (Methylated DNA-protein cystein methyltransferase) (200 sequences, 73 positions)

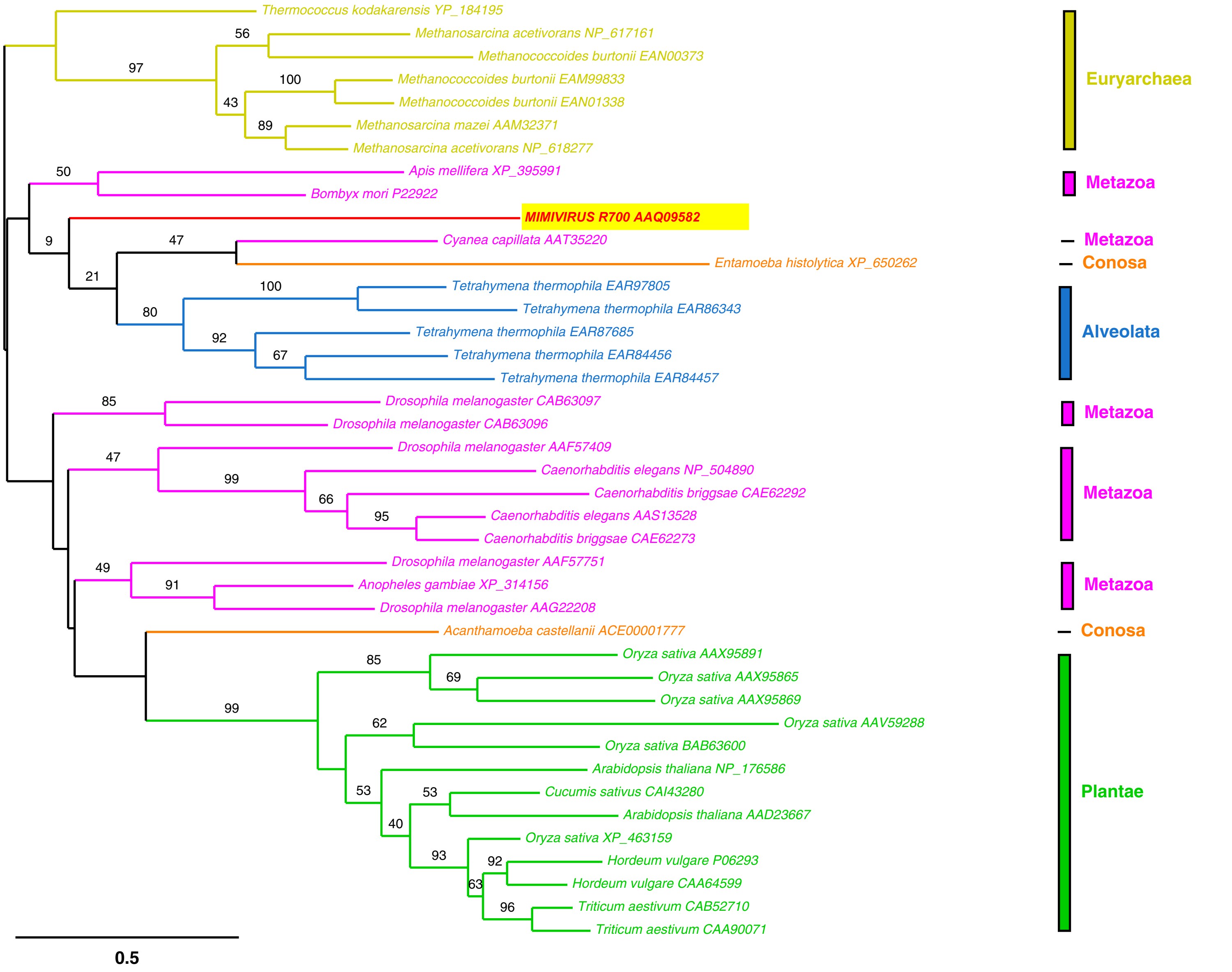

Figure 84: R700 (Serine protease inhibitor)
(41 sequences, 165 postions)

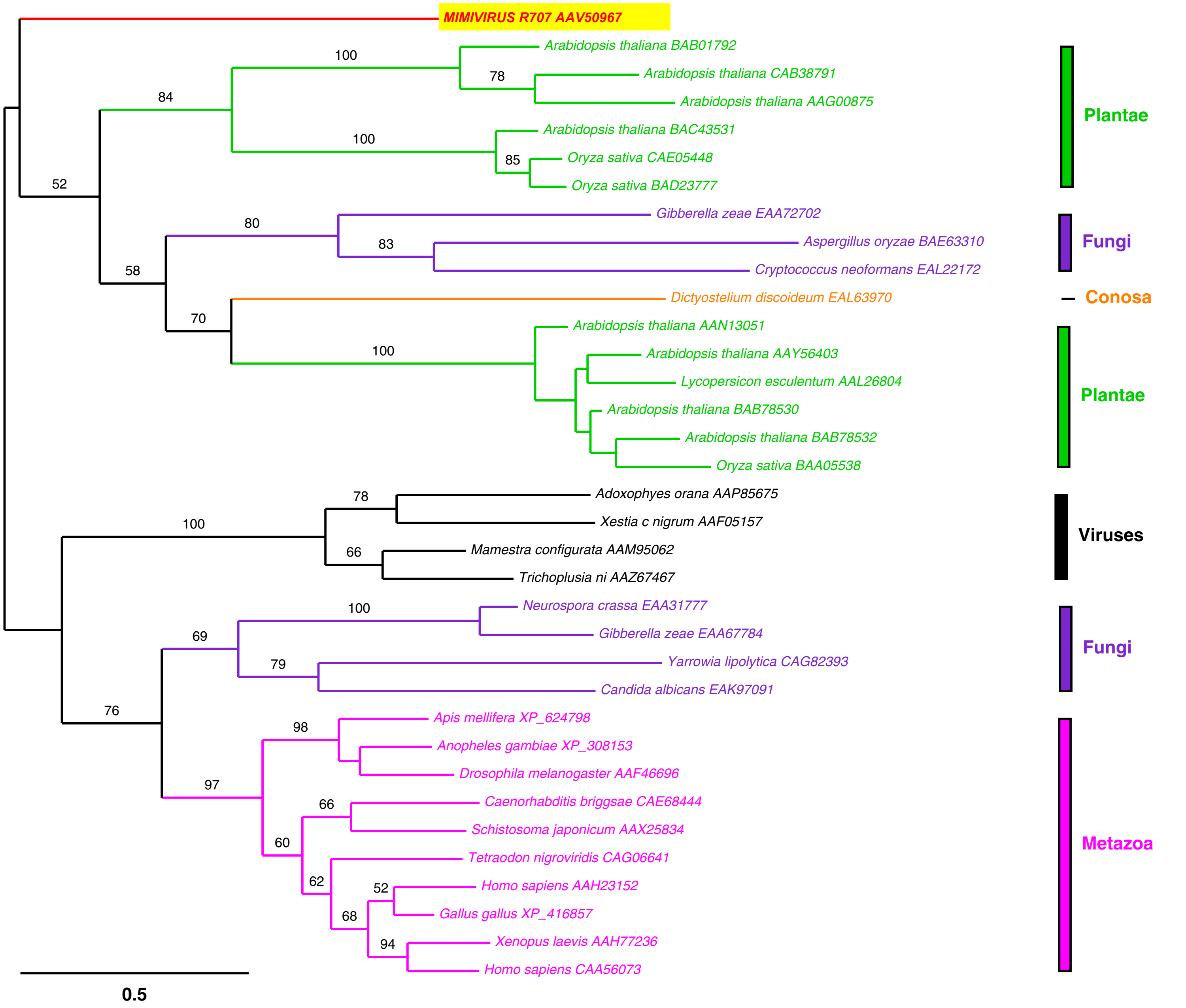

Figure 85: R707 (Alpha-N-acetylglucosamine transferase)
(35 sequences, 133 positions)

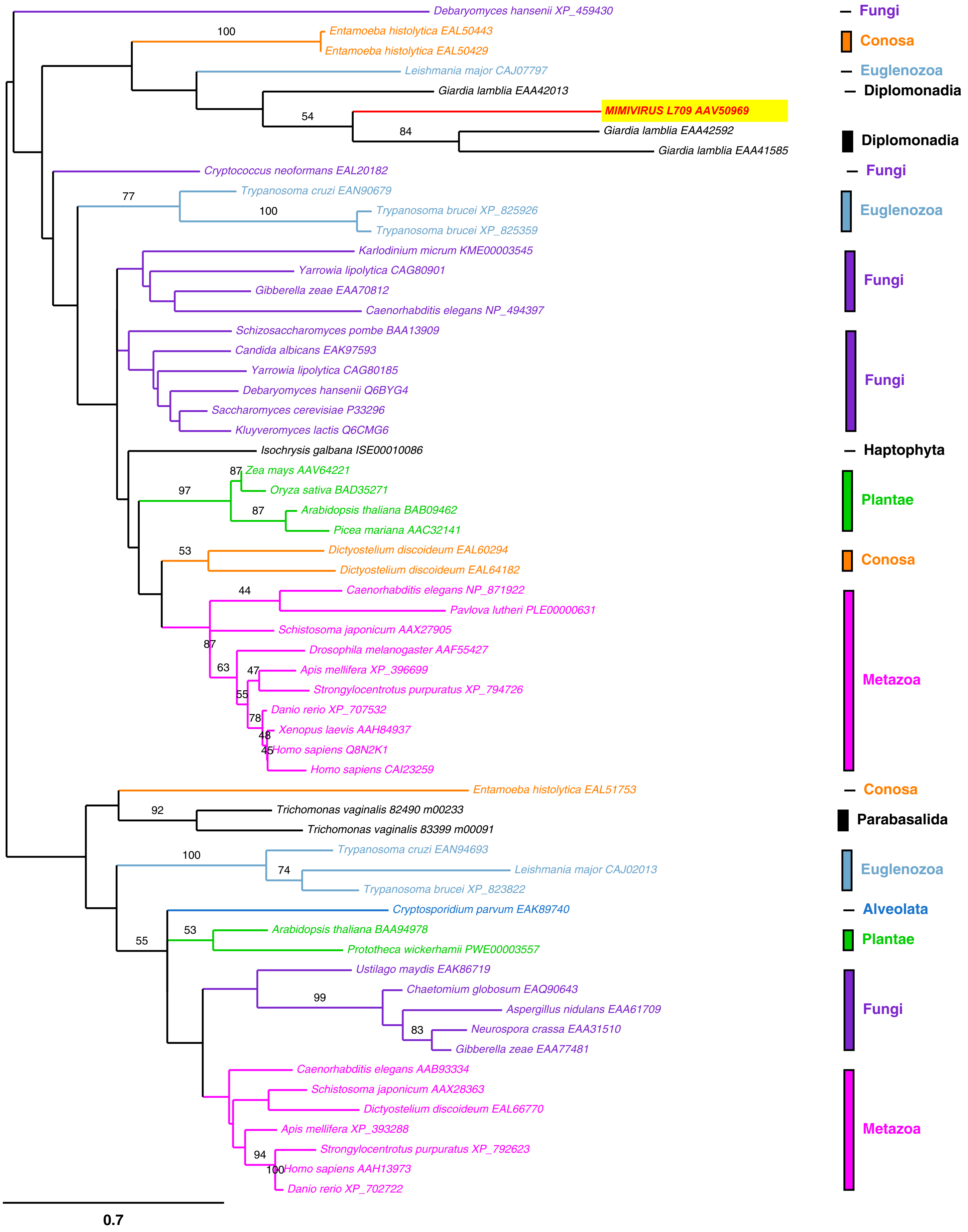

Figure 86: L709 (Ubiquitin-protein ligase)
(60 sequences, 100 positions)

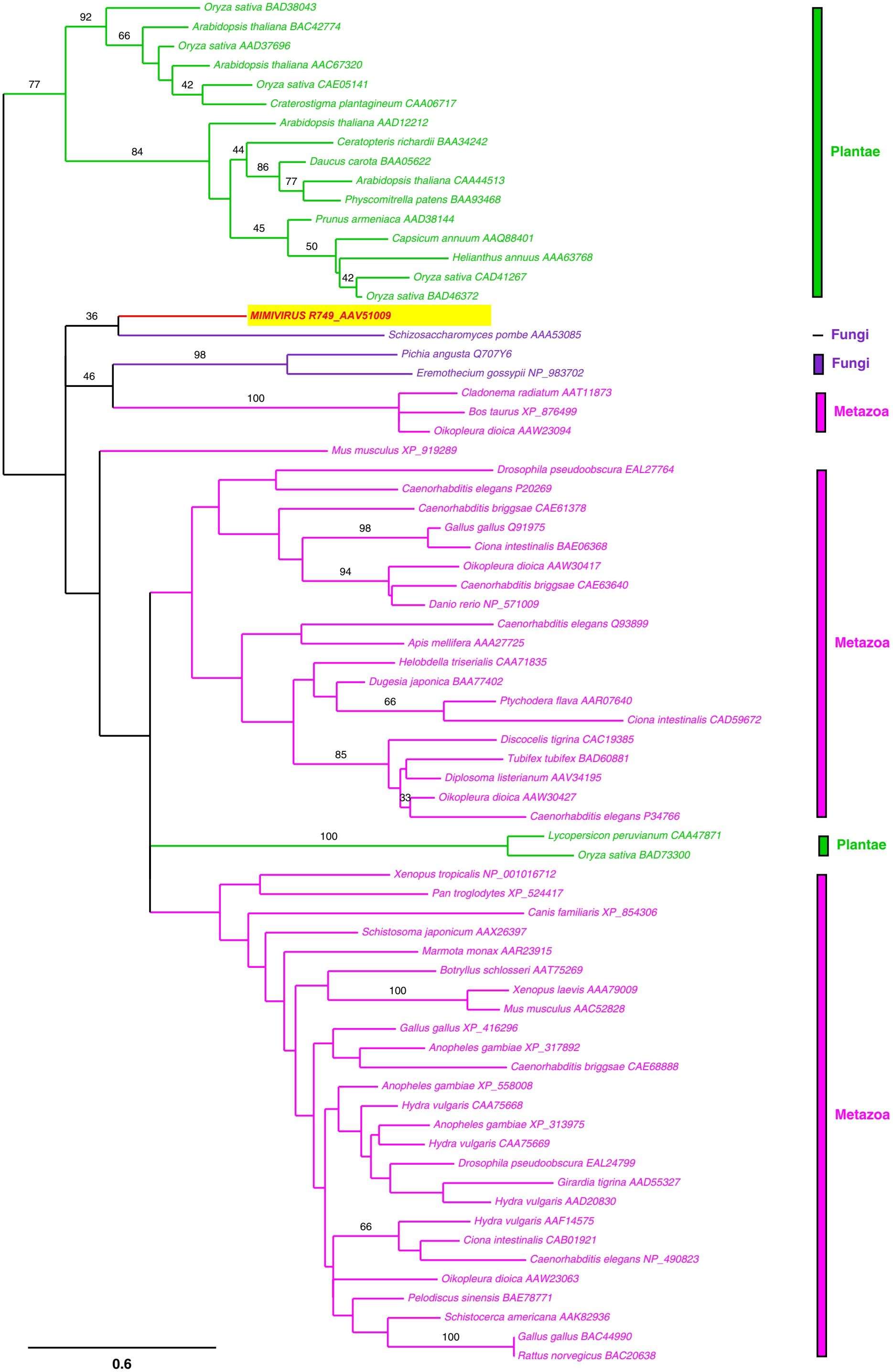

Figure 87: R749 (Homeodomain-containing transcription factor) (71 sequences, 54 positions)

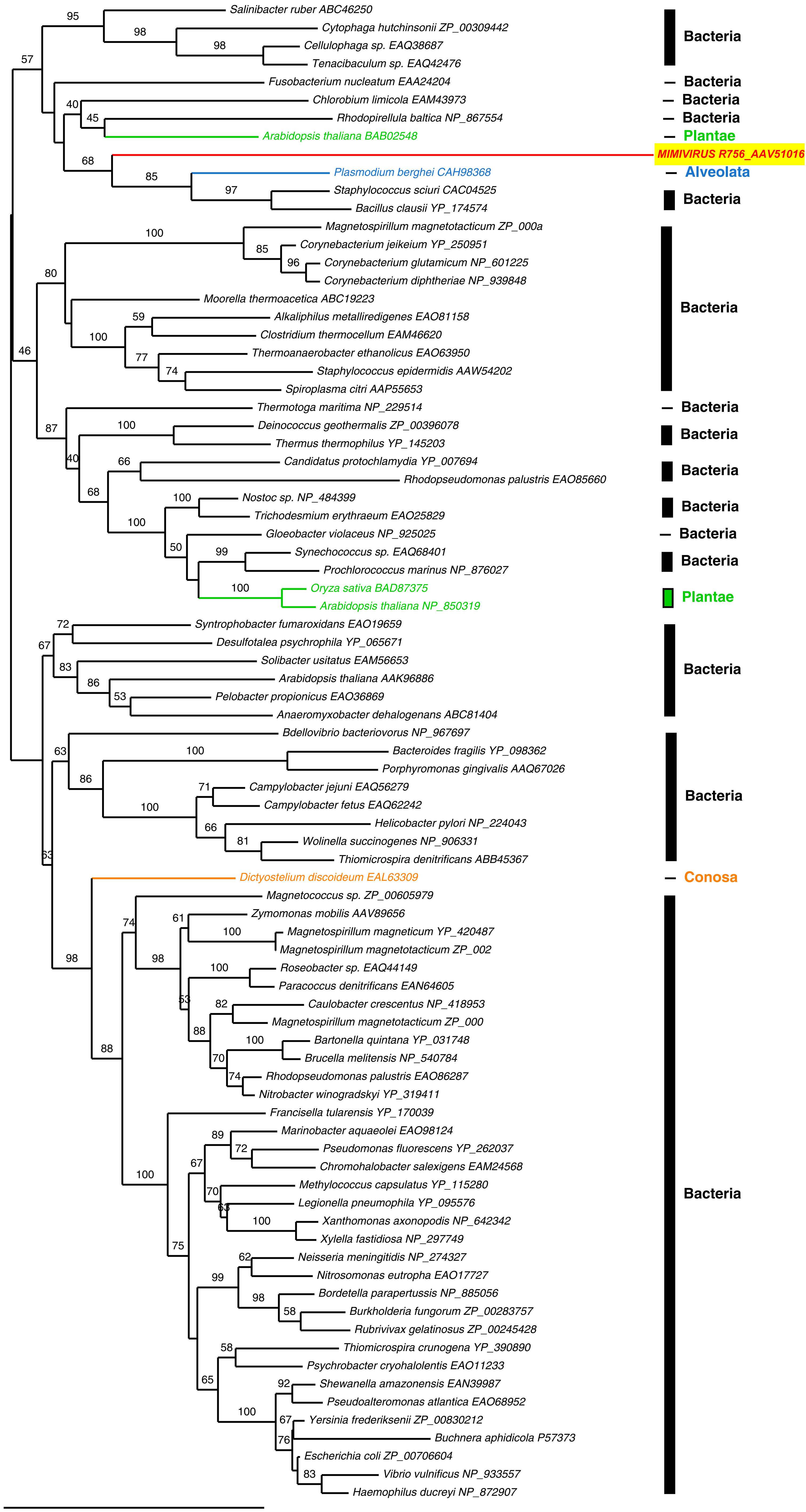

Figure 88: R756 (Predicted De-S cluster redox enzyme) (83 sequences, 196 positions)

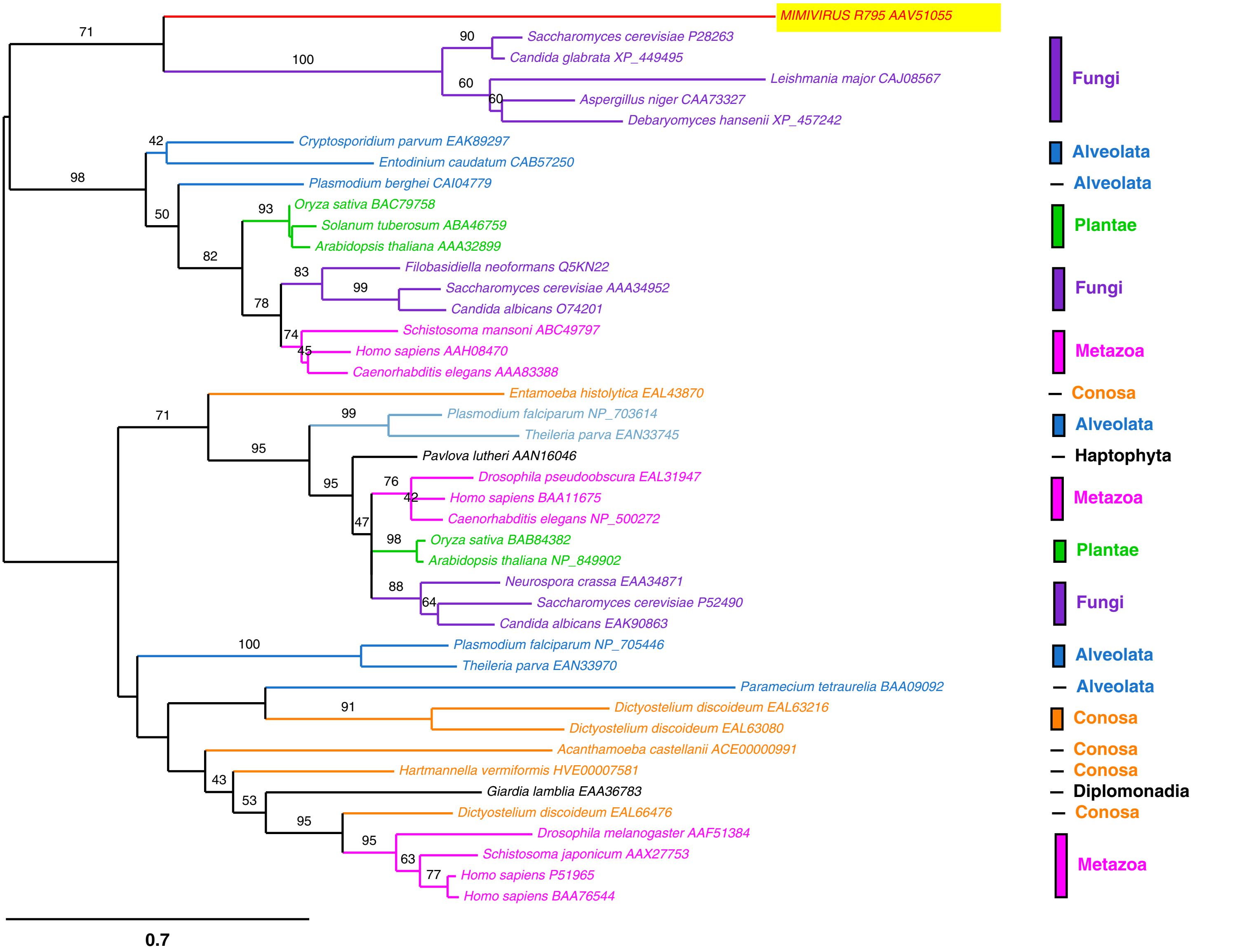

Figure 89: R795 (Ubiquitin-protein ligase)

(43 sequences, 143 positions)

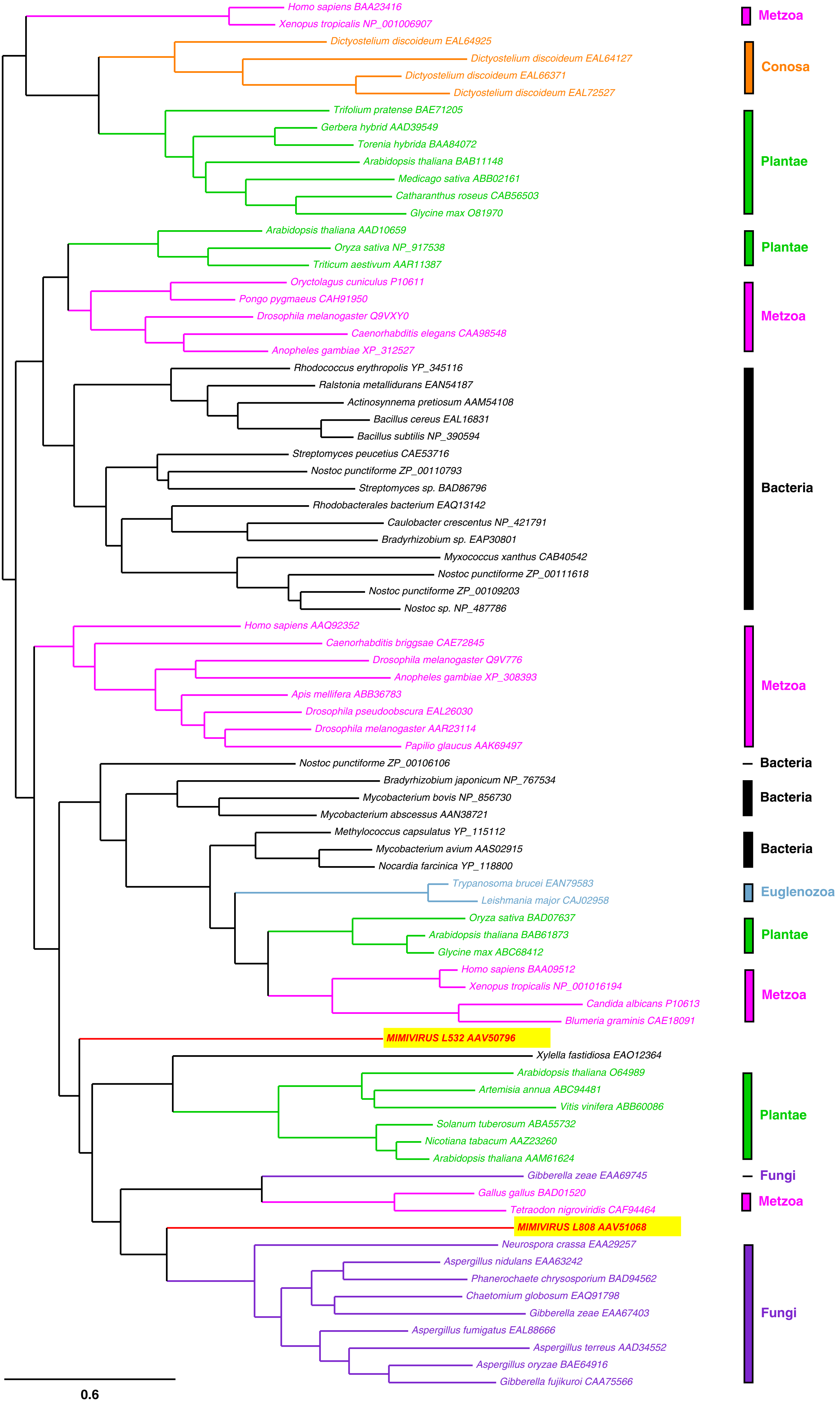

Figure 90: L808 (lanosterol 14-alpha-demethylase)
(81 sequences, 140 positions)

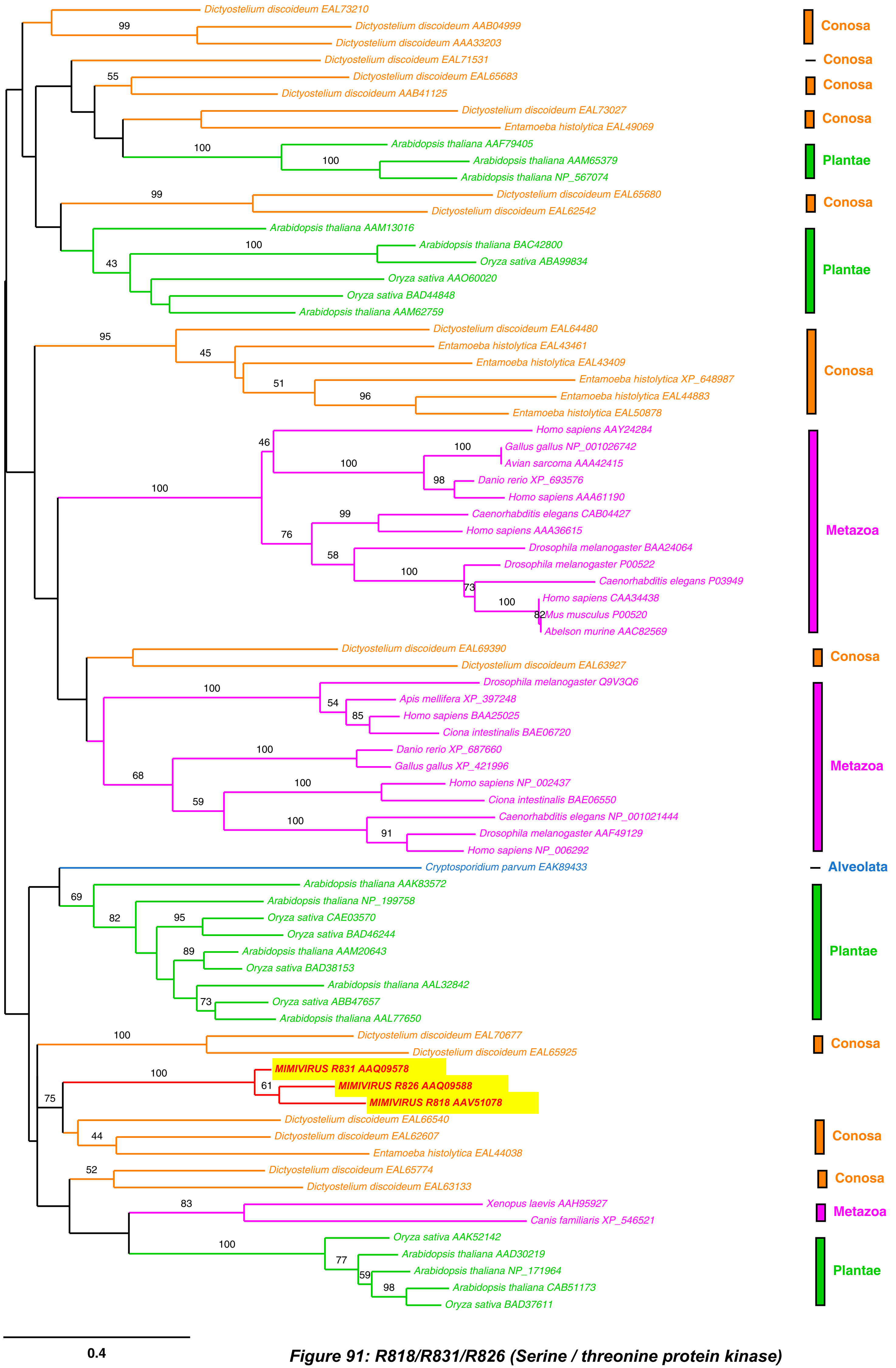

Figure 91: R818/R831/R826 (Serine / threonine protein kinase) (78 sequences, 192 positions)

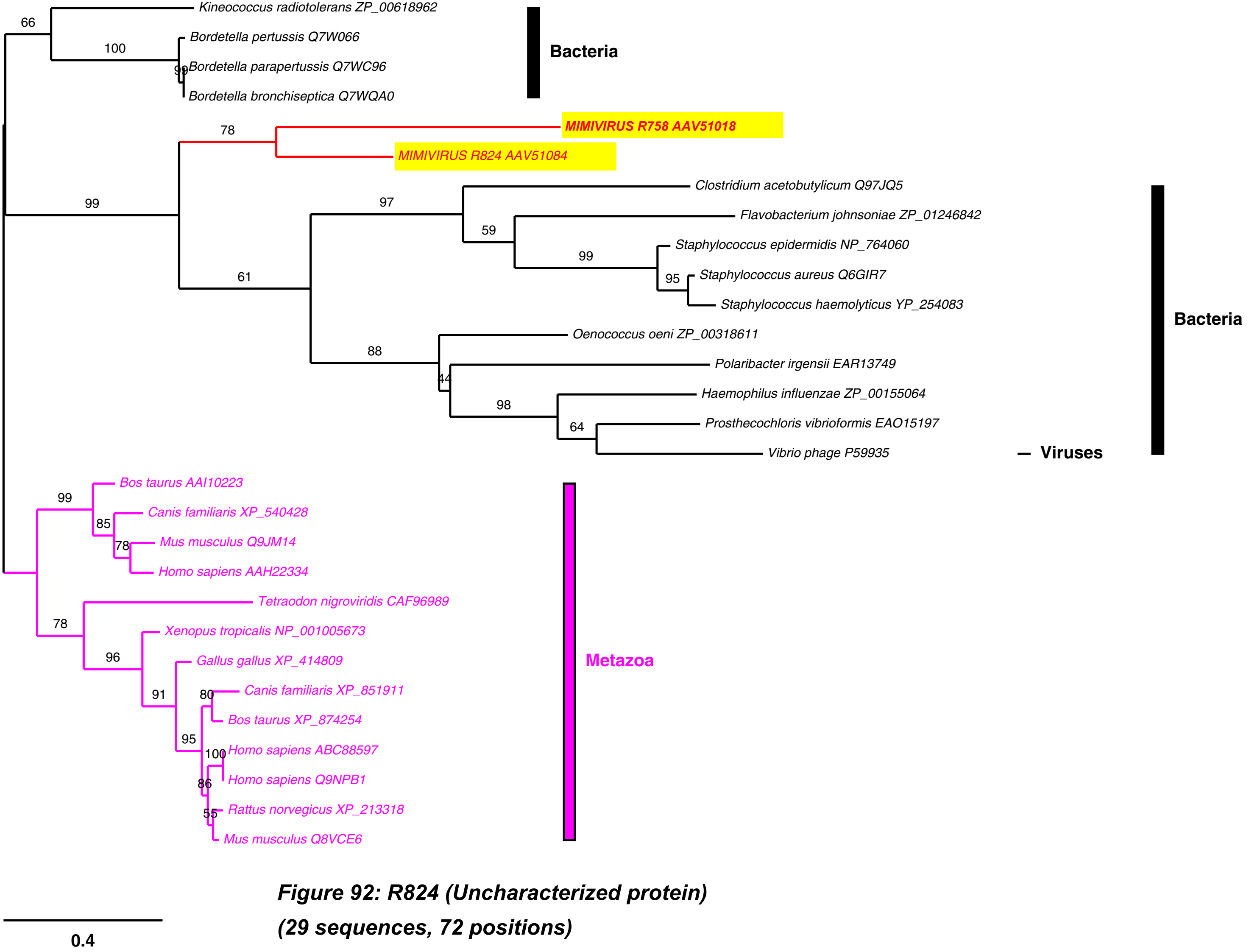

Figure 92: R824 (Uncharacterized protein)
(29 sequences, 72 positions)

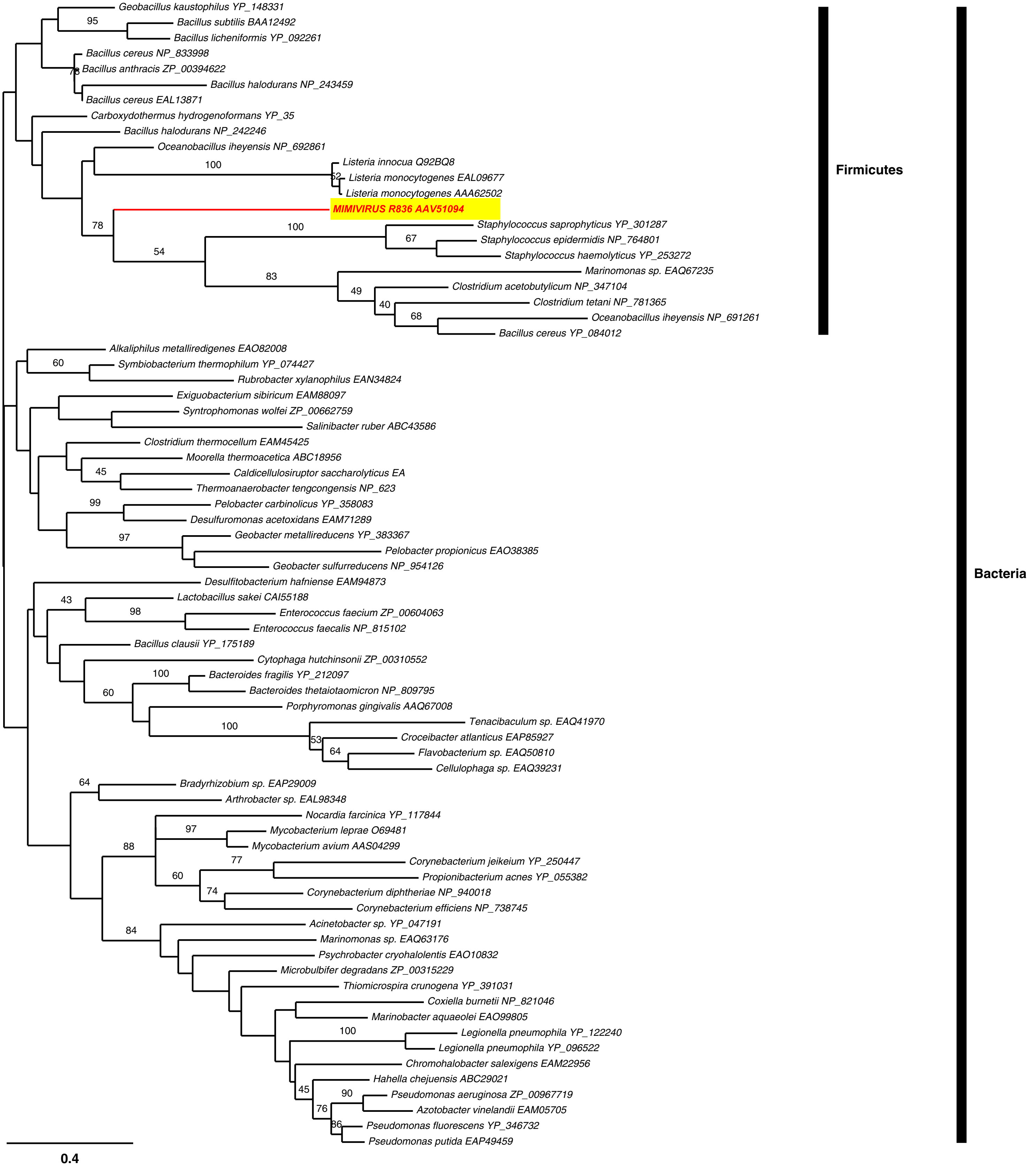

Figure 93: R836 (Uncharacterized protein)

(74 sequences, 100 positions)

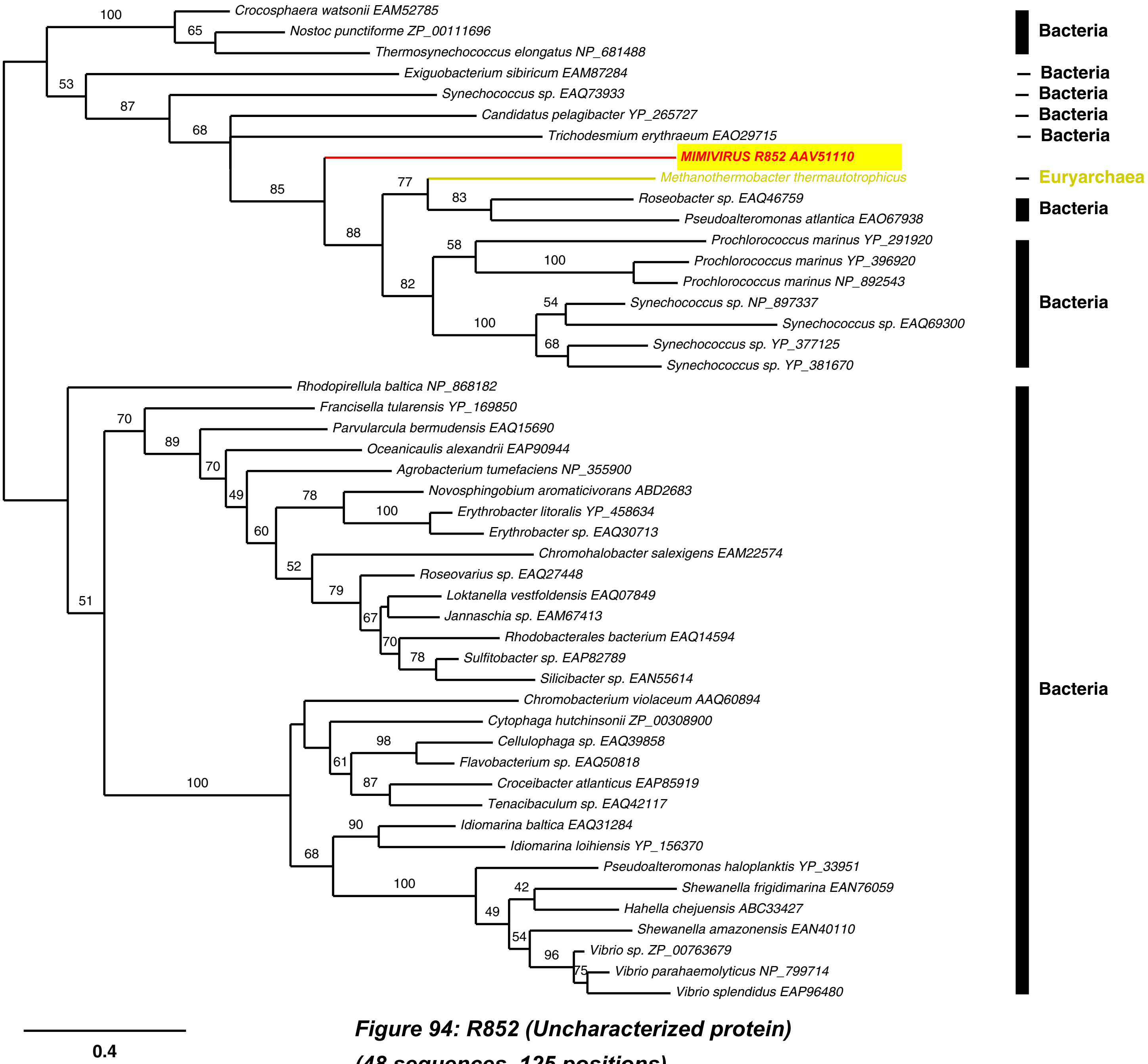

Figure 94: R852 (Uncharacterized protein)

(48 sequences, 125 positions)

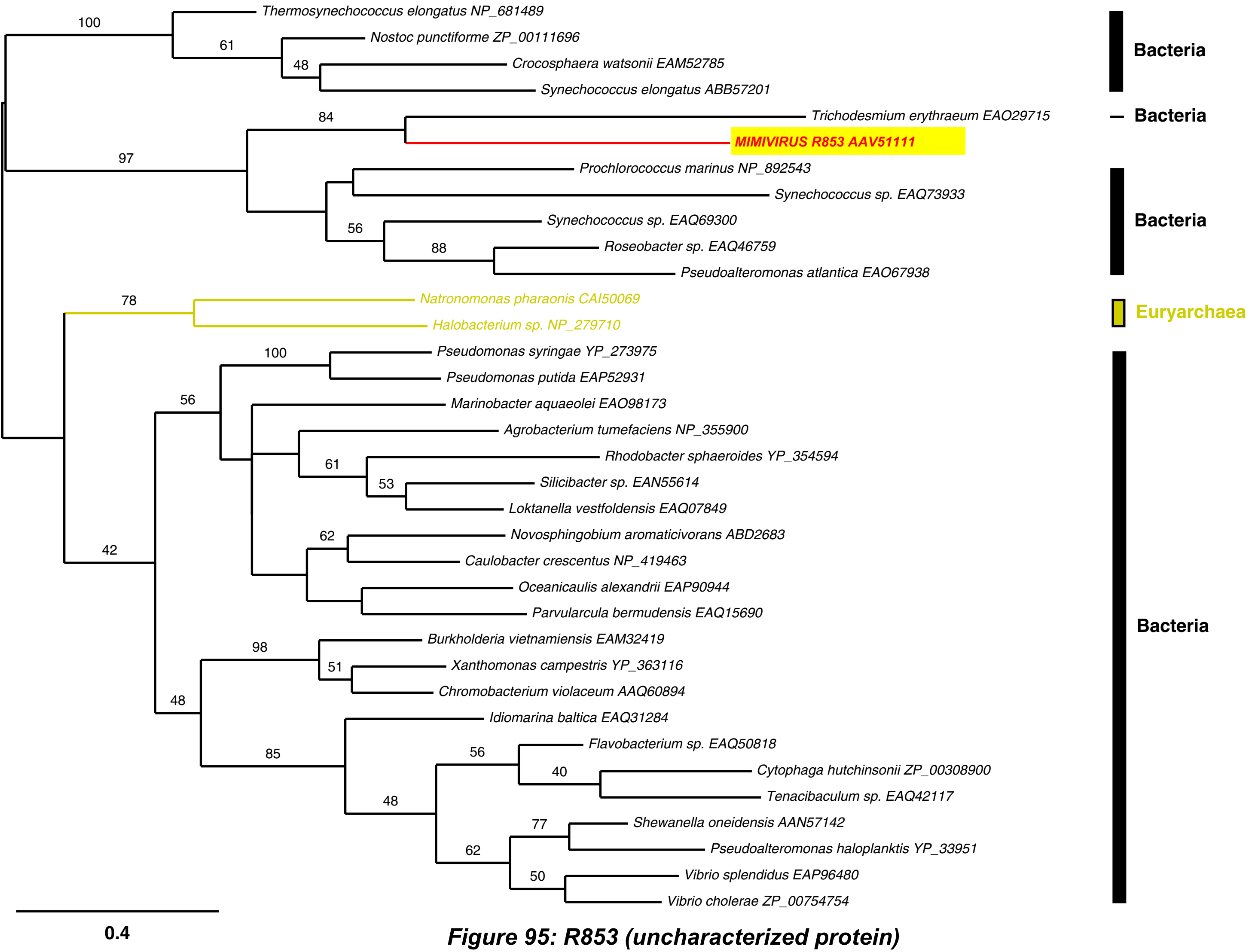

Figure 95: R853 (uncharacterized protein)

(35 sequences, 100 positions)

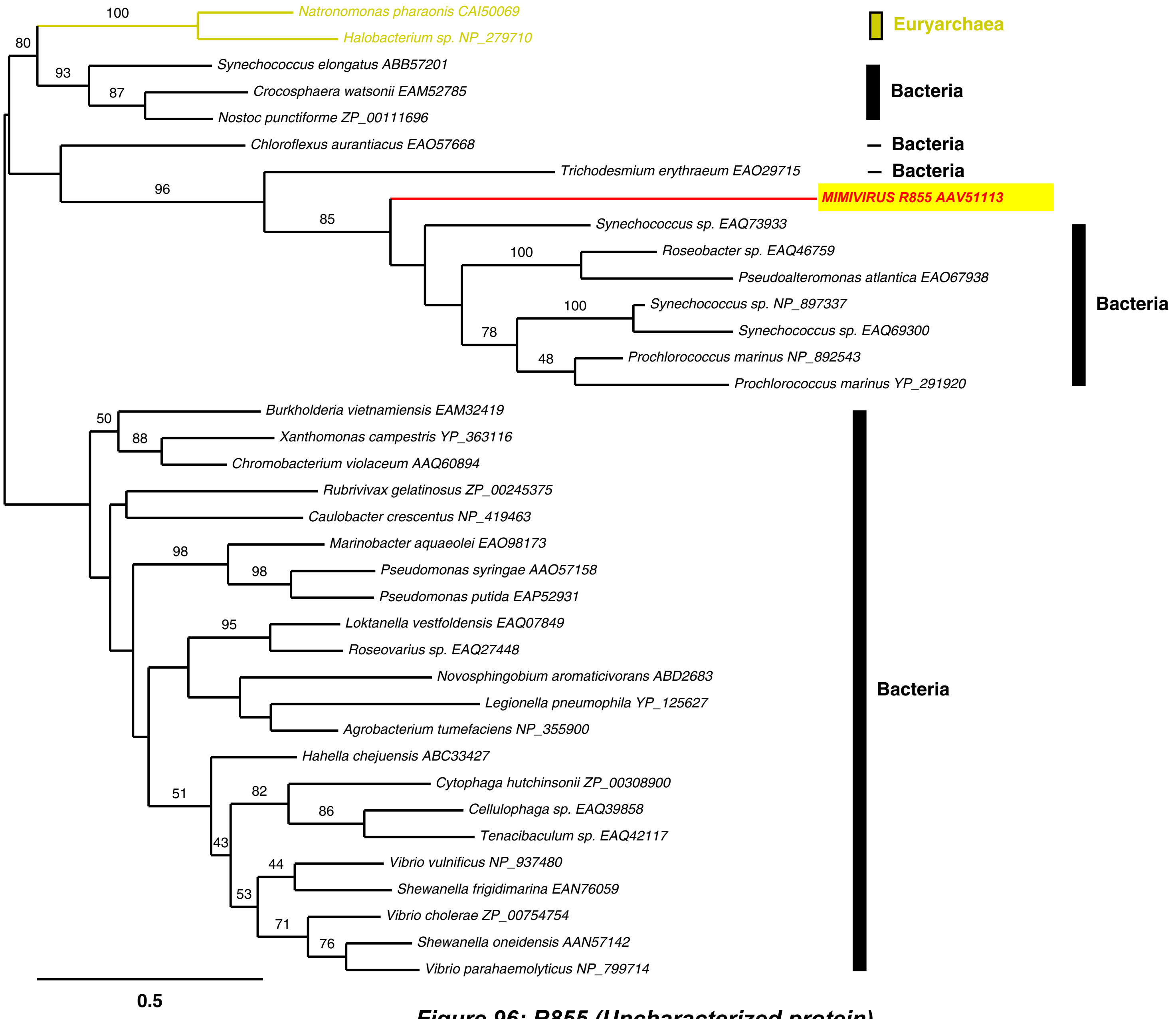

Figure 96: R855 (Uncharacterized protein)
(37 sequences, 134 positions)

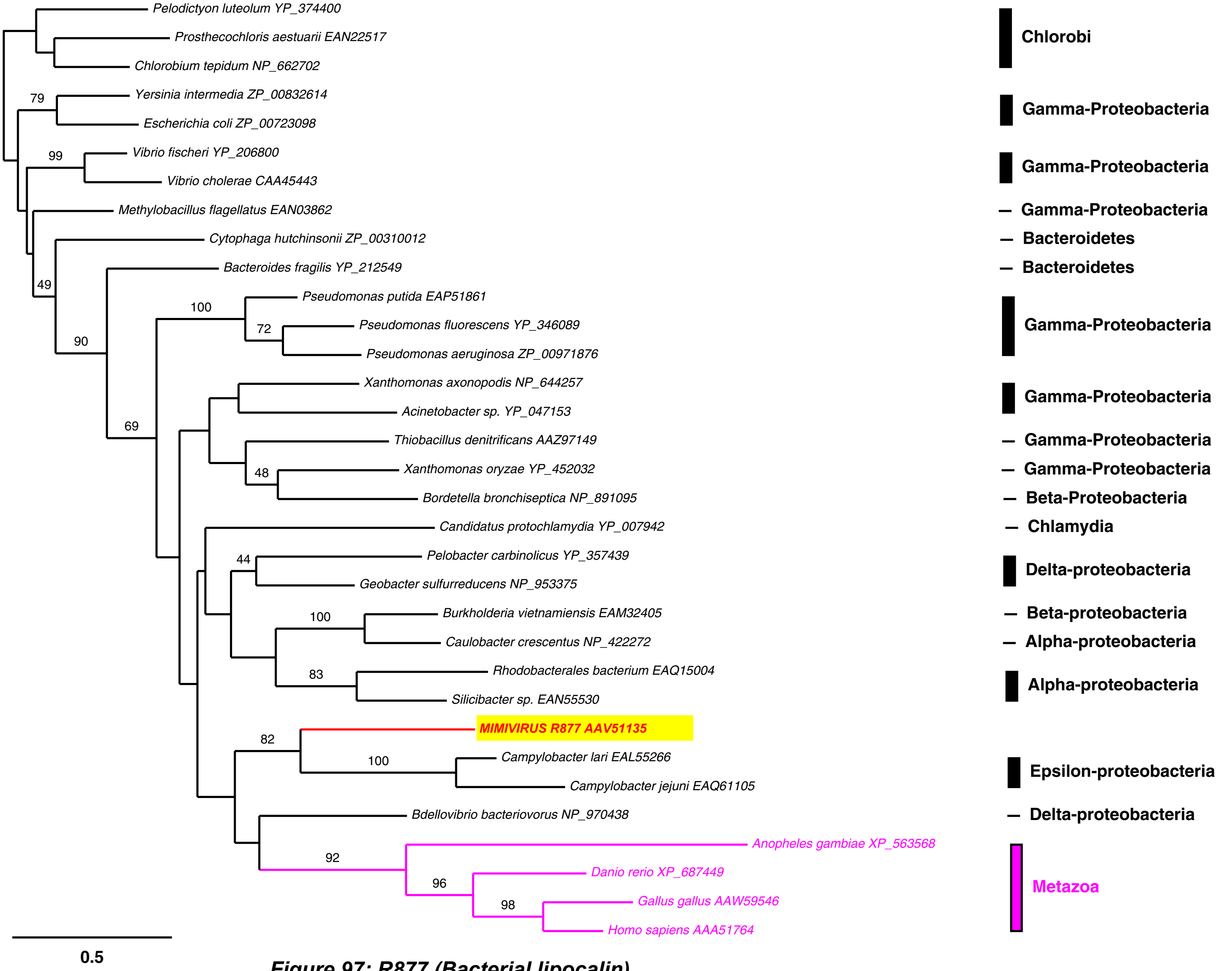

Figure 97: R877 (Bacterial lipocalin)

(33 sequences, 114 positions)

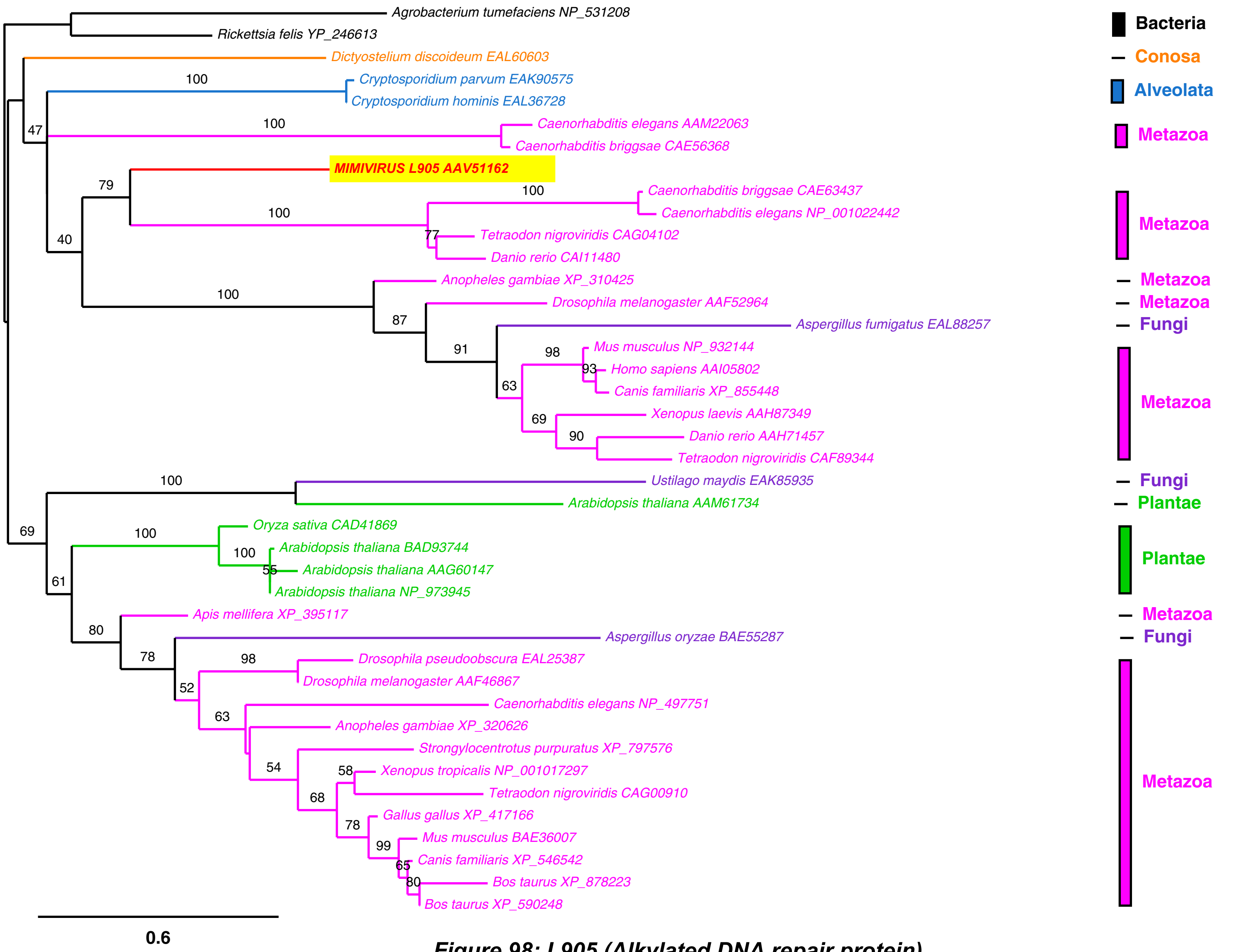

Figure 98: L905 (Alkylated DNA repair protein)

(41 sequences, 94 positions)
